# Supplementary material for: Cerebral organoids expressing mutant actin genes reveal cellular mechanism underlying microcephaly
Source: EMBO Rep. 2025 Dec 10;27(2):387–415. doi: 10.1038/s44319-025-00647-7 (PMC12852704; doi:10.1038/s44319-025-00647-7)
Supplement: Supplementary file 1 — Appendix [file 44319_2025_647_MOESM1_ESM.pdf]

Appendix

Table of contents

Appendix Figure S1	page 2
Appendix Figure S2	page 3
Appendix Figure S3	page 5
Appendix Figure S4	page 7
Appendix Figure S5	page 9
Appendix Figure S6	page 11
Appendix Figure S7	page 12
Appendix Figure S8	page 14
Appendix Figure S9	page 16
Appendix Figure S10	page 18
Appendix Figure S11	page 20
Appendix Figure S12	page 22
Appendix Figure S13	page 23

Appendix Figure S1.

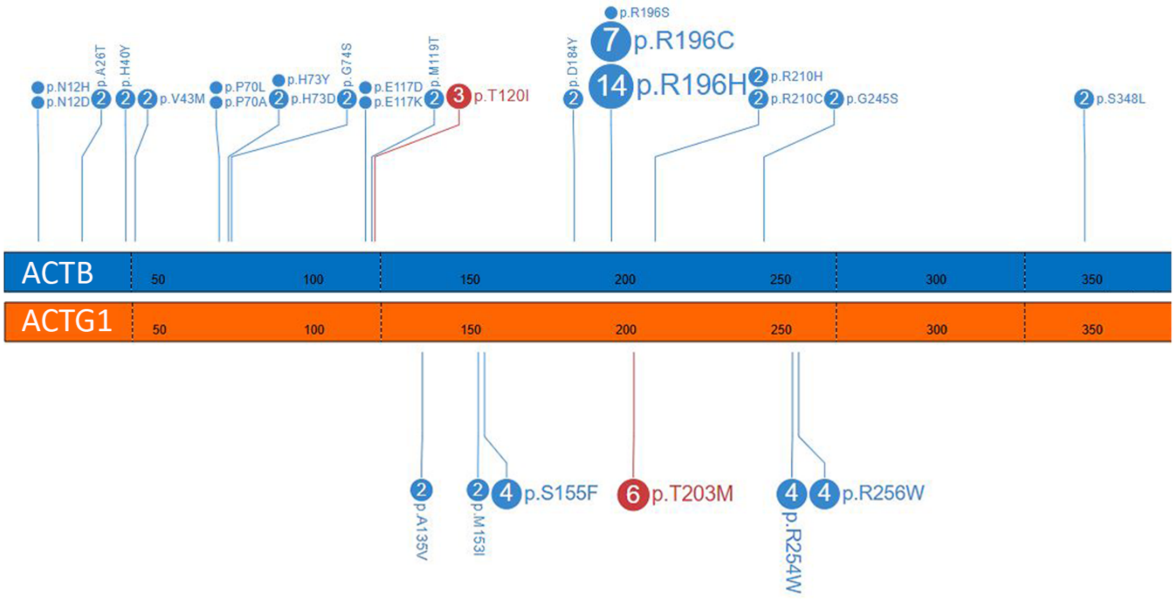

Appendix Figure S1. Overview of pathogenic variants in the genes *ACTB* and *ACTG1* associated with BWCFF-S.

Schematic depiction of the genes *ACTB* (blue) and *ACTG1* (orange) with codon numbers and the position of the pathogenic missense variants associated with BWCFF-S shown above (*ACTB*) or below (*ACTG1*). The figure presents only recurrent variants found in more than two patients for a more concise overview. The number within the circles indicates the number of affected patients. Red circles mark the pathogenic variants analyzed in this study (created with ProteinPaint).

Appendix Figure S2.

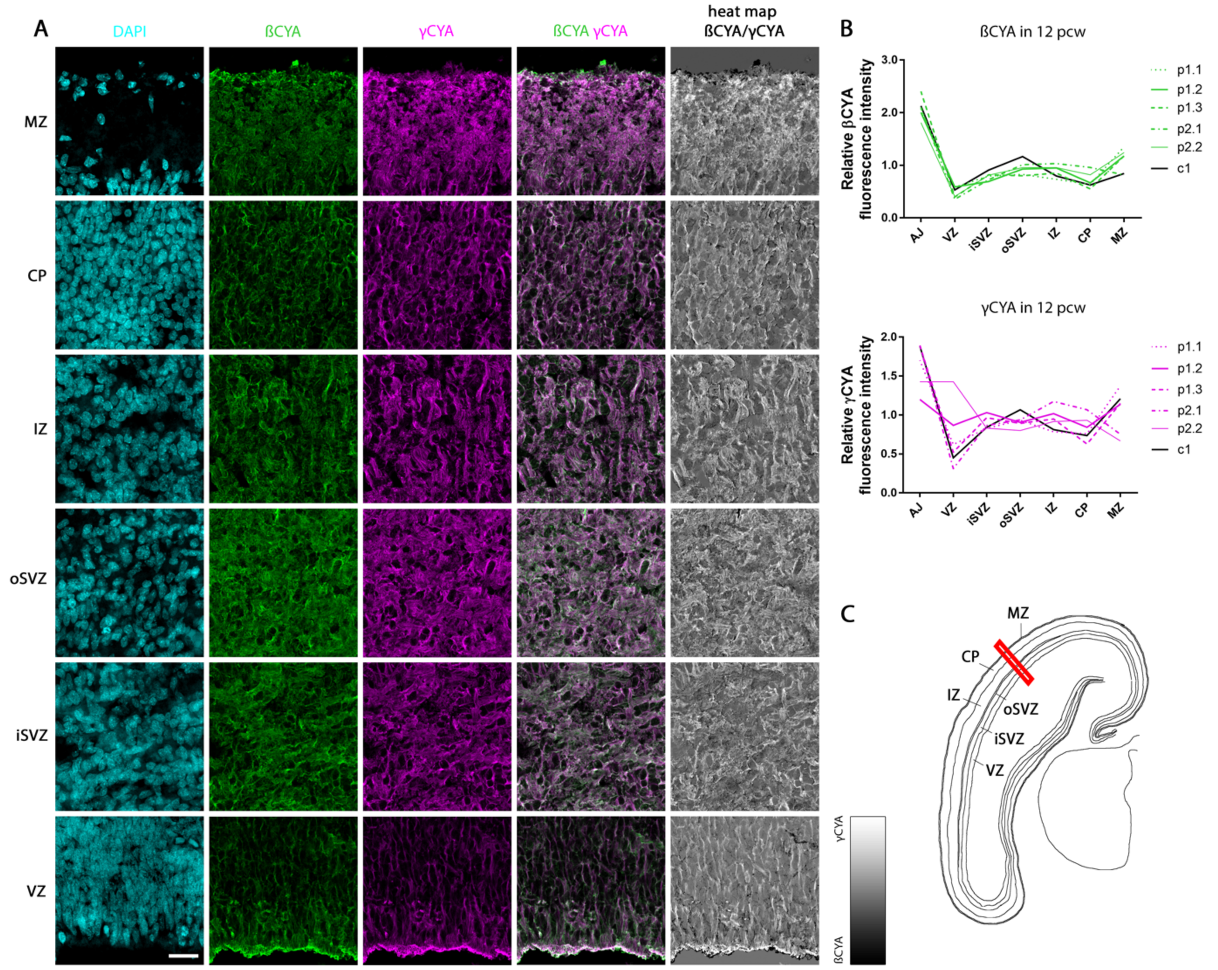

Appendix Figure S2. Widespread distribution of βCYA and γCYA in the fetal human neocortex at 12 pcw.

(A) Double immunofluorescence for βCYA (green) and γCYA (magenta), combined with DAPI staining (blue), of a coronal 12 pcw fetal human neocortex cryosection. Representative images of the indicated six zones of the developing cortical wall are depicted; iSVZ, inner subventricular zone; oSVZ, outer subventricular zone; IZ, intermediate zone; CP, cortical plate; MZ, marginal zone. The right column shows a heat map of βCYA (black) and γCYA (white) immunoreactivity. Scale bar, 25 µm. **(B)** Quantification of the relative fluorescence intensities for βCYA (green, upper panel) and γCYA (magenta, lower panel) across the indicated zones of the cortical wall (AJ, adherens junction belt), using sections of paraffin-embedded 12 pcw fetal human brains from two individuals (p1, p2) and of a cryoprotected 12

pcw fetal human brain sample of one individual (c1); for p1 three sections (p1.1-p1.3), for p2 two sections (p2.1-p2.2) and for c1 one section were analyzed. The section (c1), which is shown in (**A**), is indicated in black. (**C**) Schematic overview of a coronal 12 pcw fetal human neocortex section indicating the region (red rectangle) shown in (**A**).

Appendix Figure S3.

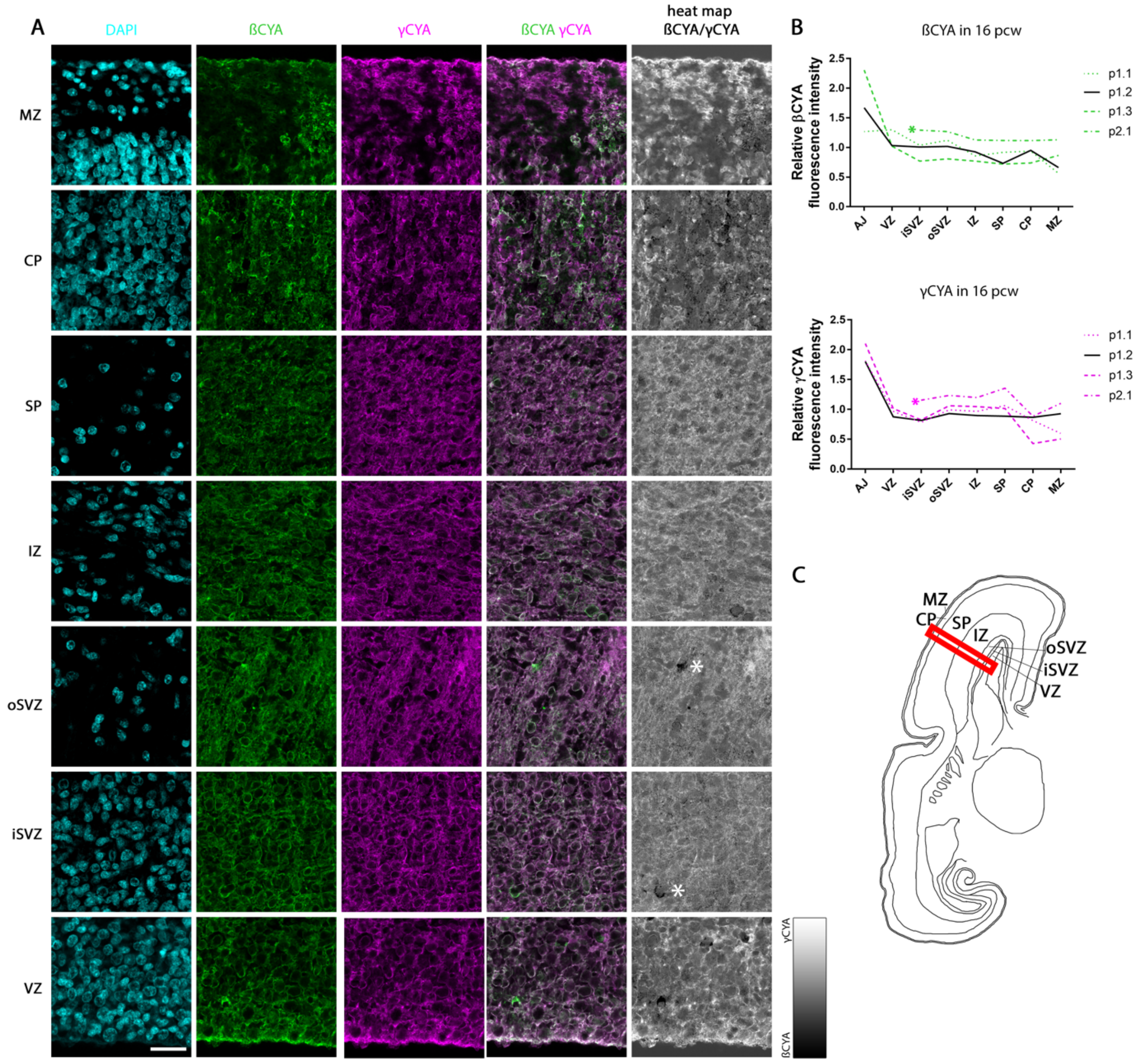

Appendix Figure S3. Widespread distribution of βCYA and γCYA in the developing fetal human neocortex at 16 pcw.

(**A**) Double immunofluorescence for βCYA (green) and γCYA (magenta), combined with DAPI staining (blue), of a coronal 16 pcw fetal human neocortex paraffin section. Representative images of the indicated seven zones of the developing cortical wall are depicted; iSVZ, inner subventricular zone; oSVZ, outer subventricular zone; IZ, intermediate zone; SP, subplate; CP, cortical plate; MZ, marginal zone. The right column shows a heat map of βCYA (black) and γCYA (white) immunoreactivity. Scale bar, 25 µm. Asterisks indicate imaging artefacts. (**B**) Quantification of the relative fluorescence intensities for βCYA (green, upper panel) and γCYA (magenta, lower panel) across the indicated zones of the cortical wall

(AJ, adherens junction belt), using sections of paraffin-embedded fetal human brains from two individuals (p1, p2); for p1 three sections (p1.1-p1.3) and for p2, one section (p2.1) were analyzed. The section (p1.2), which is shown in (**A**) is indicated in black. Note that AJ and VZ of individual p2 could not be evaluated due to the quality of the section. (**C**) Schematic overview of a coronal 16 pcw fetal human neocortex section indicating the region (red rectangle) shown in (**A**).

Appendix Figure S4.

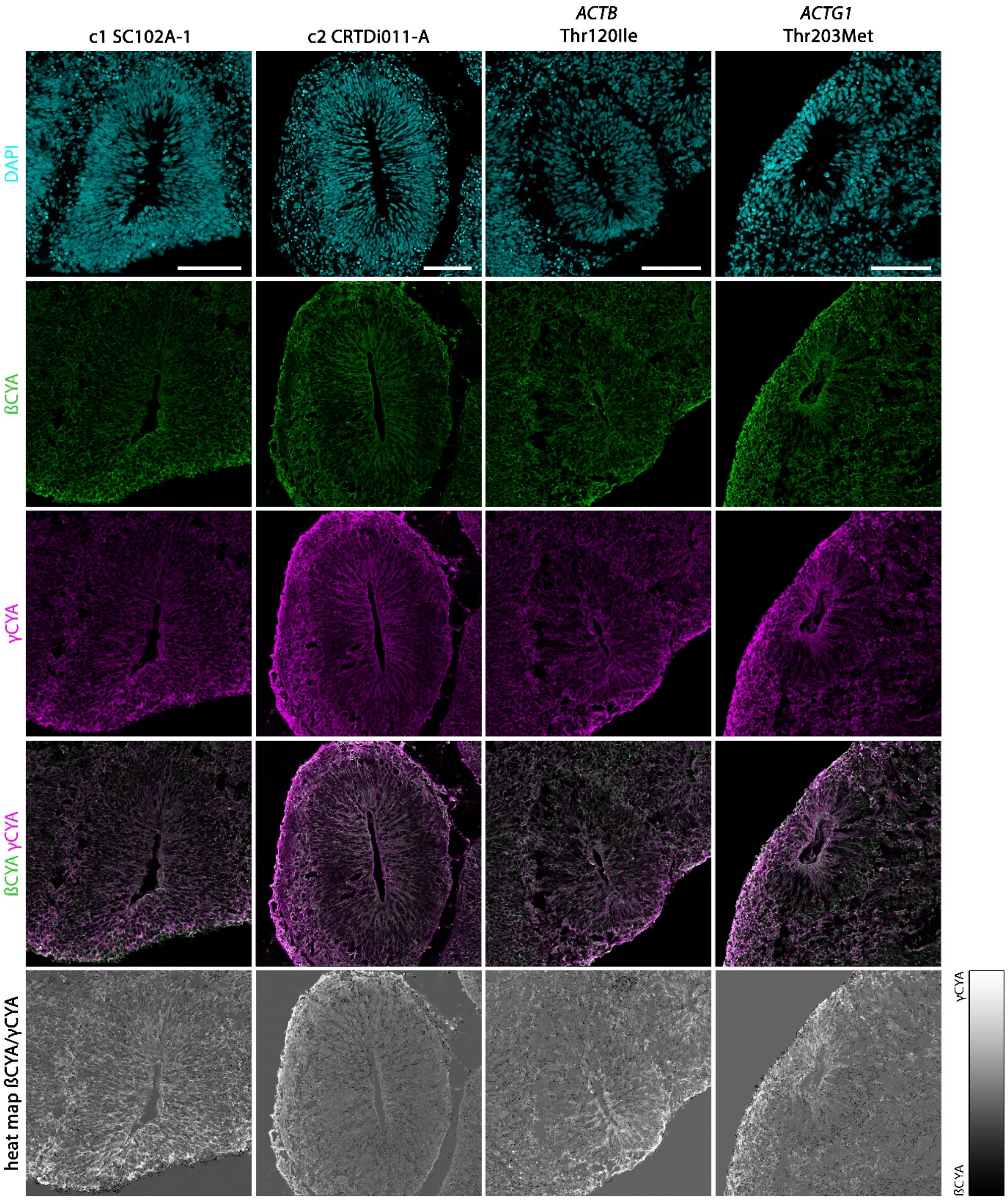

Appendix Figure S4. Widespread distribution of βCYA and γCYA in control and BWCFF-S cerebral organoids.

Double immunofluorescence for βCYA (green) and γCYA (magenta), combined with DAPI staining (blue), of sections of control (c1, SC102A-1 and c2, CRTDi011-A; two left columns), BWCFF-S *ACTB* Thr120Ile (second column from right) and BWCFF-S *ACTG1* Thr203Met (right column) 30-days old cerebral organoids showing ventricle-like structures. The bottom

row shows a heat map of βCYA (black) and γCYA (white) immunoreactivity. Scale bars, 100 μm.

Appendix Figure S5.

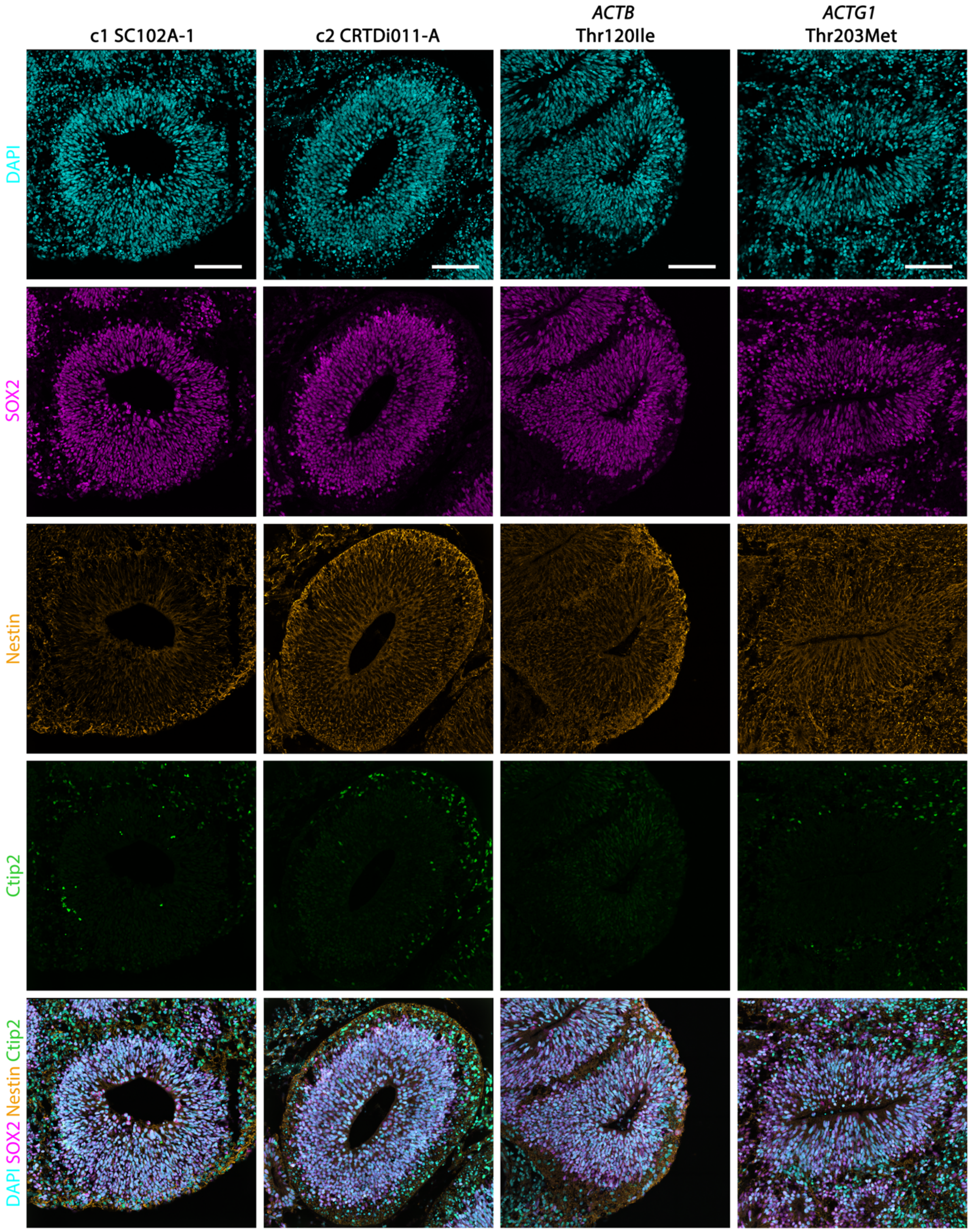

Appendix Figure S5. Cell type composition of control and BWCFF-S cerebral organoids – analysis 1.

Triple immunofluorescence for SOX2 (magenta), nestin (orange) and Ctip2 (green), combined with DAPI staining (blue), of sections of control (c1, SC102A-1 and c2,

CRTDi011-A; two left columns), BWCFF-S *ACTB* Thr120Ile (second column from right) and BWCFF-S *ACTG1* Thr203Met (right column) 30-days old cerebral organoids showing ventricle-like structures. Scale bars, 100 µm.

Appendix Figure S6.

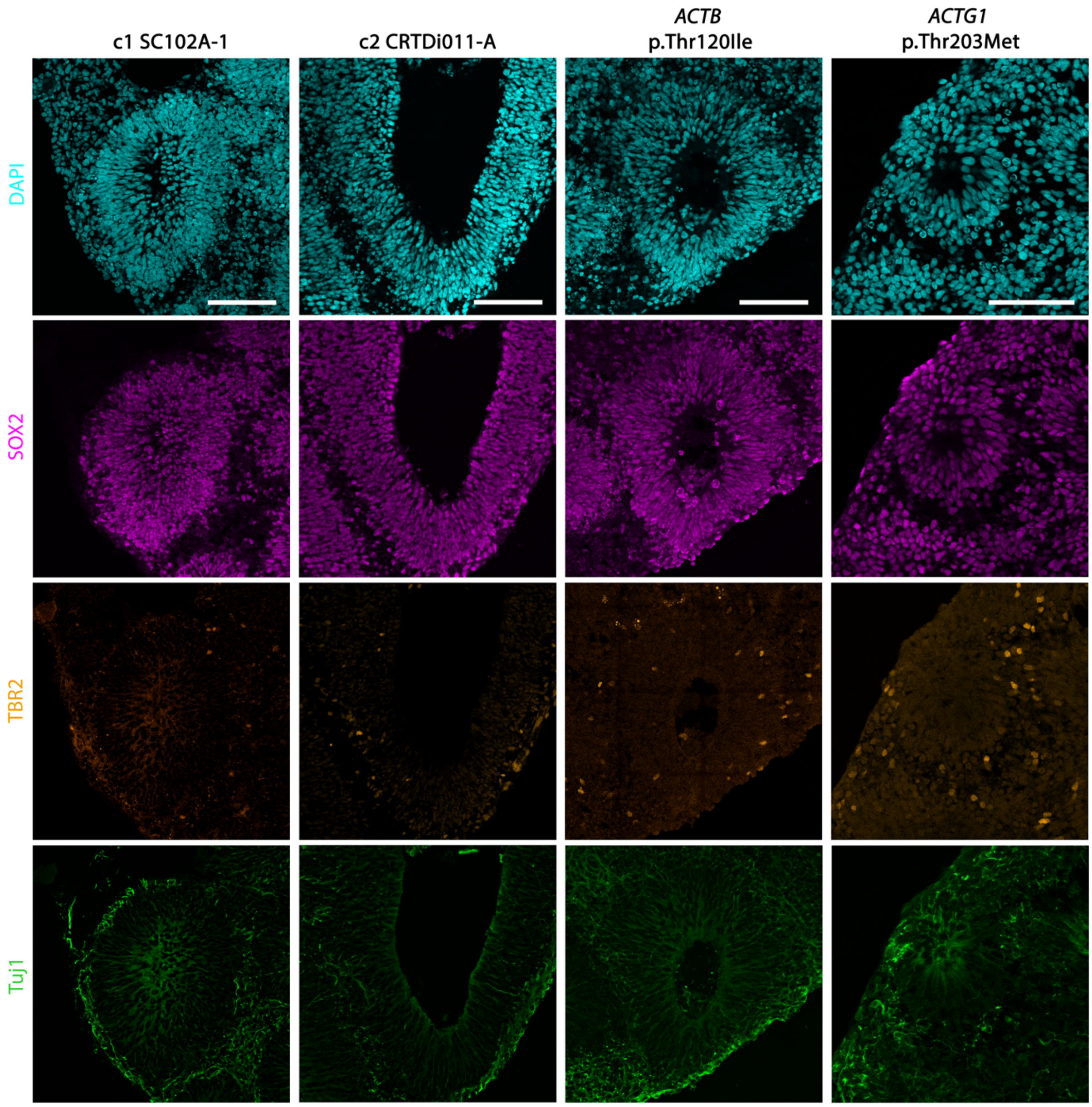

Appendix Figure S6. Cell type composition of control and BWCFF-S cerebral organoids – analysis 2.

Triple immunofluorescence for SOX2 (magenta), TBR2 (orange) and Tuj1 (green), combined with DAPI staining (blue), of control (c1, SC102A-1 and c2, CRTDi011-A; two left columns), BWCFF-S *ACTB* Thr120Ile (second column from right) and BWCFF-S *ACTG1* Thr203Met (right column) 30-days old cerebral organoids showing ventricle-like structures. Scale bars, 100 µm.

Appendix Figure S7.

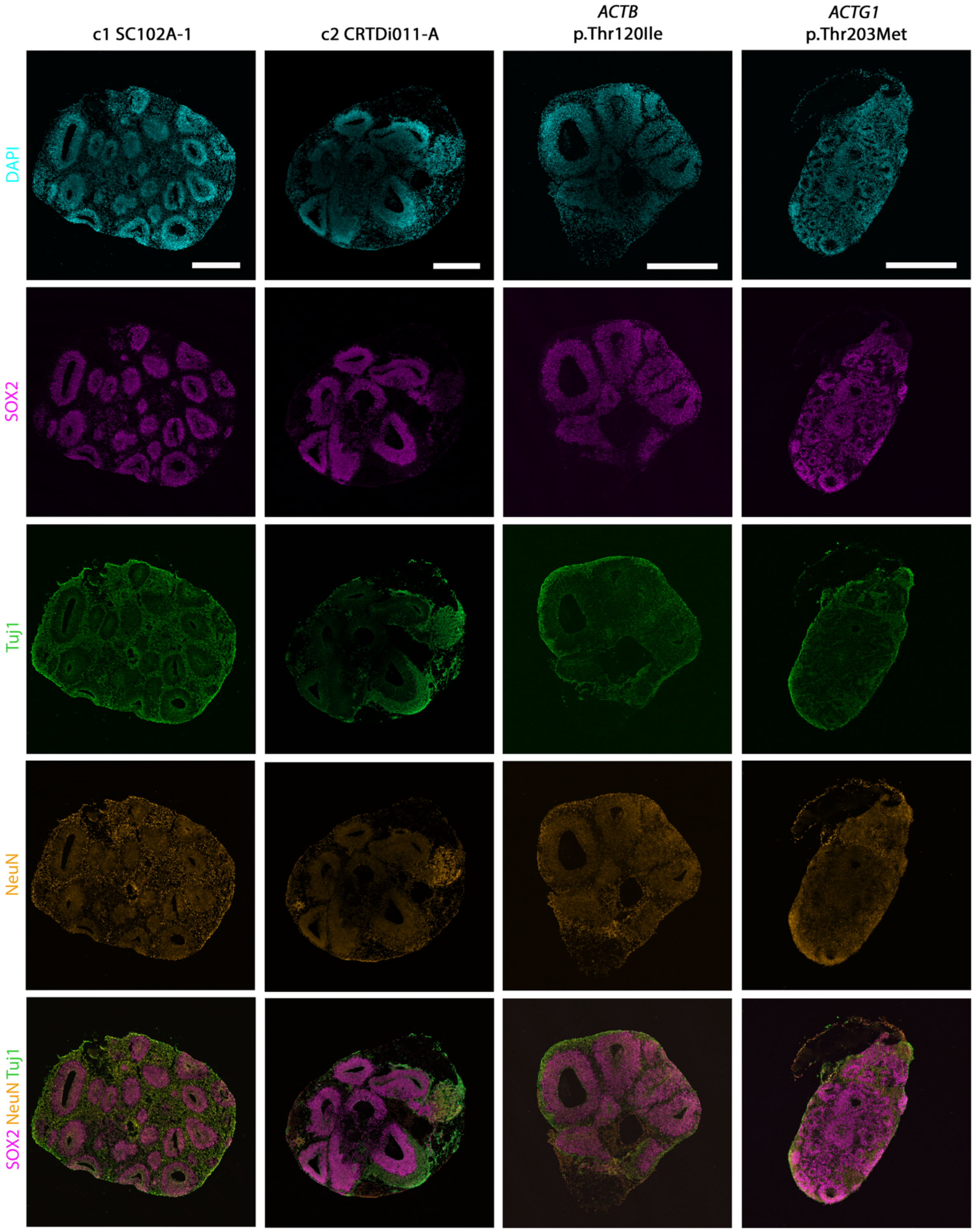

Appendix Figure S7. Overview images of control and BWCFF-S cerebral organoids.

Triple immunofluorescence for SOX2 (magenta), TUJ1 (green) and NeuN (organge), combined with DAPI staining (blue), of sections of control (c1, SC102A-1 and c2, CRTDi011-A; two left

columns), BWCFF-S *ACTB* Thr120Ile (second column from right) and BWCFF-S *ACTG1* Thr203Met (right column) 30-days old cerebral organoids. Scale bars, 500 μm.

Appendix Figure S8.

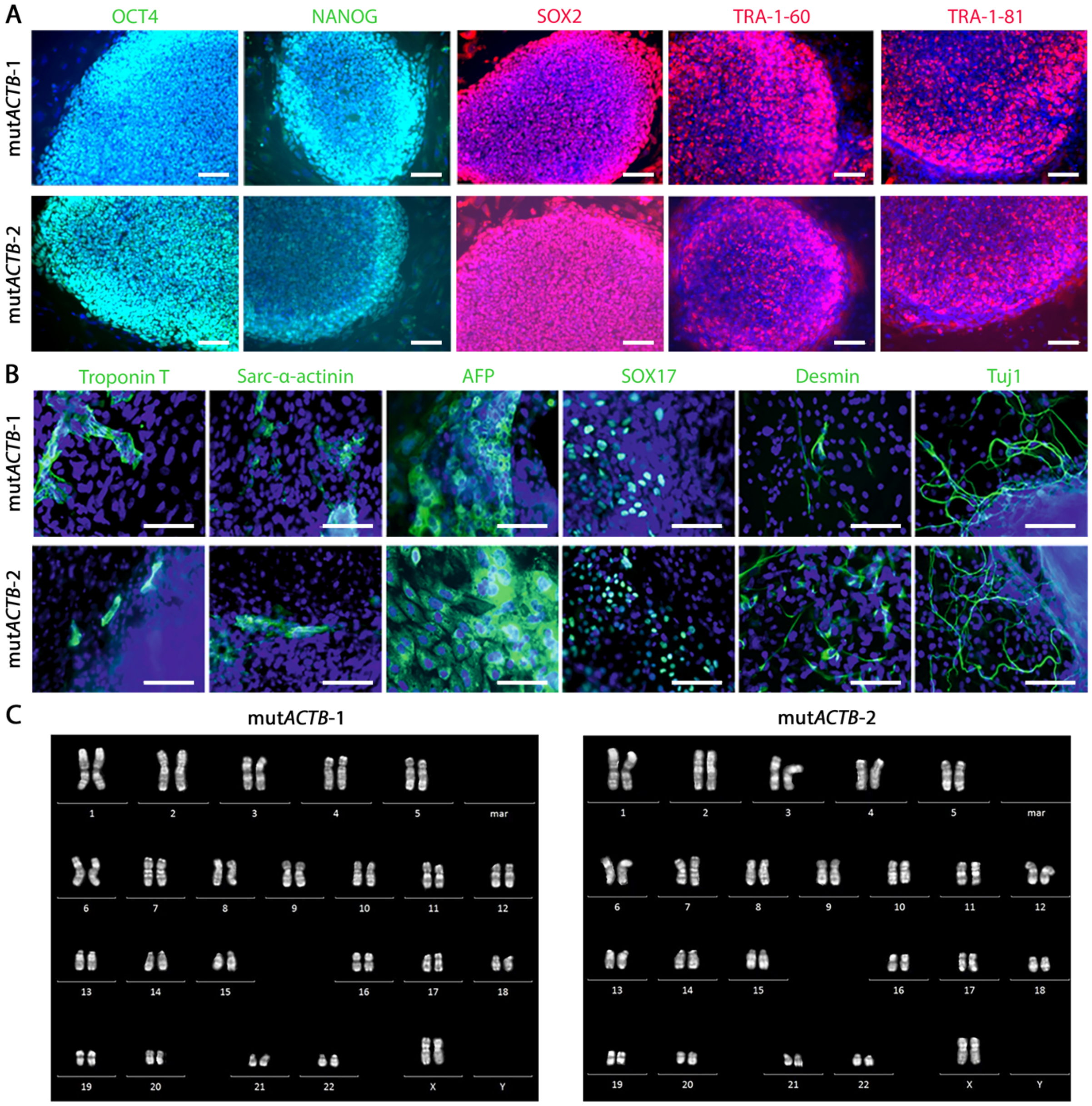

Appendix Figure S8. Characterization of the BWCFF-S *ACTB* Thr120Ile patient-derived iPSC clones mut*ACTB*-1 and mut*ACTB*-2.

(**A**) Immunofluorescence for either OCT4 (green, first column from left), NANOG (green, second column from left), SOX2 (magenta, third column from left), TRA-1-60 (magenta, fourth column from left), or TRA-1-81 (magenta, fifth column from left), combined with DAPI staining (blue), of BWCFF-S *ACTB* Thr120Ile patient-derived iPSC clones mut*ACTB*-1 (upper row) and mut*ACTB*-2 (lower row). Scale bars, 100 µm. (**B**) Immunofluorescence for either Troponin T (green, first column from left), Sarc-α-actinin (green, second column from left),

AFP (green, third column from left), SOX17 (green, fourth column from left), Desmin (green, fifth column from left), or Tuj1 (green, sixth column from left), combined with DAPI staining (blue), of BWCFF-S *ACTB* Thr120Ile patient-derived iPSC clones mut*ACTB*-1 (upper row) and mut*ACTB*-2 (lower row). Scale bars, 100 µm. (**C**) Karyotype analysis of BWCFF-S *ACTB* Thr120Ile patient-derived iPSC clones mut*ACTB*-1 (left) and mut*ACTB*-2 (right).

Appendix Figure S9.

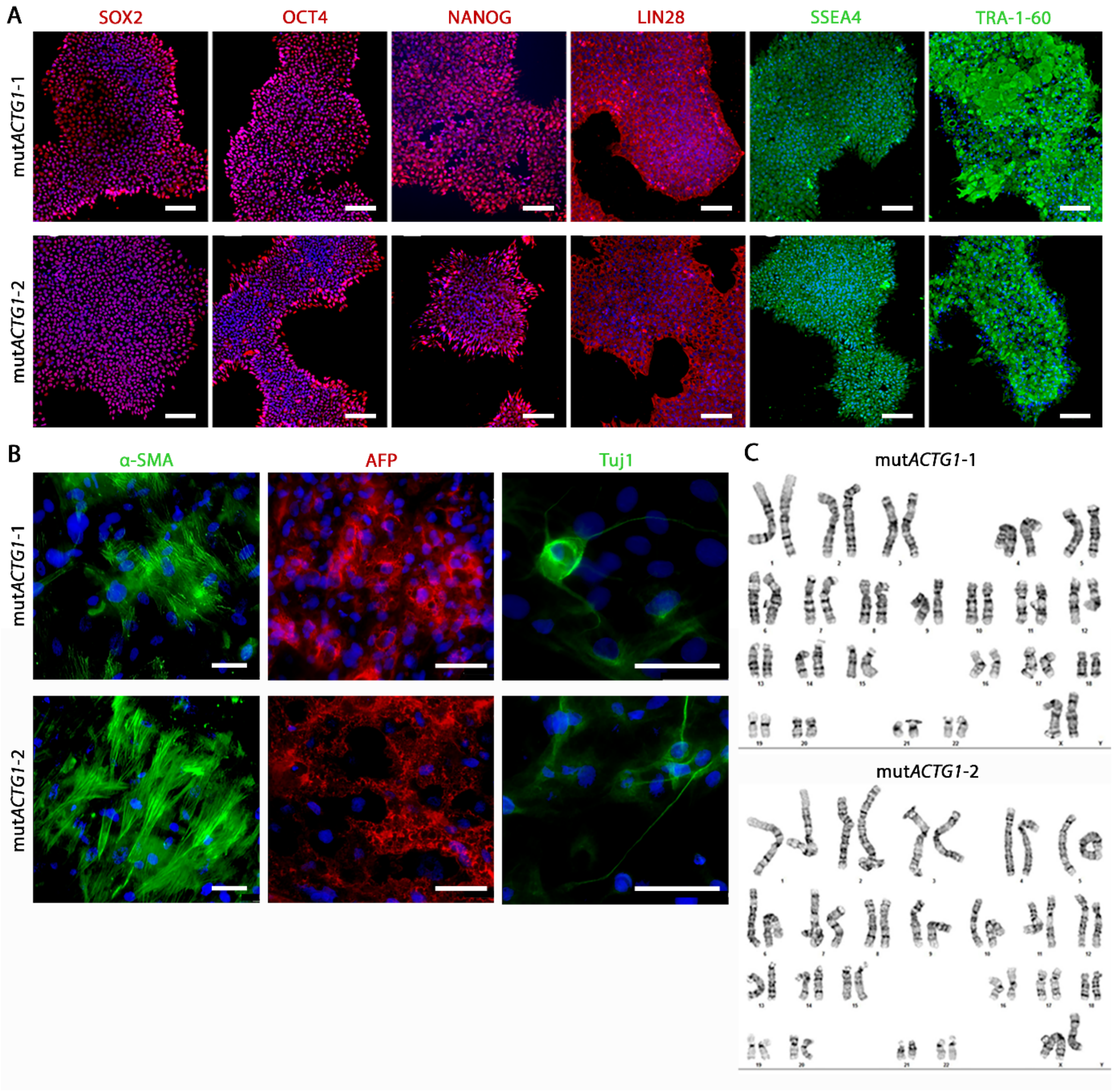

Appendix Figure S9. Characterization of the BWCFF-S *ACTG1* Thr203Met patient-derived iPSC clones mut*ACTG1*-1 and mut*ACTG1*-2.

(**A**) Immunofluorescence for either SOX2 (magenta, first column from left), OCT4 (magenta, second column from left), NANOG (magenta, third column from left), LIN28 (magenta, fourth column from left), SSEA4 (green, fifth column from left), or TRA-1-60 (green, sixth column from left), combined with DAPI staining (blue), of BWCFF-S *ACTG1* Thr203Met patient-derived iPSC clones mut*ACTG1*-1 (upper row) and mut*ACTG1*-2 (lower row). Scale bars, 100 μm. (**B**) Immunofluorescence for either α-SMA (green, left column), AFP (magenta, middle

column), or Tuj1 (green, right column), combined with DAPI staining (blue), of BWCFF-S *ACTG1* Thr203Met patient-derived iPSC clones mut*ACTG1*-1 (upper row) and mut*ACTG1*-2 (lower row). Scale bars, 50 µm. (**C**) Karyotype analysis of BWCFF-S *ACTG1* Thr203Met patient-derived iPSC clones mut*ACTG1*-1 (upper panel) and mut*ACTG1*-2 (lower panel).

Appendix Figure S10.

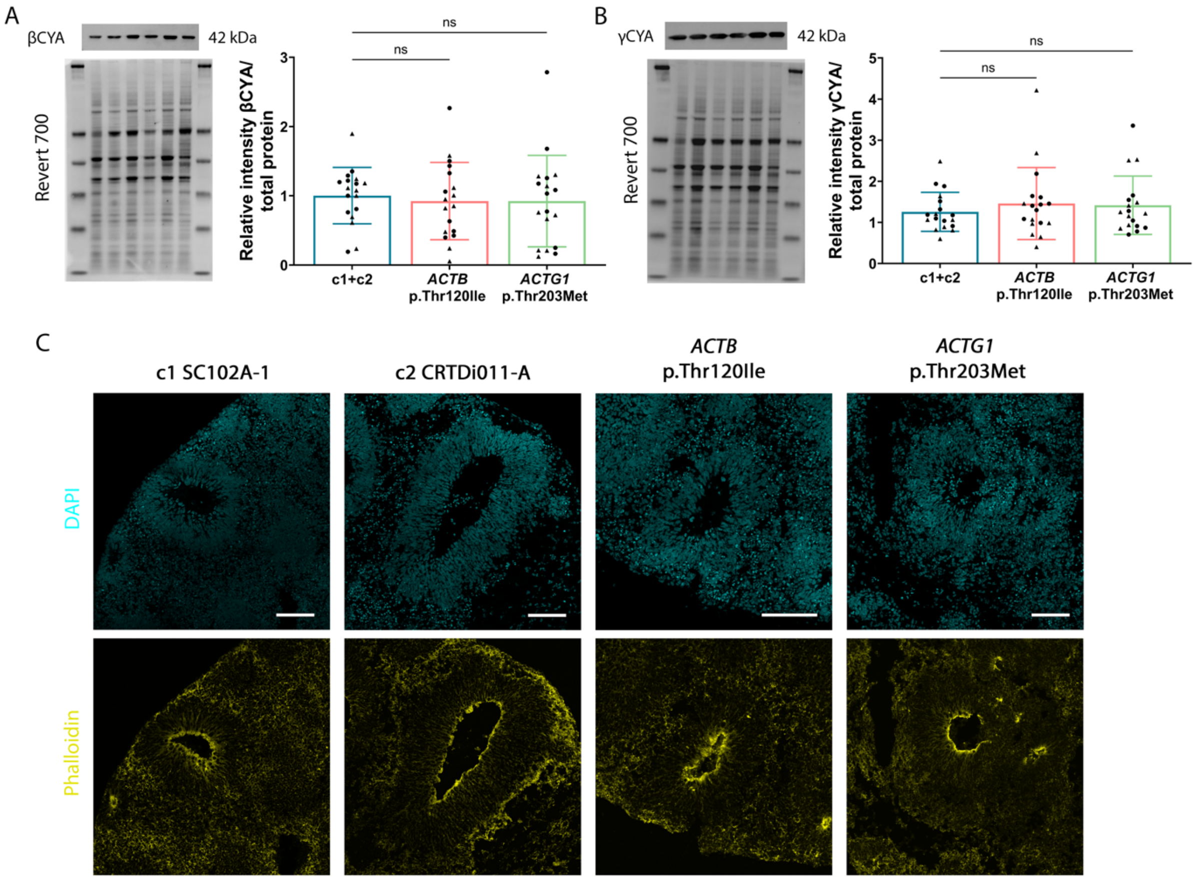

Appendix Figure S10. Western blot analysis and phalloidin staining do not show changes in actin levels between control, BWCFF-S cerebral organoids.

(**A**,**B**) Left in each panel, representative Western blot images showing either βCYA (**A**)or γCYA (**B**) levels in cerebral organoids generated from either the control cell lines SC102A-1 and CRTDi011-A or from the BWCFF-S *ACTB* Thr120Ile (two different iPSC clones), and BWCFF-S *ACTG1* Thr203Met (two different iPSC clones) organoids. Upper part, image section of whole blot image of either βCYA (**A**)or γCYA (**B**) bands. Right in each panel, quantification of band intensity, normalized to total protein of control (c1, SC102A-1 and c2, CRTDi011-A; blue bars), BWCFF-S *ACTB* Thr120Ile (red bars), and BWCFF-S *ACTG1* Thr203Met (green bars) cerebral organoids at culture day 30. Data are the mean of 18 control (generated from two different iPSC lines; indicated by circles (c1, SC102A-1) and triangles (c2, CRTDi011-A)), 18 BWCFF-S *ACTB* Thr120Ile (generated from two different iPSC clones;

indicated by circles and triangles) and 18 BWCFF-S *ACTG1* Thr203Met (generated from two different iPSC clones; indicated by circles and triangles) 30 days-old cerebral organoids of 3 independent batches; error bars indicate SD; ns, not significant (one-way ANOVA). (**C**) Phalloidin staining (yellow), combined with DAPI staining (blue), of sections of control (c1, SC102A-1 and c2, CRTDi011-A; two left columns), BWCFF-S *ACTB* Thr120Ile (second column from right) and BWCFF-S *ACTG1* Thr203Met (right column) 30-days old cerebral organoids showing ventricle-like structures. Scale bars, 100 µm.

Appendix Figure S11.

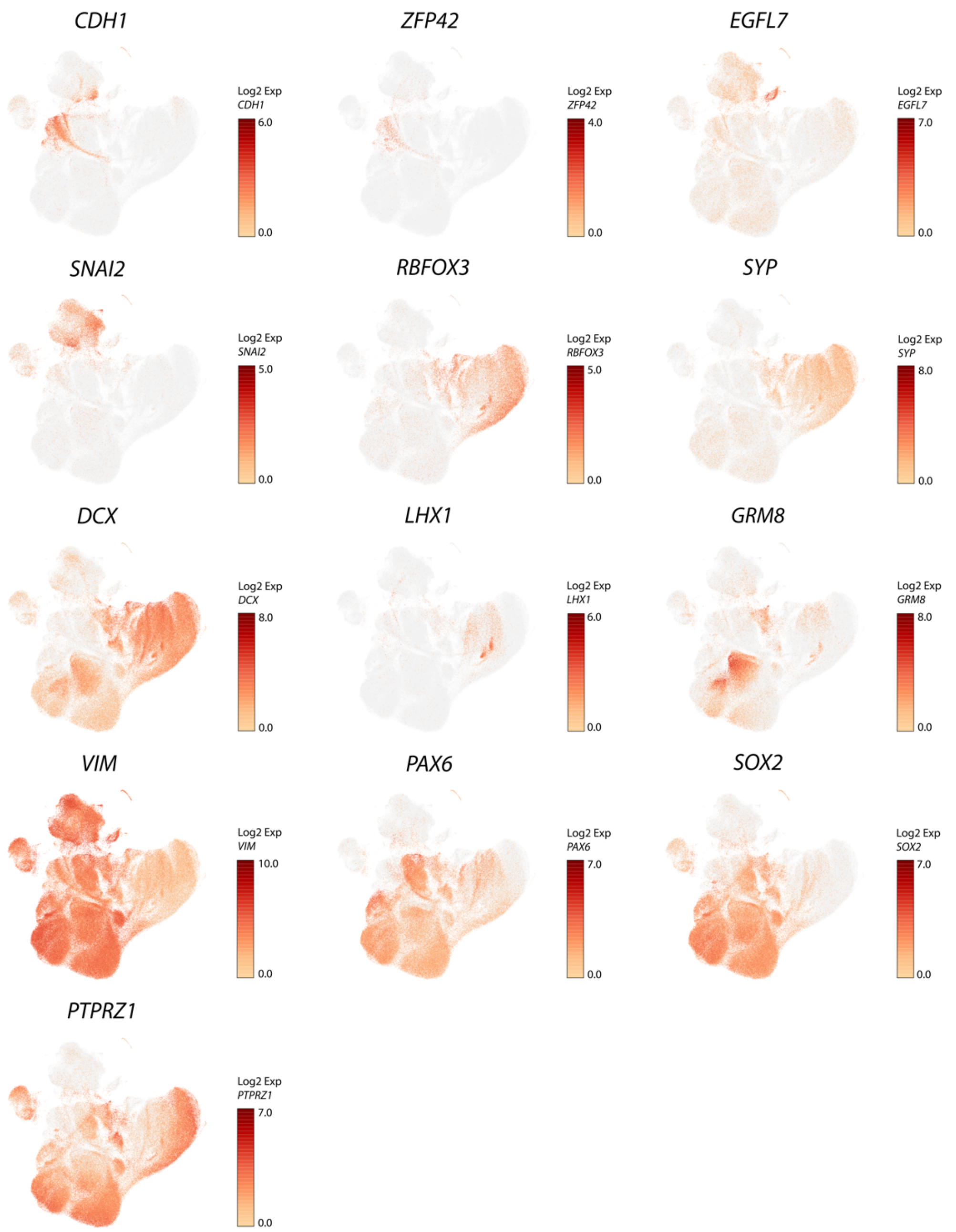

Appendix Figure S11. Examples of the expression of genes used to determine the different clusters in the scRNA-seq data.

UMAP plots of single-cell RNA-seq data from control (generated from two different iPSC lines; SC102A-1 and CRTDi011-A)), BWCFF-S *ACTB* Thr120Ile (generated from two different iPSC clones; mut*ACTB*-1 and mut*ACTB*-2), BWCFF-S *ACTG1* Thr203Met (generated from two different iPSC clones; mut*ACTG1*-1 and mut*ACTG1*-2), and BWCFF-S–like *ACTB* Thr120Ile (generated from the CRISPR/Cas9-edited CRTDi011-A–derived iPSC clones CRTDi011-A–

mut*ACTB*-1 and CRTDi011-A–mut*ACTB*-2, collectively referred to as cr. *ACTB* Thr120Ile). Each point represents a single cell, colored by the expression level of the indicated gene (legend on the right side of each UMAP plot).

Appendix Figure S12.

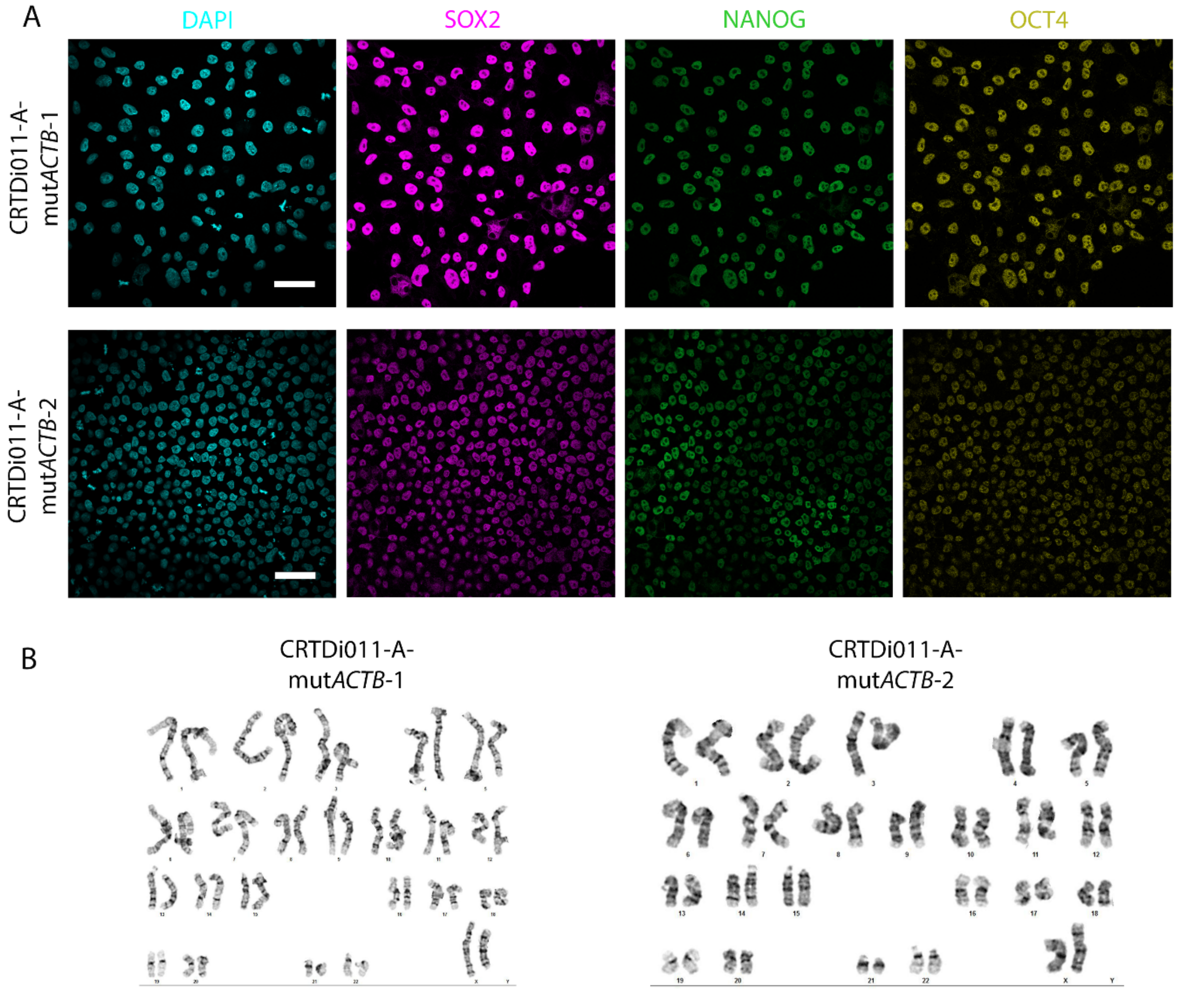

Appendix Figure S12. Characterization of the CRISPR/Cas9-edited CRTDi011-A–derived iPSC clones CRTDi011-A–mut*ACTB*-1 and CRTDi011-A–mut*ACTB*-2.

Triple immunofluorescence for SOX2 (magenta), NANOG (green) and OCT4 (yellow), combined with DAPI staining (blue), of CRISPR/Cas9-edited CRTDi011-A–derived iPSC clones CRTDi011-A–mut*ACTB*-1 (upper row) and CRTDi011-A–mut*ACTB*-2 (lower row). Scale bars, 50 µm. (B) Karyotype analysis of CRISPR/Cas9-edited CRTDi011-A–derived iPSC clones CRTDi011-A–mut*ACTB*-1 (left) and CRTDi011-A–mut*ACTB*-2 (right).

Appendix Figure S13.

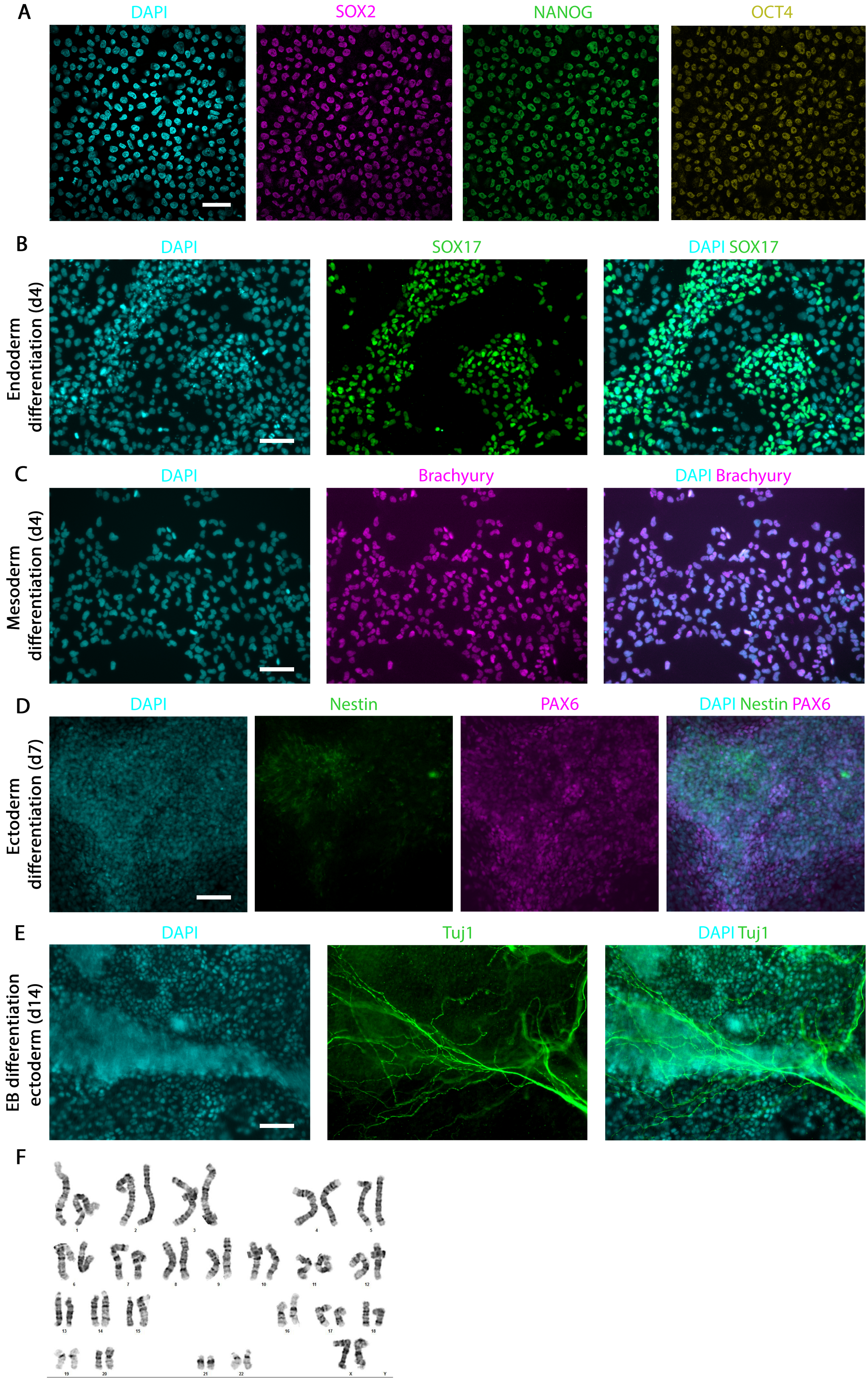

Appendix Figure S13. Characterization of the generated control iPSC line CRTDi011-A. (**A**) Triple immunofluorescence for SOX2 (magenta), NANOG (green) and OCT4 (yellow), combined with DAPI staining (blue), of CRTDi011-A iPSCs. Scale bars, 100 µm. (**B**,**C**) Immunofluorescence for either SOX17 (green, **B**) or Brachyury (magenta, **C**), in both cases combined with DAPI staining (blue), of cells after 4 days of either endoderm (**B**) or mesoderm (**C**) differentiation. Scale bars, 100 µm. (**D**) Double immunofluorescence for Nestin (green) and Pax6 (magenta), combined with DAPI staining (blue), of cells after 7 days of ectoderm differentiation. Scale bars, 100 µm. (**E**) Immunofluorescence for Tuj1 (green), combined with DAPI staining (blue), of embryoid bodies (EB) after 14 days of ectoderm differentiation. Scale bars, 100 µm. (**F**) Karyotype analysis of CRTDi011-A iPSCs.
