# Supplementary figures and images for: Cerebral organoids expressing mutant actin genes reveal cellular mechanism underlying microcephaly
Source: EMBO Rep. 2025 Dec 10;27(2):387–415. doi: 10.1038/s44319-025-00647-7 (PMC12852704; doi:10.1038/s44319-025-00647-7)

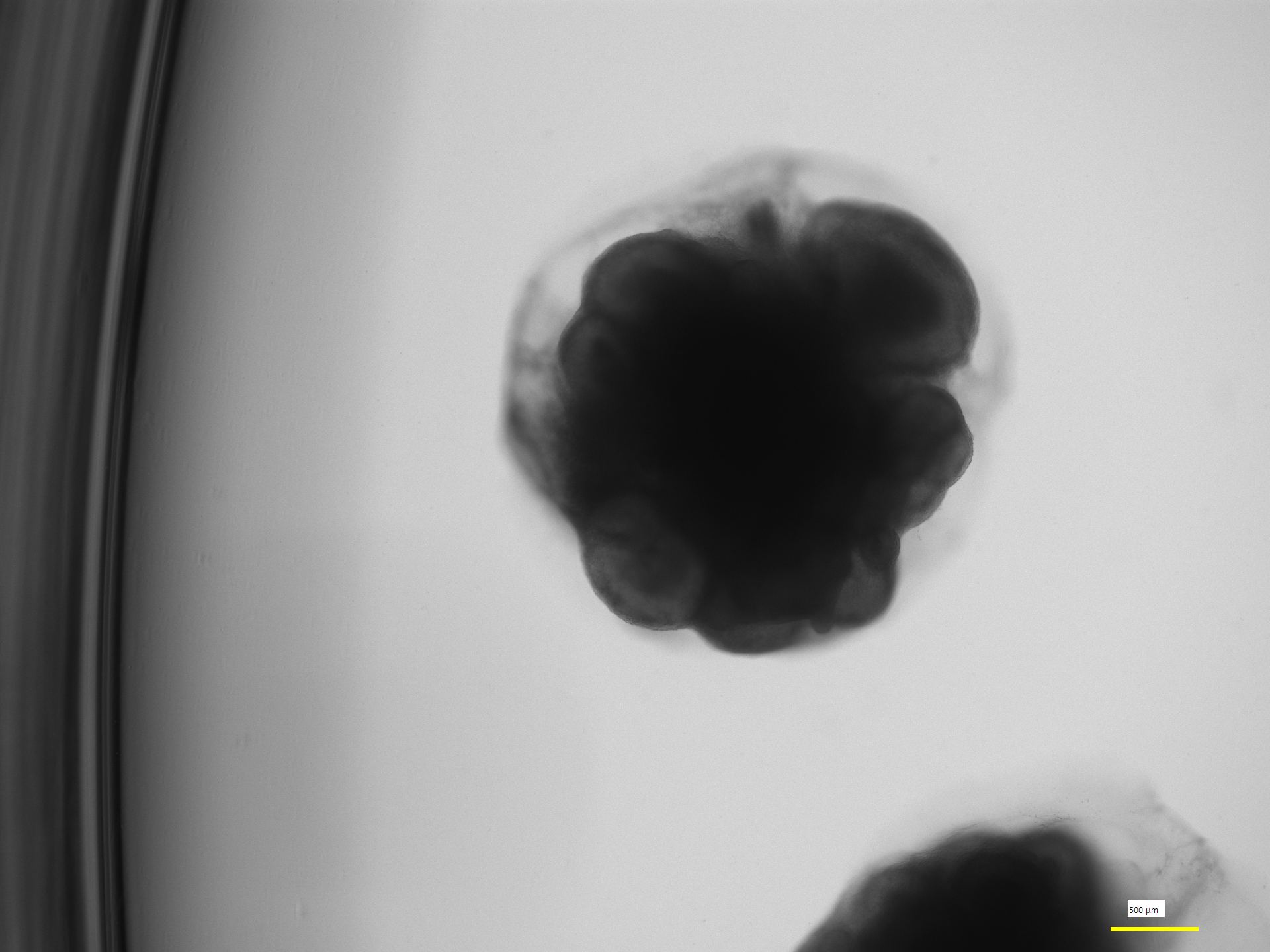

Supplement: Supplementary file 9 — Source data Fig. 2 [file 44319_2025_647_MOESM9_ESM.zip › Figure 2/2A/c1_SC102A-1_d29_brightfield.tif]

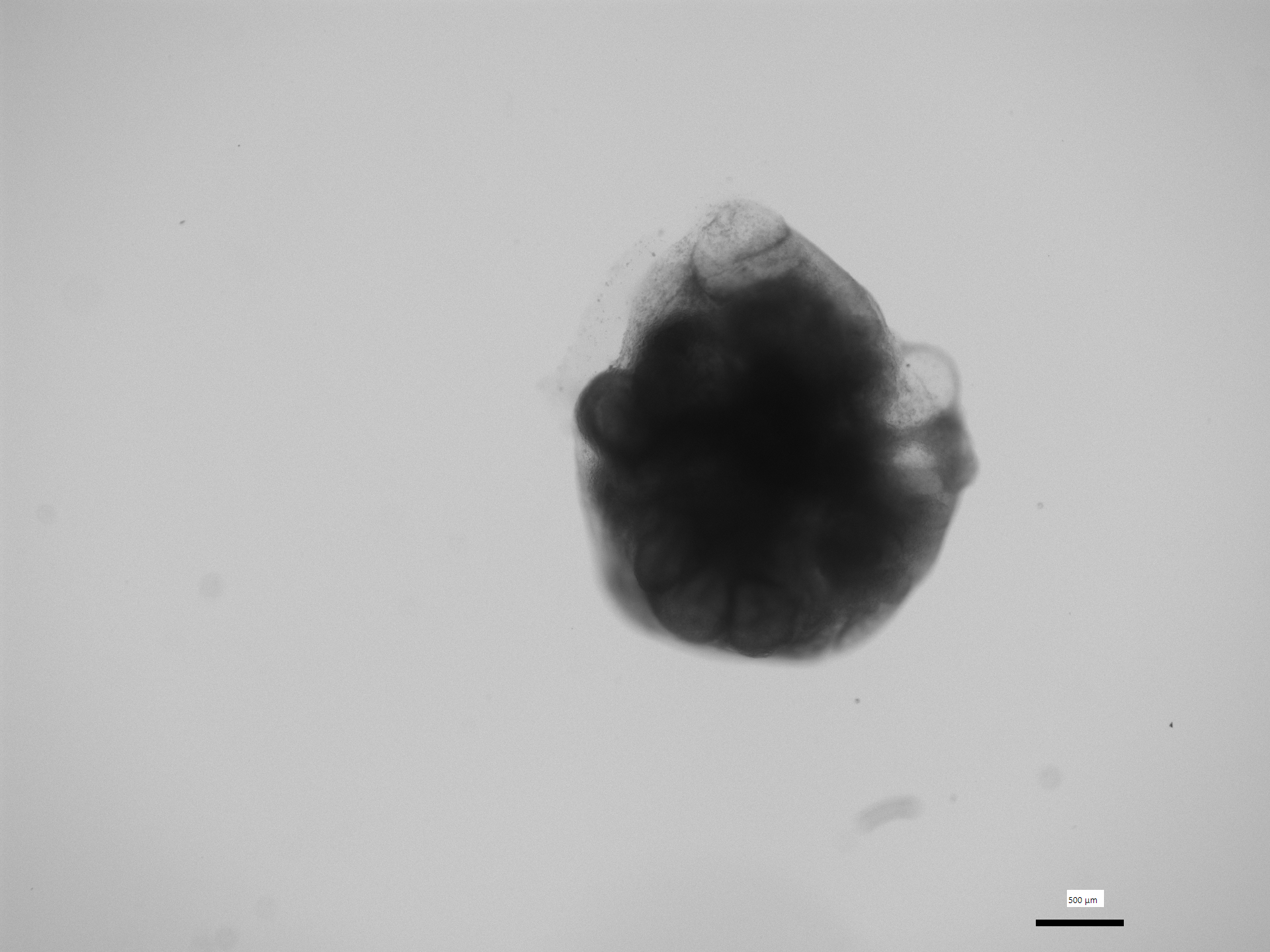

Supplement: Supplementary file 9 — Source data Fig. 2 [file 44319_2025_647_MOESM9_ESM.zip › Figure 2/2A/mutACTB-2_d29_brightfield.tif]

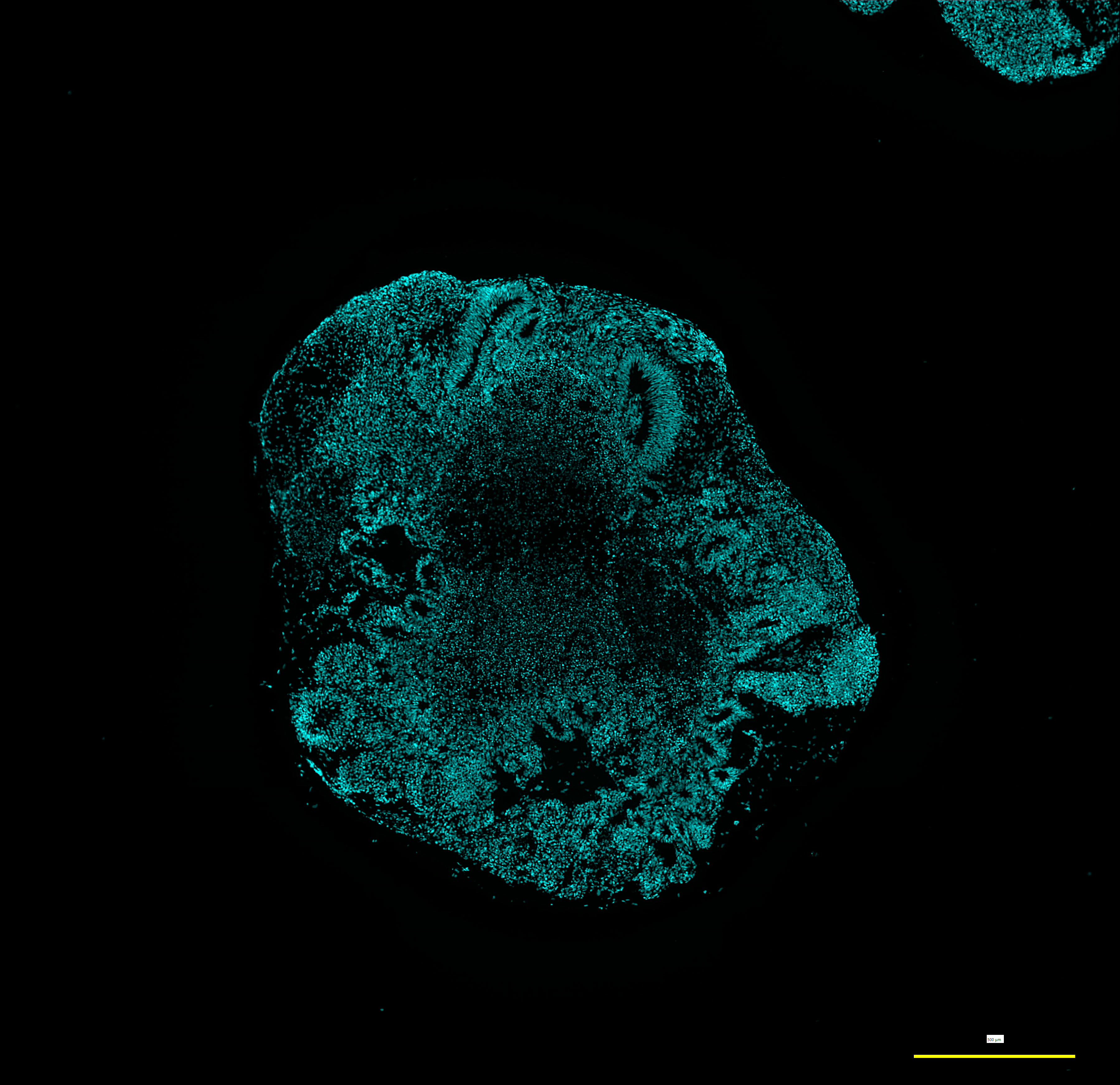

Supplement: Supplementary file 9 — Source data Fig. 2 [file 44319_2025_647_MOESM9_ESM.zip › Figure 2/2A/mutACTG1-1_d30_DAPI.tif]

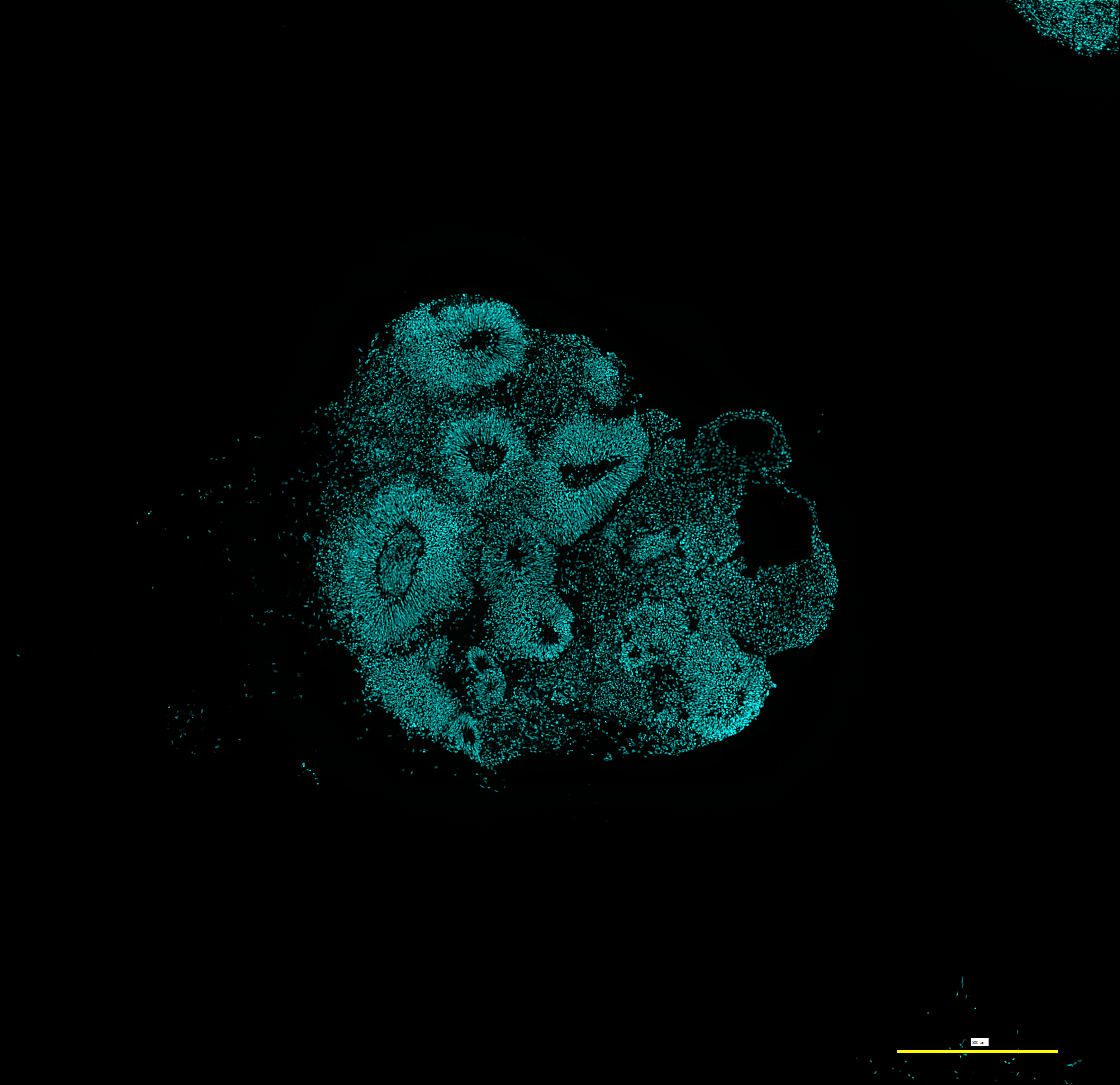

Supplement: Supplementary file 9 — Source data Fig. 2 [file 44319_2025_647_MOESM9_ESM.zip › Figure 2/2A/mutACTB-1_d30_DAPI.tif]

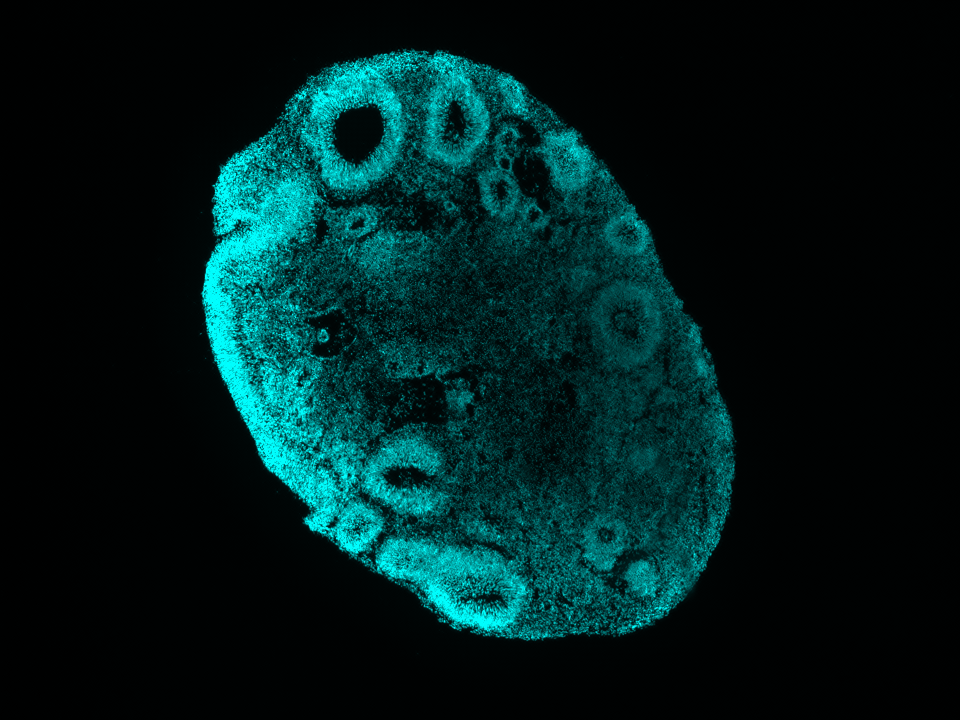

Supplement: Supplementary file 9 — Source data Fig. 2 [file 44319_2025_647_MOESM9_ESM.zip › Figure 2/2A/c1_SC102A-1_d30_DAPI.tif]

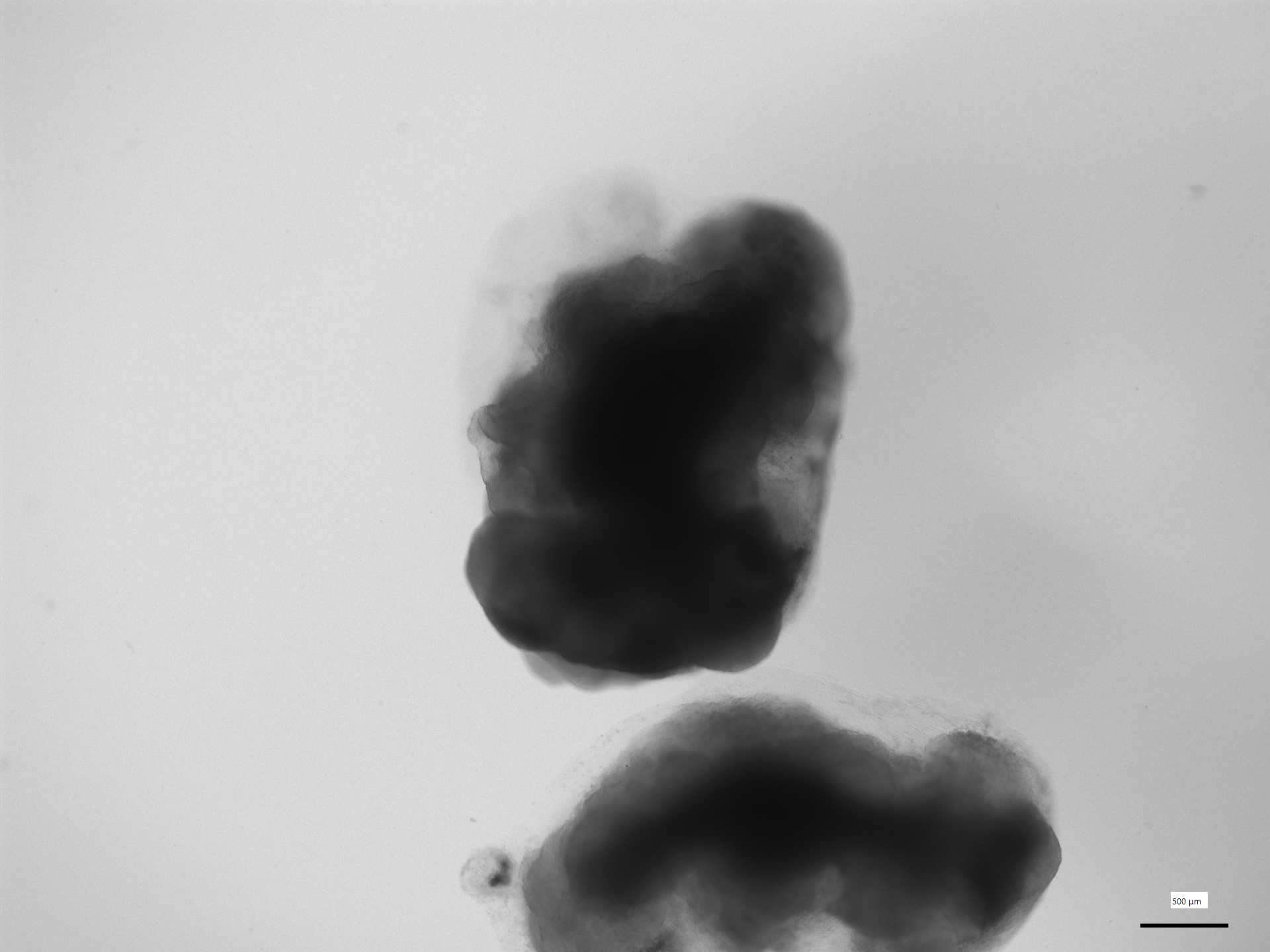

Supplement: Supplementary file 9 — Source data Fig. 2 [file 44319_2025_647_MOESM9_ESM.zip › Figure 2/2A/mutACTG1-1_d29_brightfield.tif]

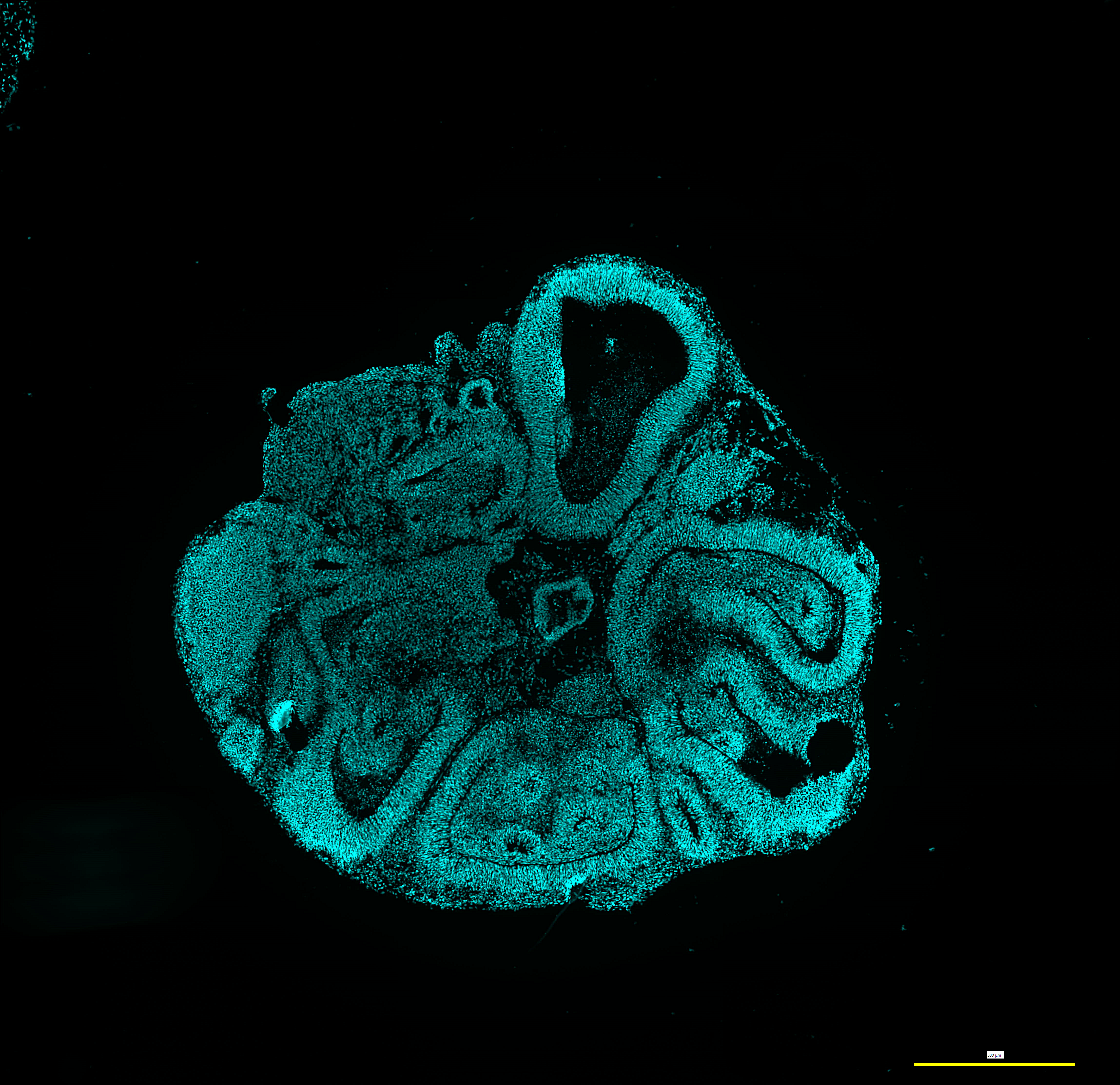

Supplement: Supplementary file 9 — Source data Fig. 2 [file 44319_2025_647_MOESM9_ESM.zip › Figure 2/2A/c2_CRTDi06-A_d30_DAPI.tif]

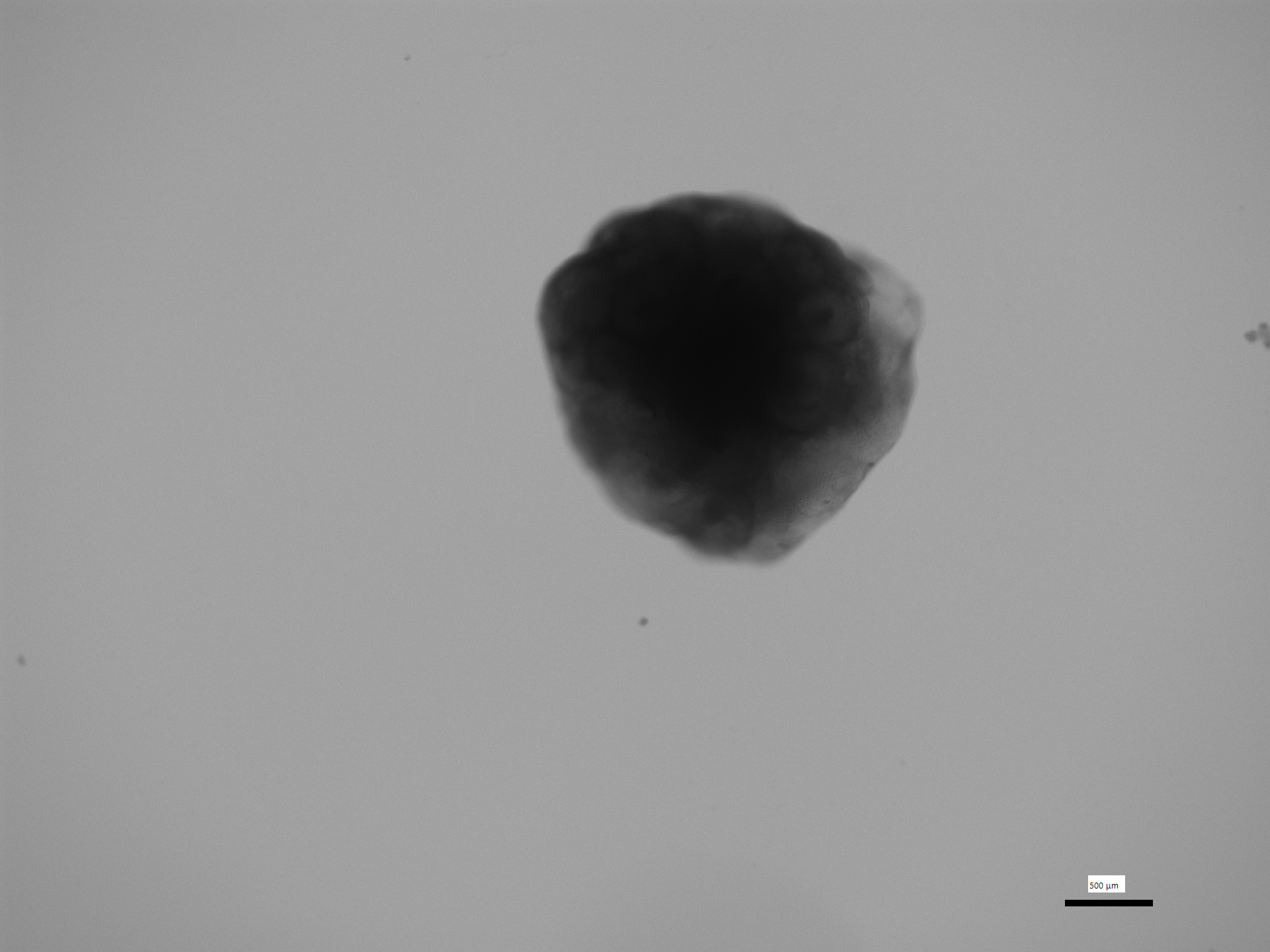

Supplement: Supplementary file 9 — Source data Fig. 2 [file 44319_2025_647_MOESM9_ESM.zip › Figure 2/2A/c2_CRTDi06-A_d29_brightfield.tif]

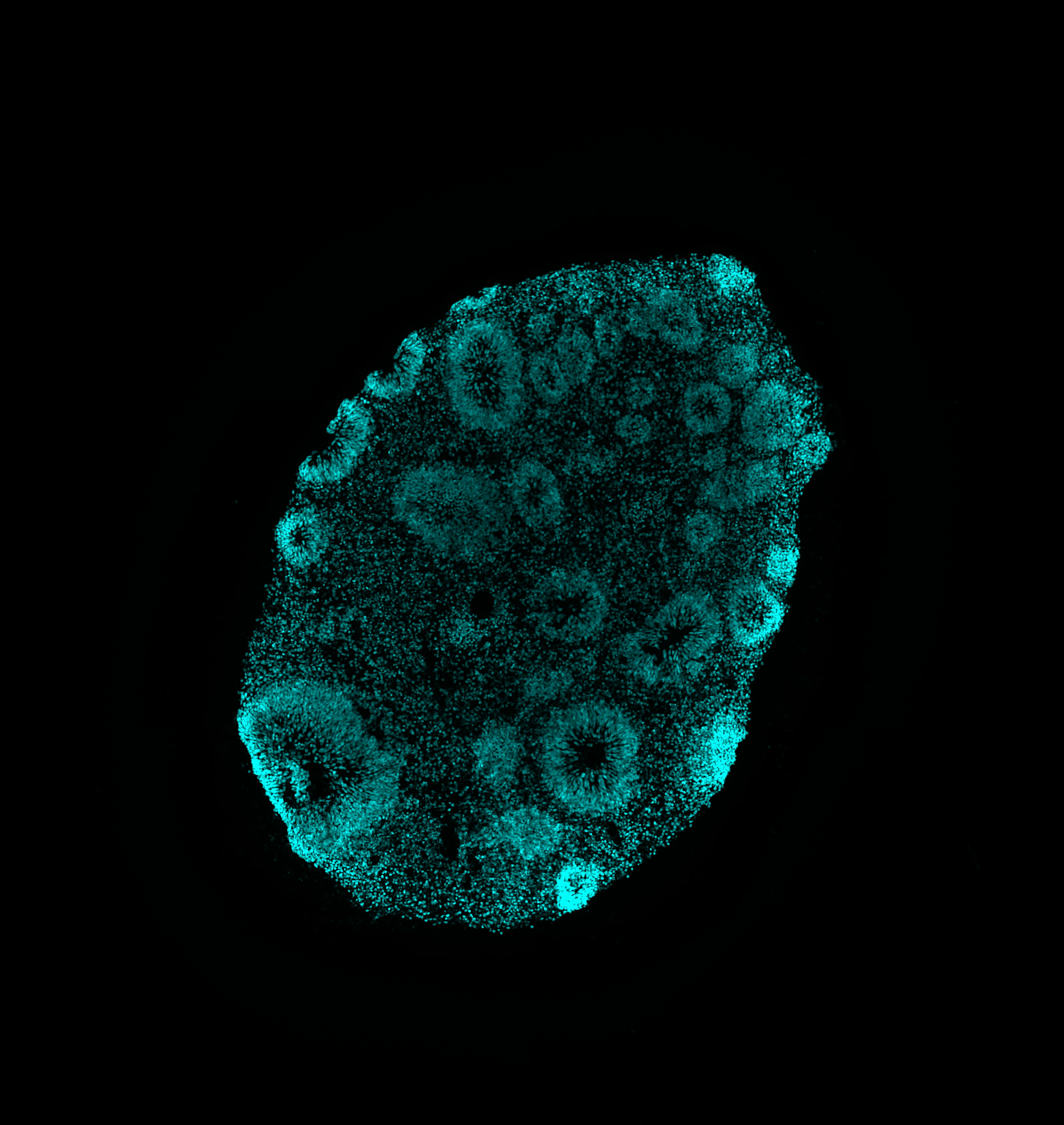

Supplement: Supplementary file 9 — Source data Fig. 2 [file 44319_2025_647_MOESM9_ESM.zip › Figure 2/2B/mutACTB-2_d50_DAPI.tif]

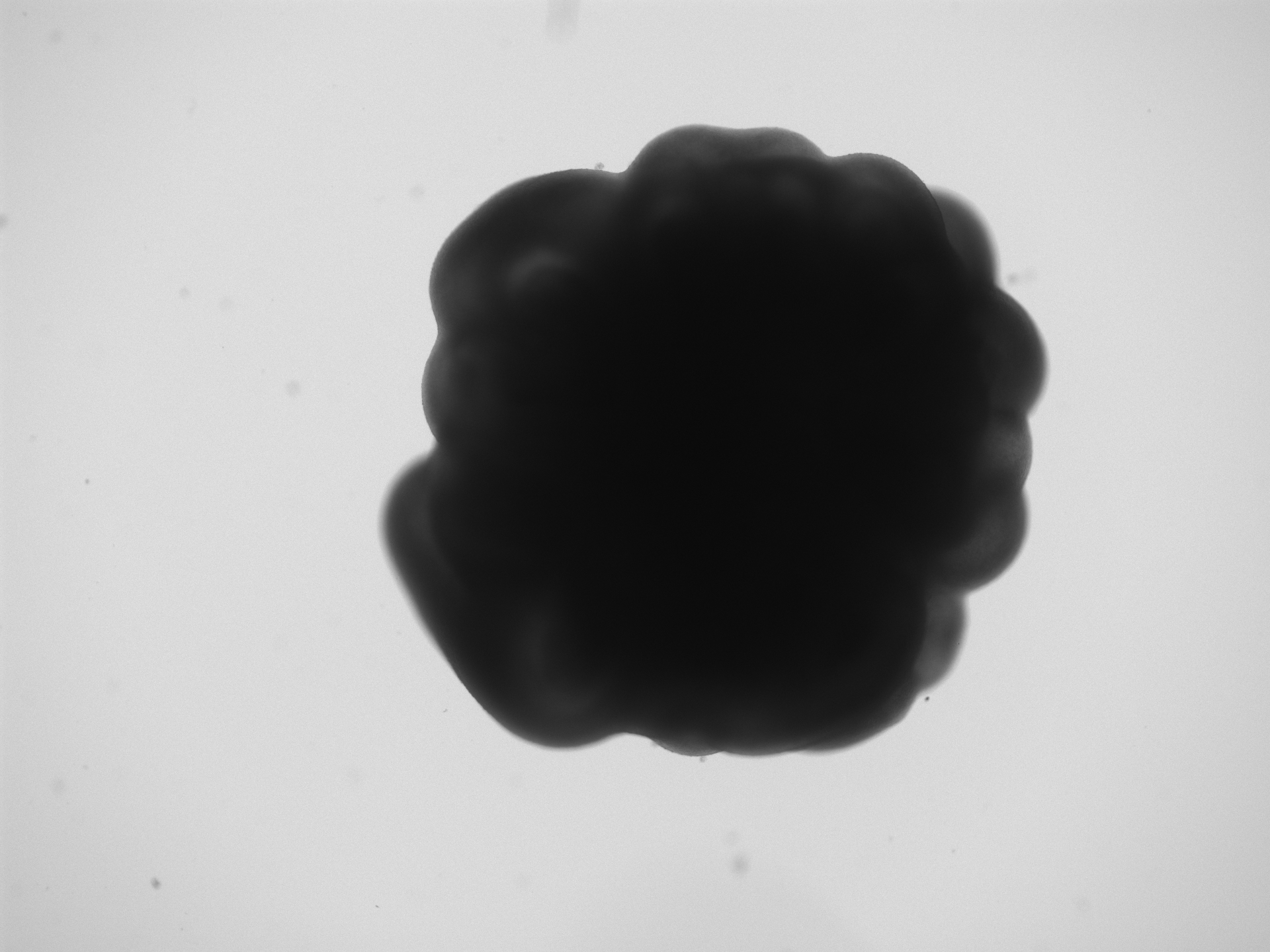

Supplement: Supplementary file 9 — Source data Fig. 2 [file 44319_2025_647_MOESM9_ESM.zip › Figure 2/2B/c2_CRTDi06-A_d50_brightfield.tif]

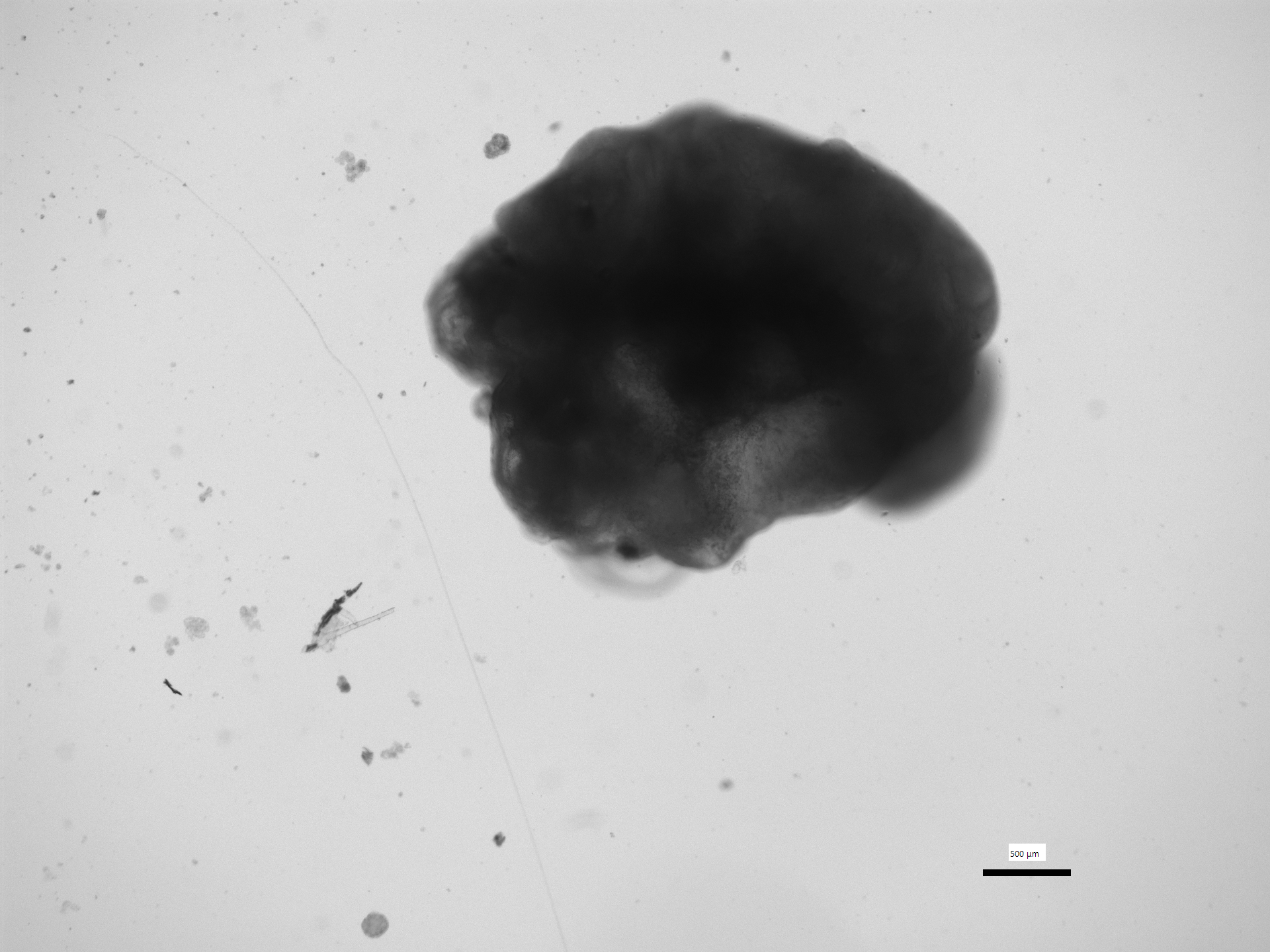

Supplement: Supplementary file 9 — Source data Fig. 2 [file 44319_2025_647_MOESM9_ESM.zip › Figure 2/2B/mutACTG1-2_d50_brightfield.tif]

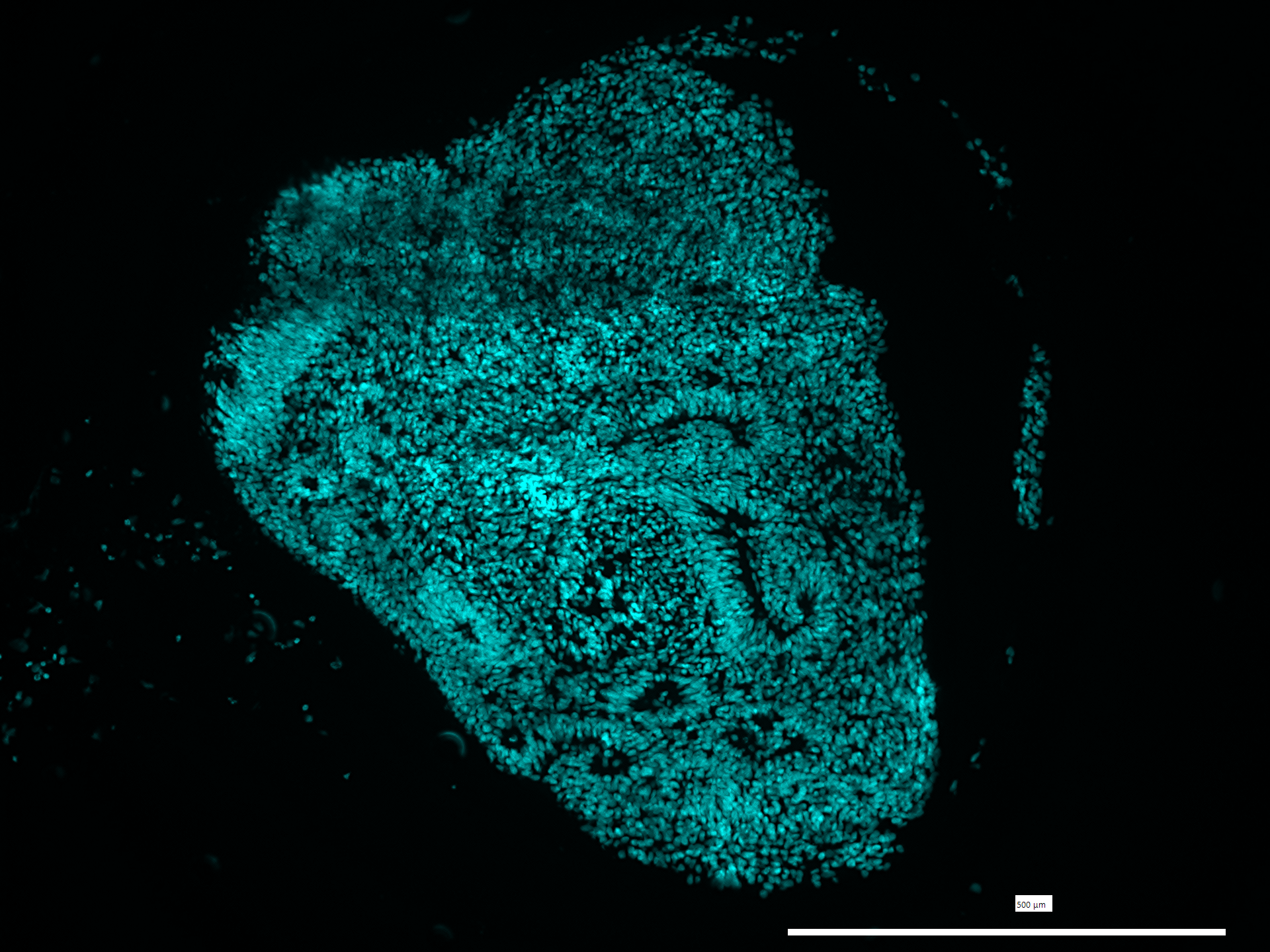

Supplement: Supplementary file 9 — Source data Fig. 2 [file 44319_2025_647_MOESM9_ESM.zip › Figure 2/2B/mutACTG1-2_d50_DAPI.tif]

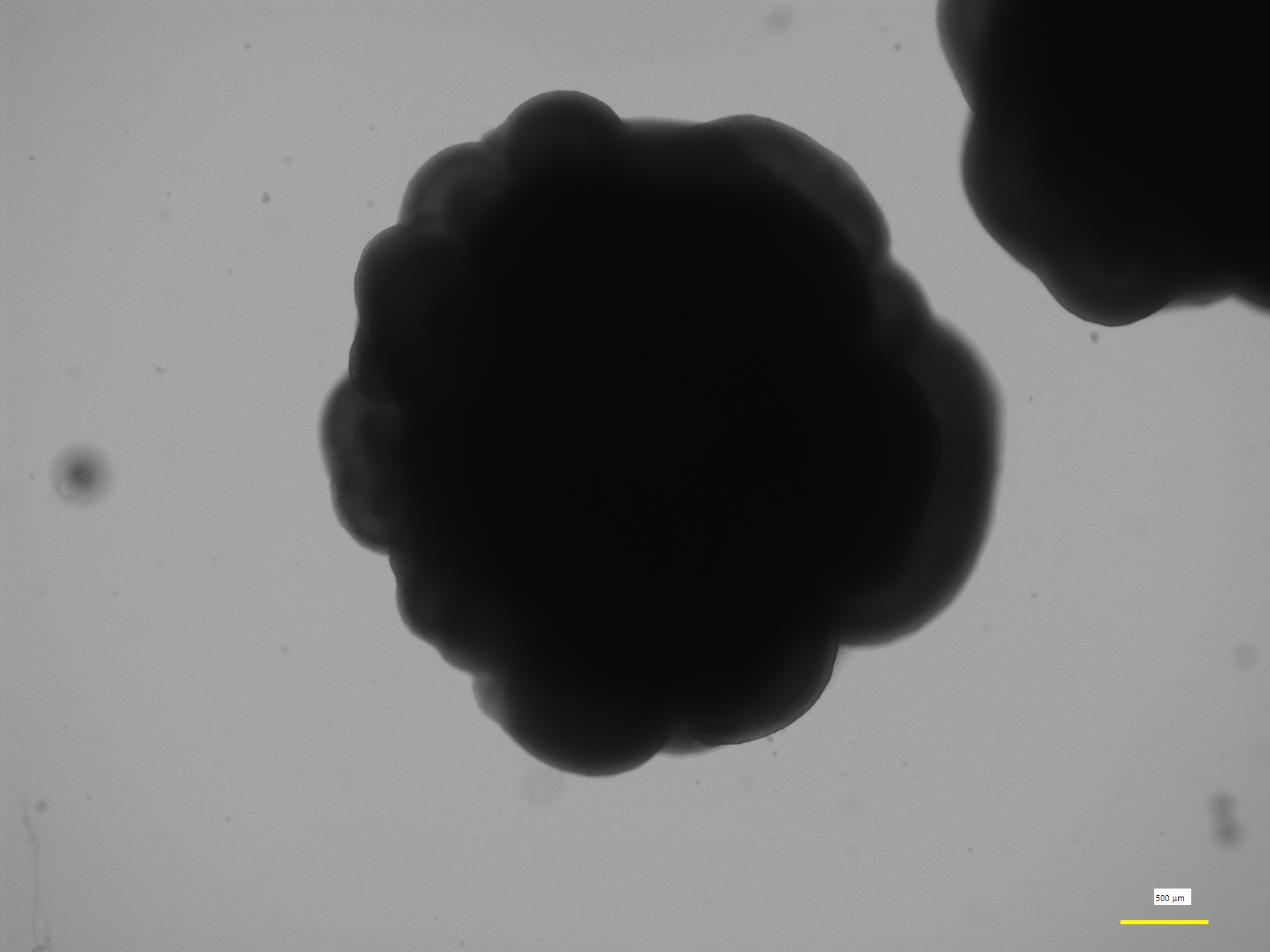

Supplement: Supplementary file 9 — Source data Fig. 2 [file 44319_2025_647_MOESM9_ESM.zip › Figure 2/2B/c1_SC102A-1_d49_brightfield.tif]

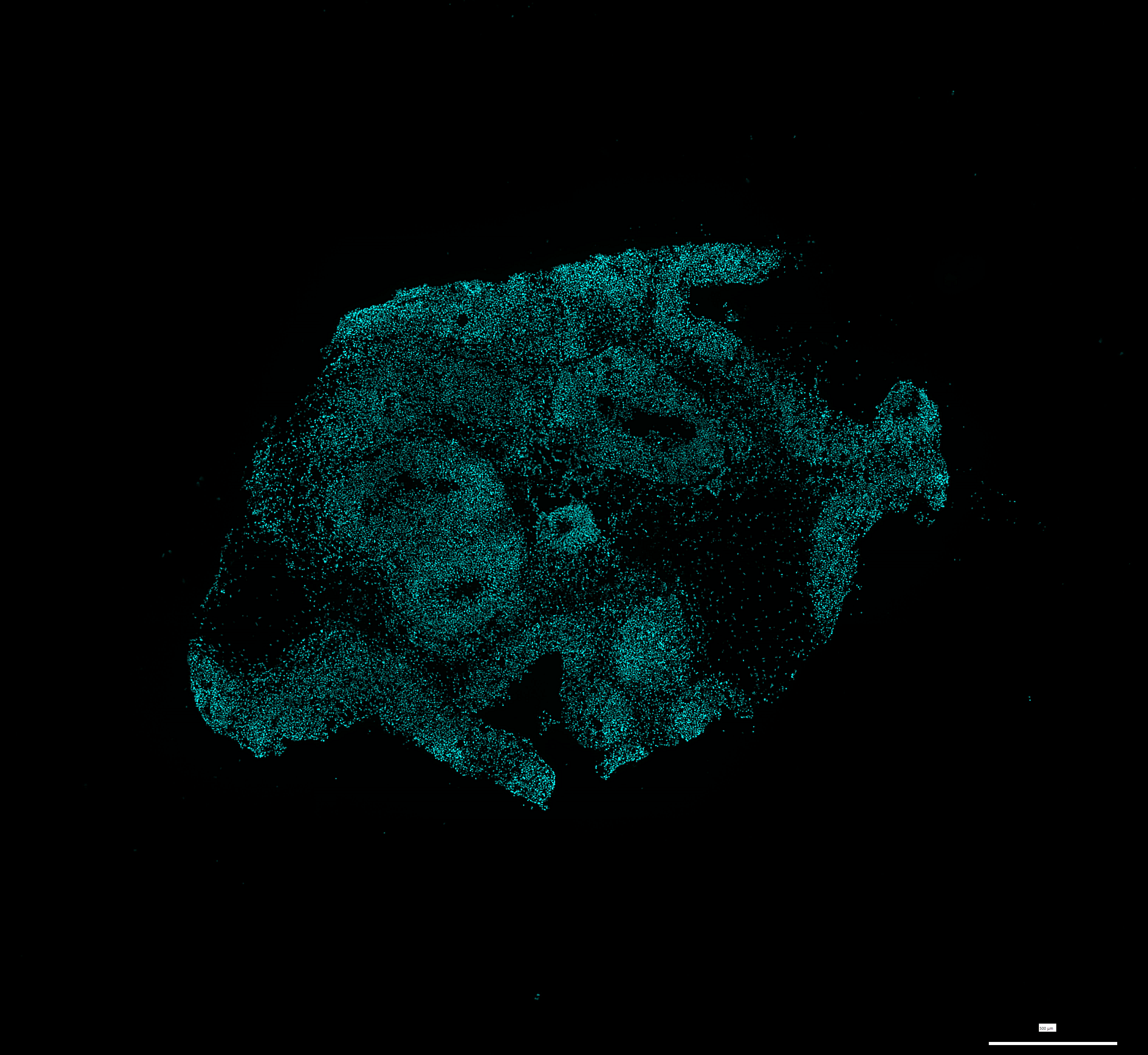

Supplement: Supplementary file 9 — Source data Fig. 2 [file 44319_2025_647_MOESM9_ESM.zip › Figure 2/2B/c2_CRTDi06-A_d50_DAPI.tif]

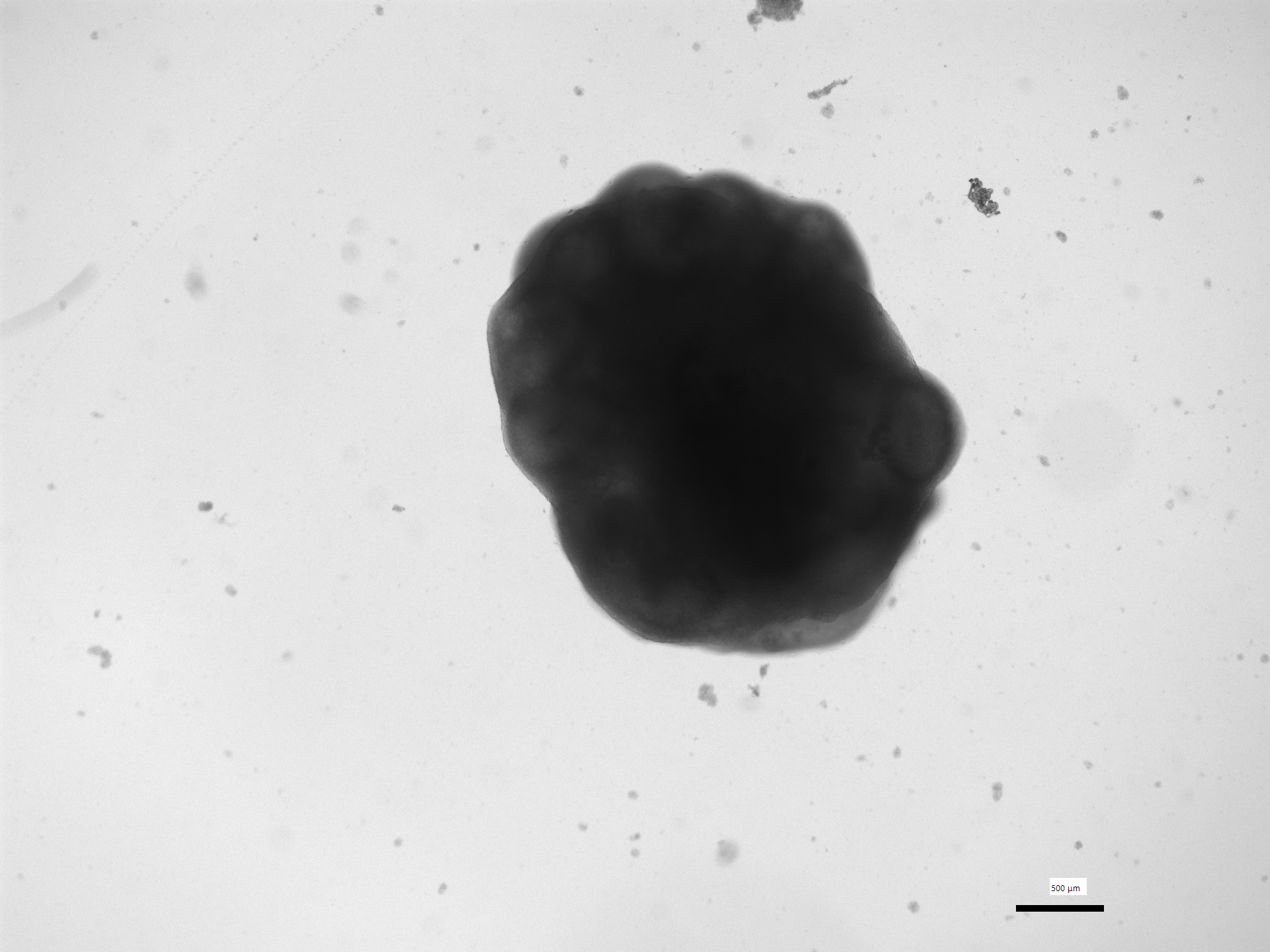

Supplement: Supplementary file 9 — Source data Fig. 2 [file 44319_2025_647_MOESM9_ESM.zip › Figure 2/2B/mutACTB-1_d50_brightfield.tif]

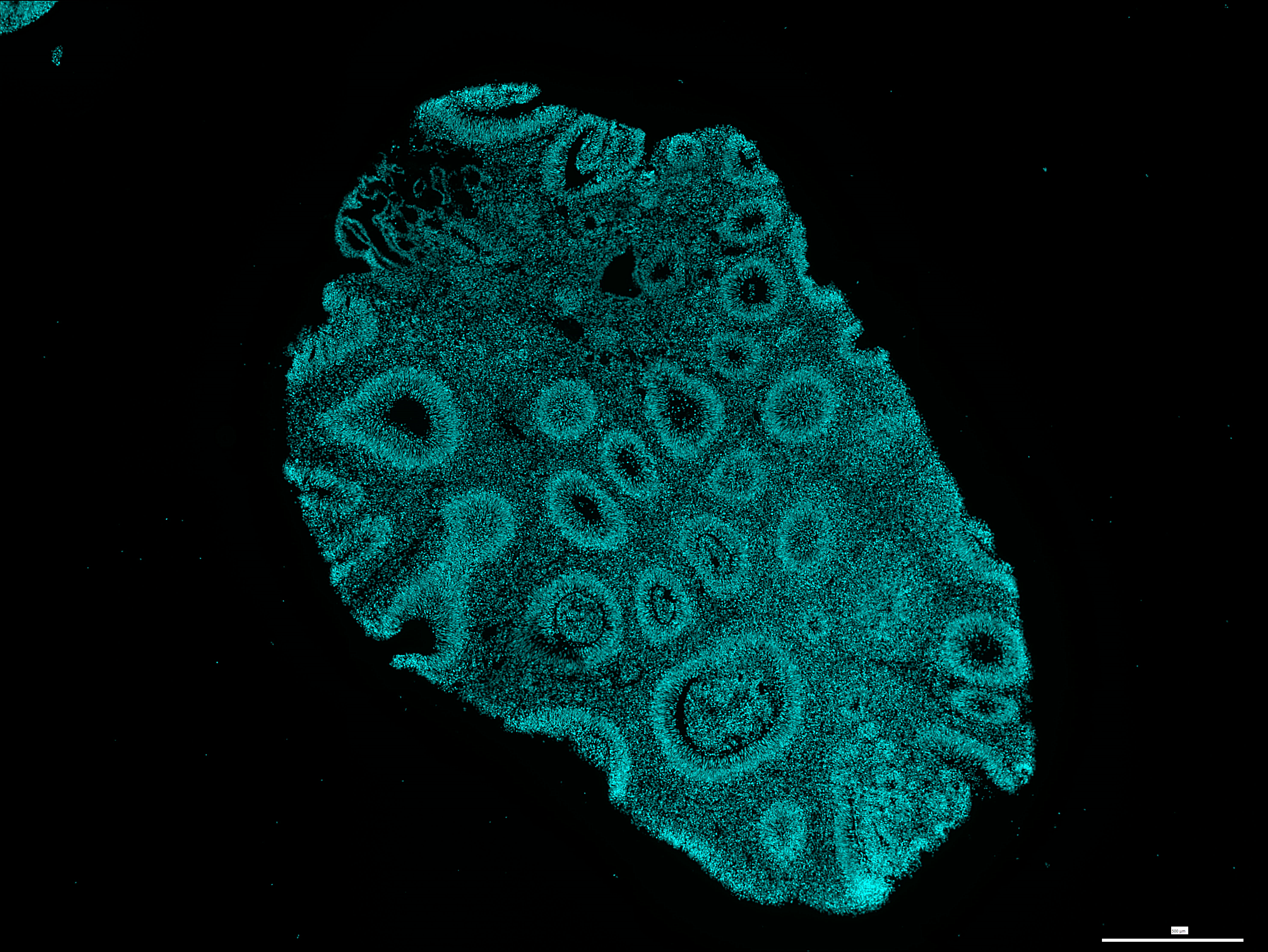

Supplement: Supplementary file 9 — Source data Fig. 2 [file 44319_2025_647_MOESM9_ESM.zip › Figure 2/2B/c1_SC102A-1_d50_DAPI.tif]

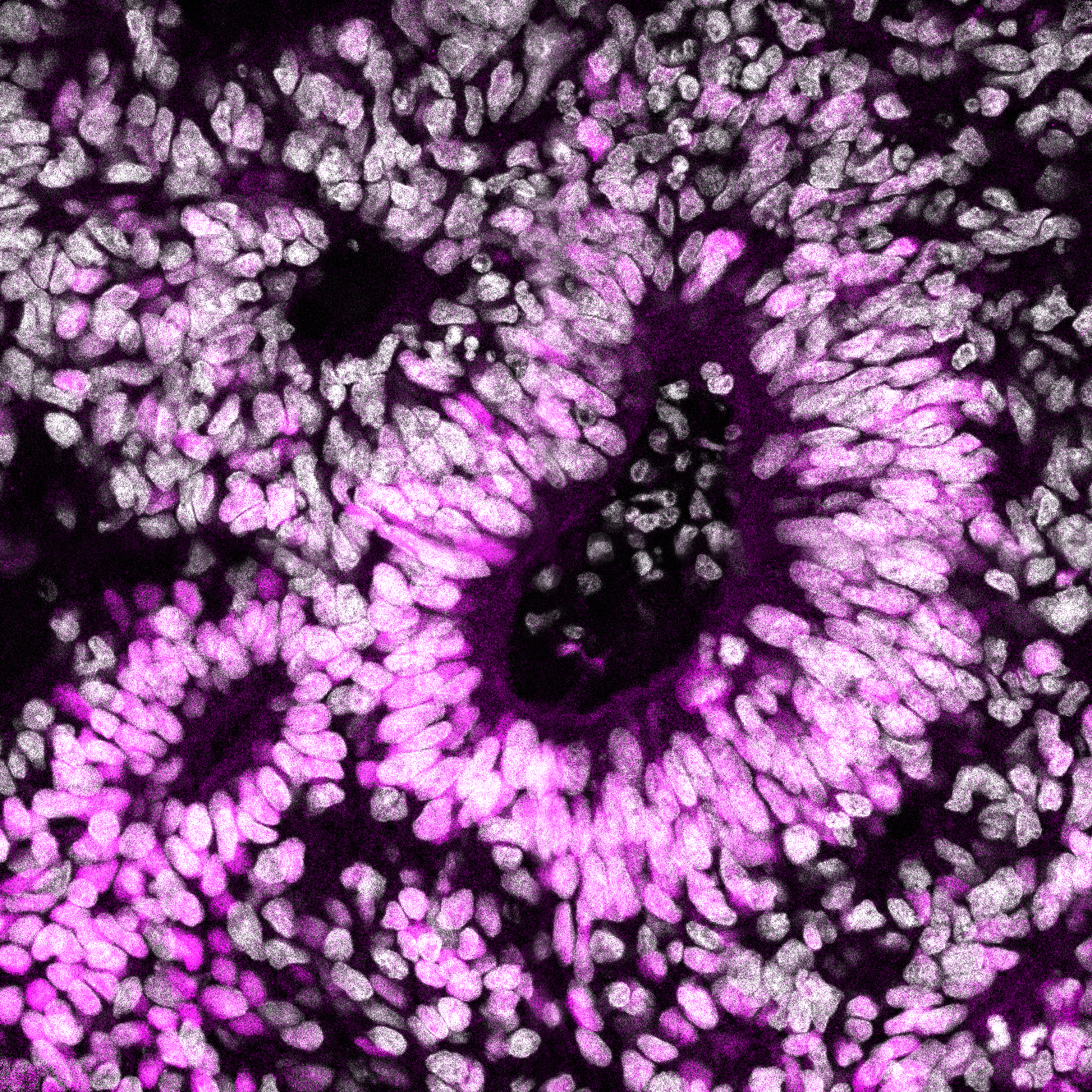

Supplement: Supplementary file 11 — Source data Fig. 4 [file 44319_2025_647_MOESM11_ESM.zip › Figure 4/4A,G/mutACTG1-1_DAPI_SOX2.tif]

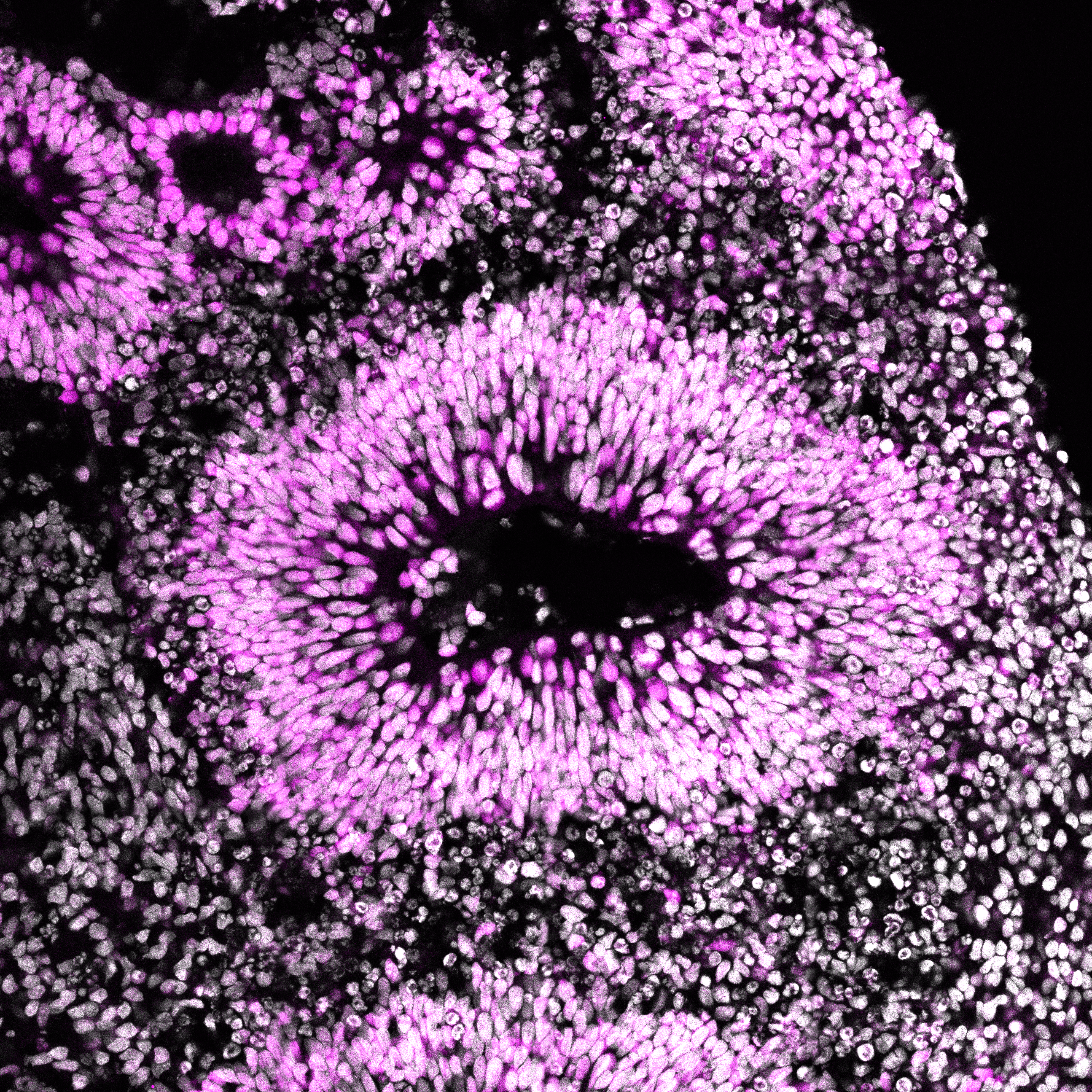

Supplement: Supplementary file 11 — Source data Fig. 4 [file 44319_2025_647_MOESM11_ESM.zip › Figure 4/4A,G/c1 SC102A-1_DAPI_SOX2.tif]

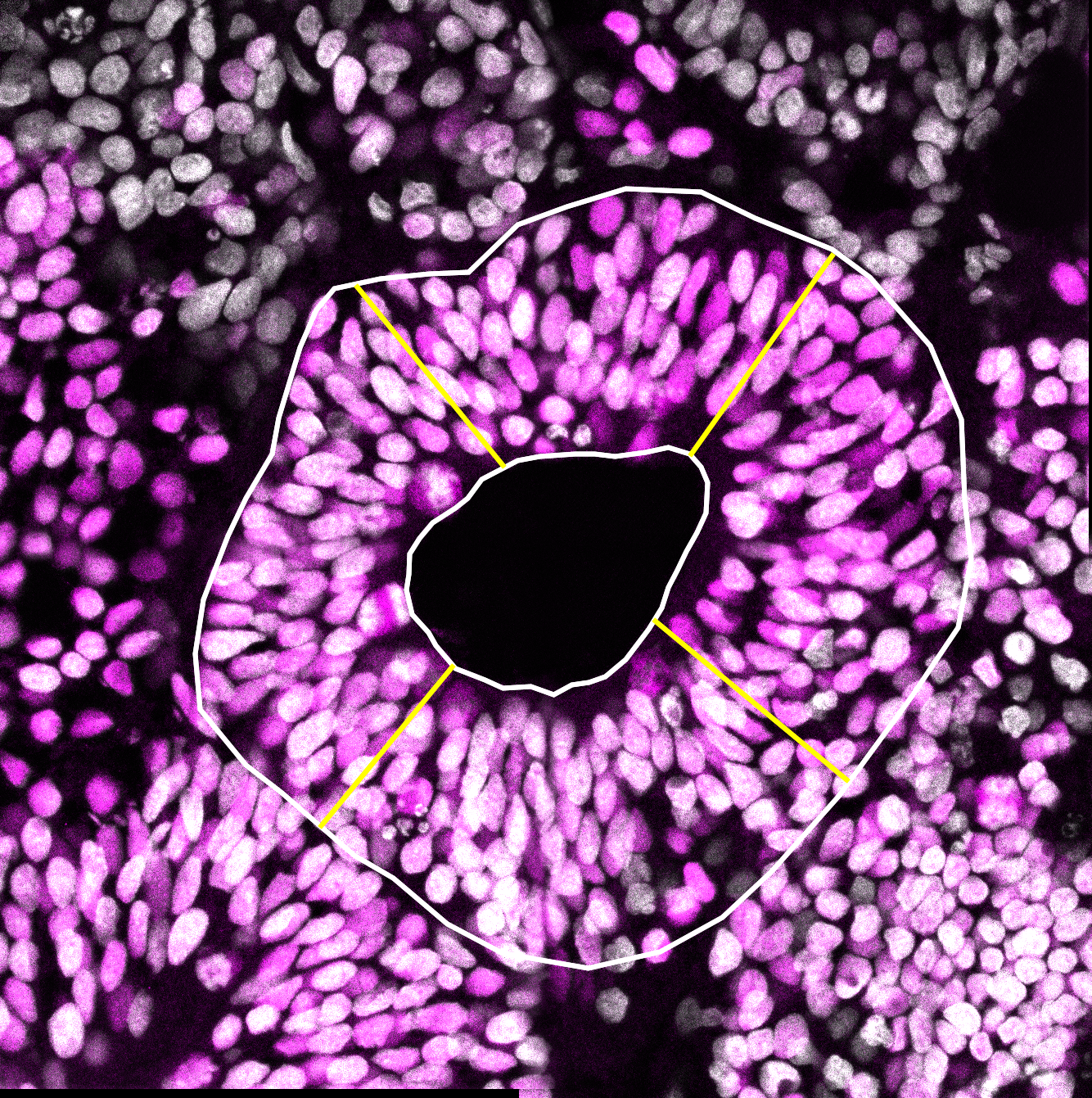

Supplement: Supplementary file 11 — Source data Fig. 4 [file 44319_2025_647_MOESM11_ESM.zip › Figure 4/4A,G/mutACTB-a_DAPI_SOX2_roi.png]

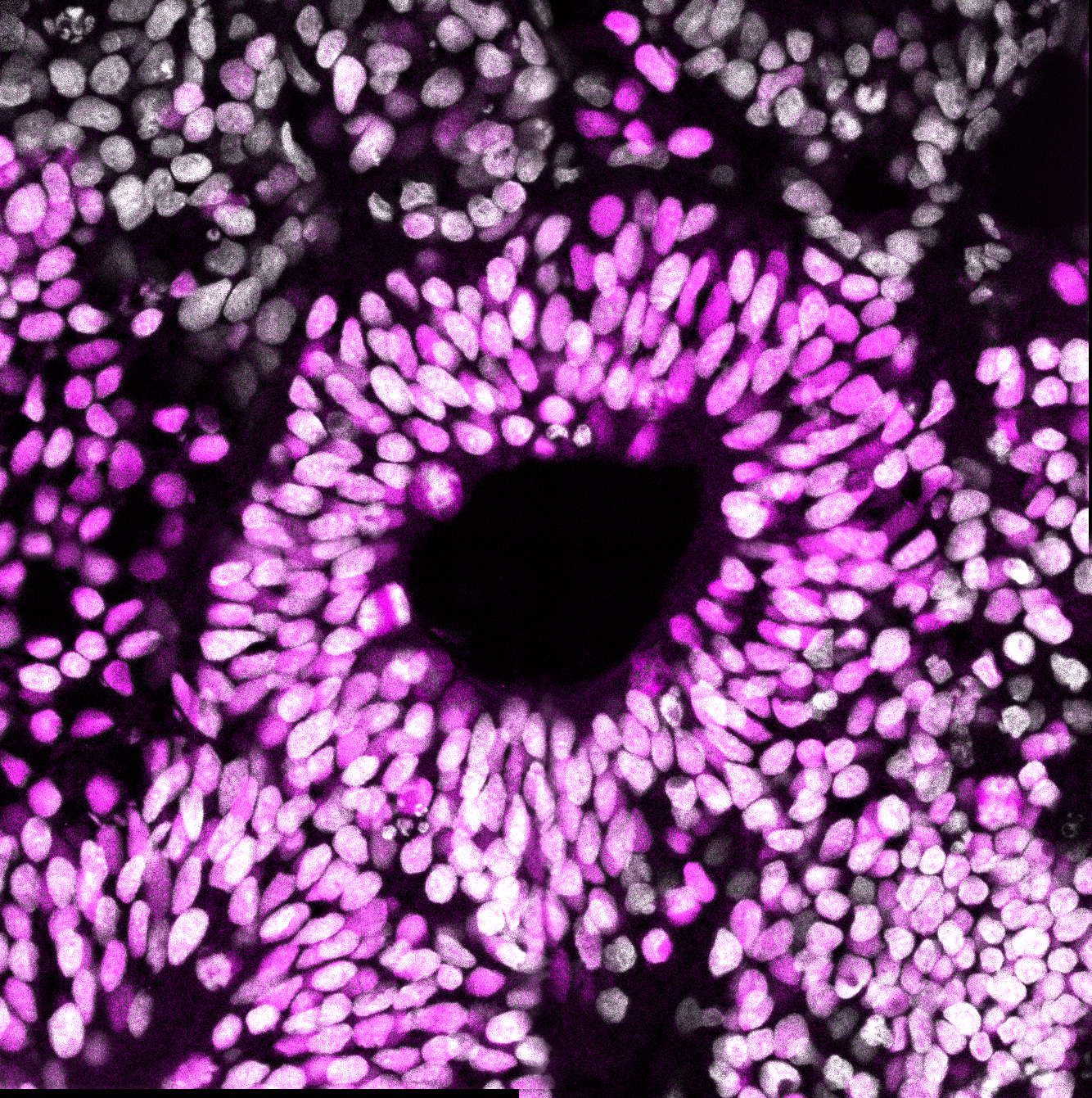

Supplement: Supplementary file 11 — Source data Fig. 4 [file 44319_2025_647_MOESM11_ESM.zip › Figure 4/4A,G/mutACTB-a_DAPI_SOX2.tif]

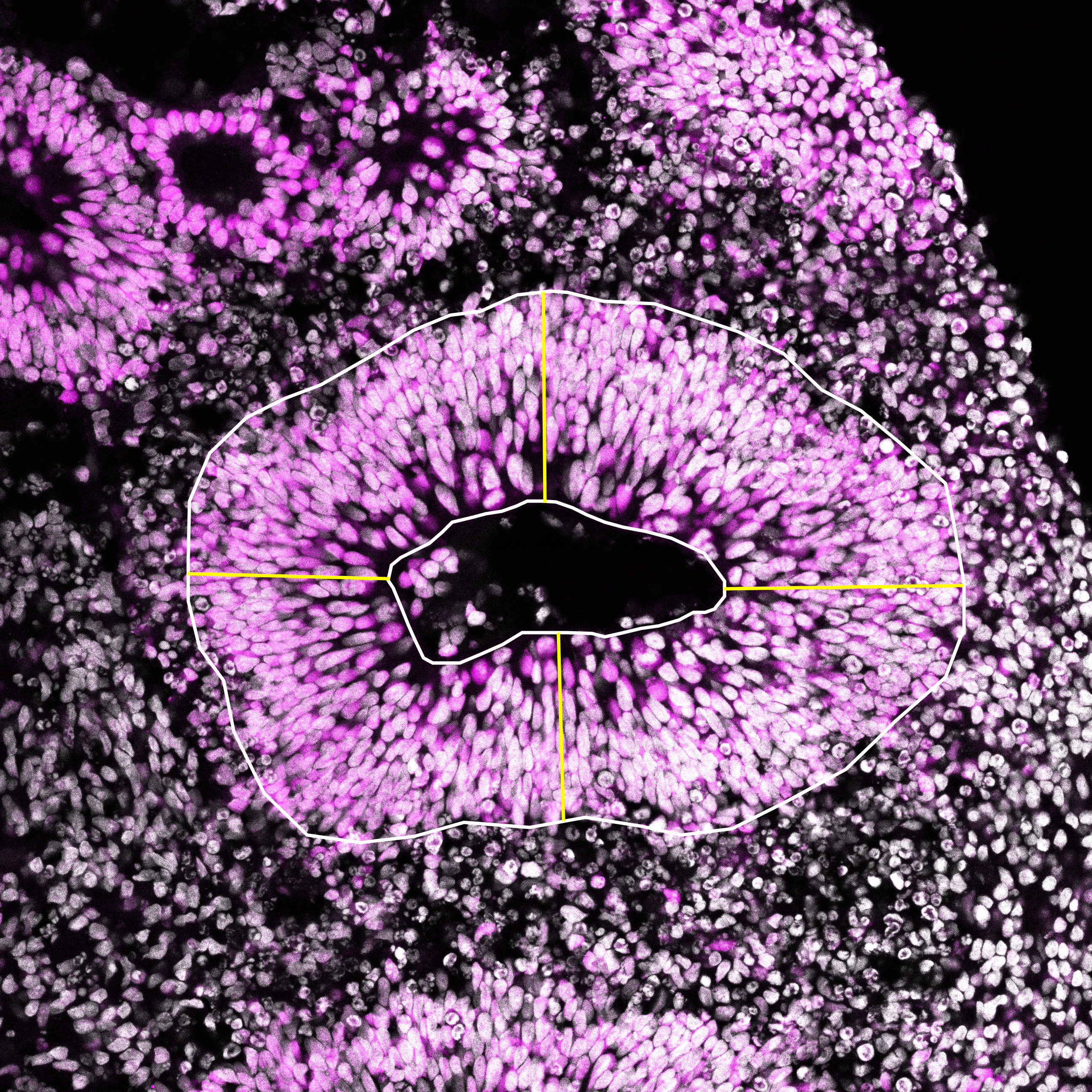

Supplement: Supplementary file 11 — Source data Fig. 4 [file 44319_2025_647_MOESM11_ESM.zip › Figure 4/4A,G/c1 SC102A-1_DAPI_SOX2_roi.png]

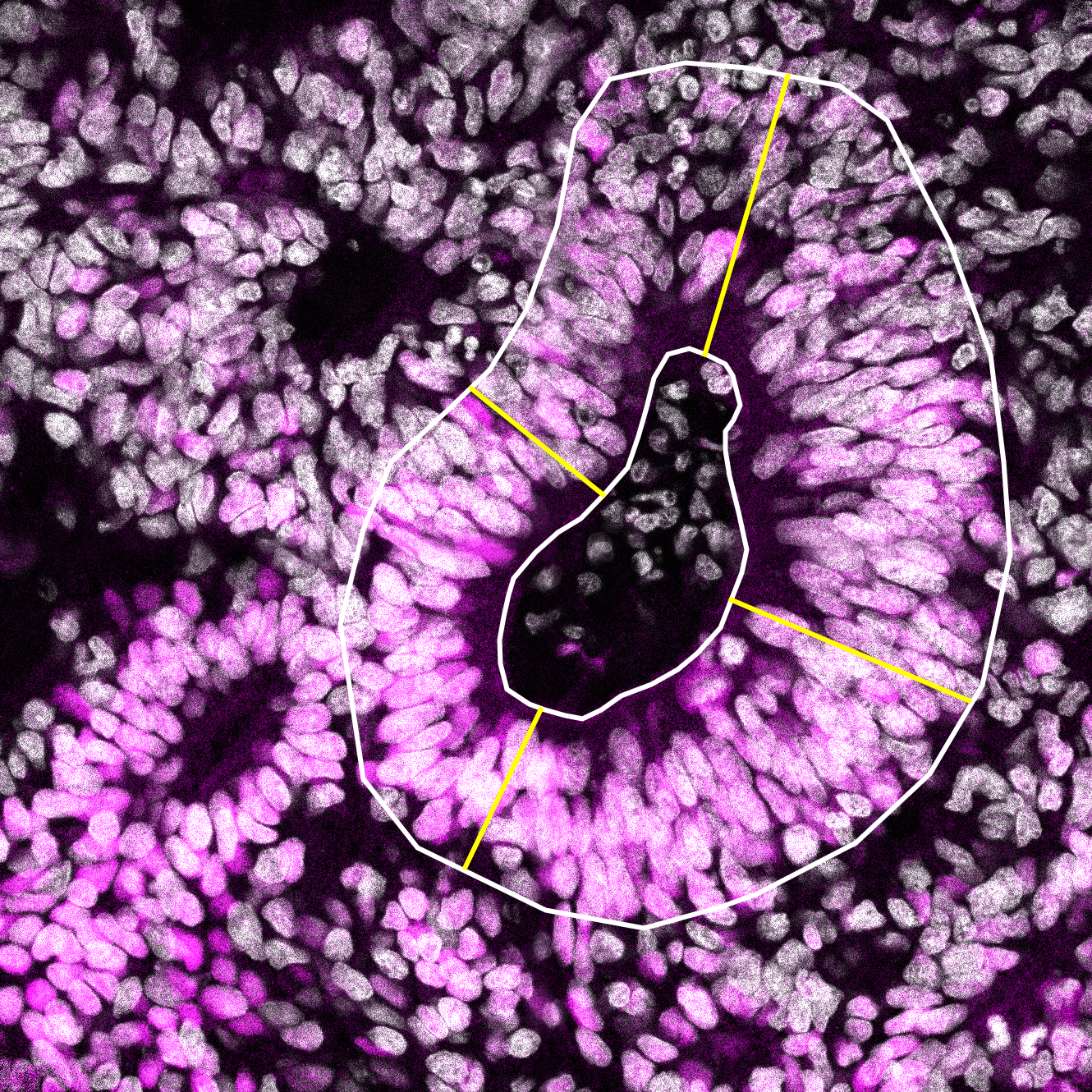

Supplement: Supplementary file 11 — Source data Fig. 4 [file 44319_2025_647_MOESM11_ESM.zip › Figure 4/4A,G/mutACTG1-1_DAPI_SOX2_roi.png]

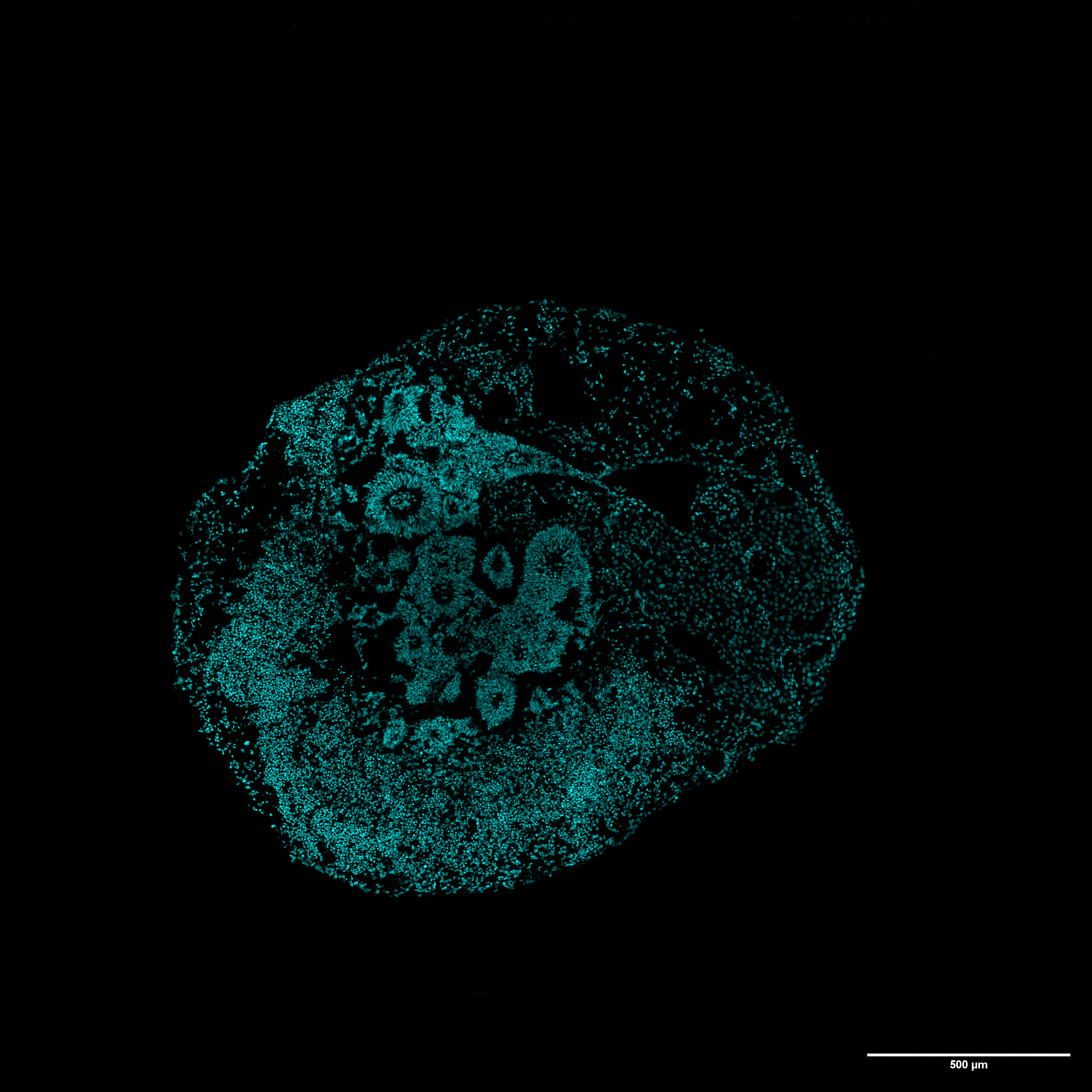

Supplement: Supplementary file 12 — Source data Fig. 5 [file 44319_2025_647_MOESM12_ESM.zip › Figure 5/5I/mutACTB-2_DAPI.png]

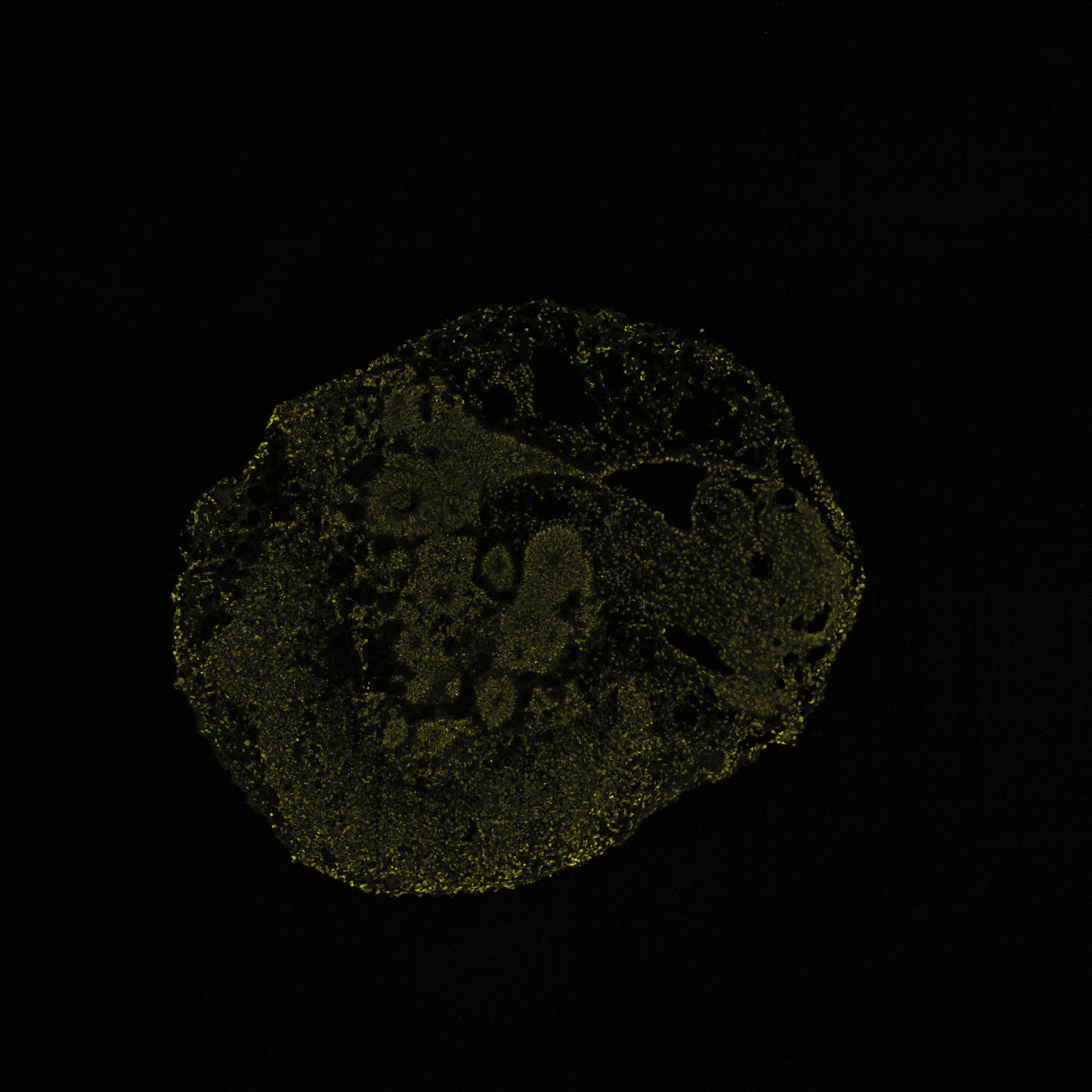

Supplement: Supplementary file 12 — Source data Fig. 5 [file 44319_2025_647_MOESM12_ESM.zip › Figure 5/5I/mutACTB-2_TBR2.tif]

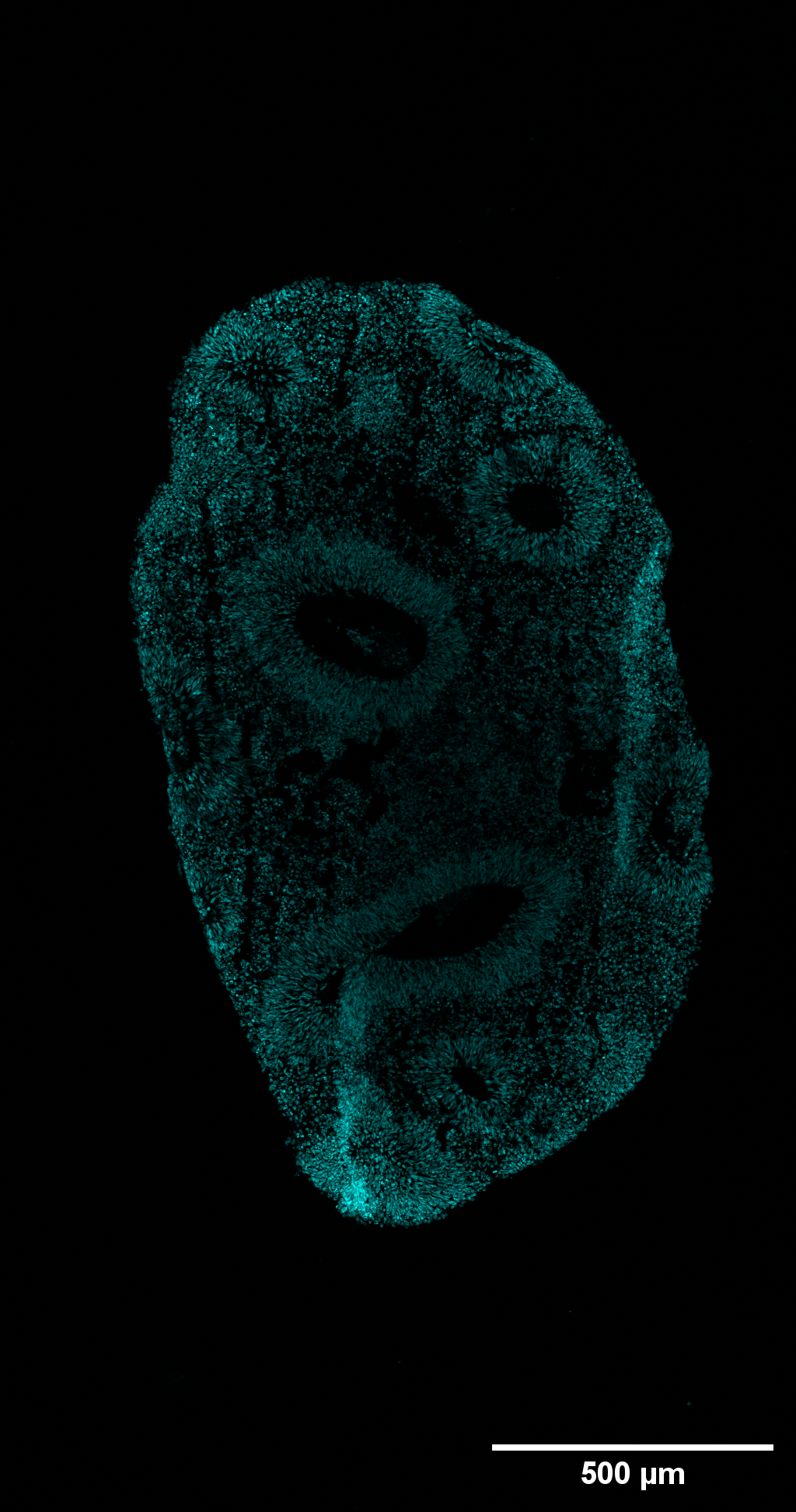

Supplement: Supplementary file 12 — Source data Fig. 5 [file 44319_2025_647_MOESM12_ESM.zip › Figure 5/5I/c1 SC102A-1_DAPI.png]

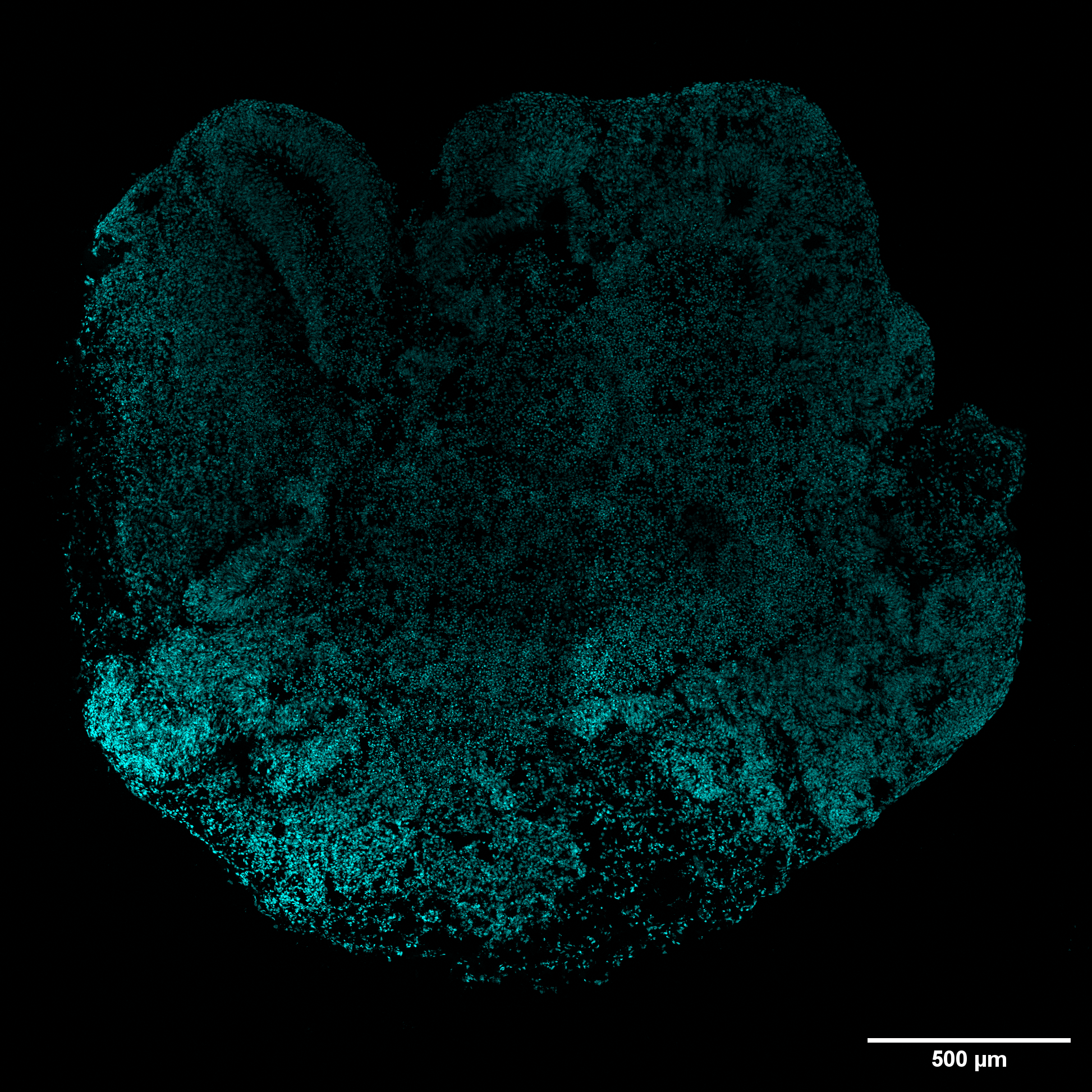

Supplement: Supplementary file 12 — Source data Fig. 5 [file 44319_2025_647_MOESM12_ESM.zip › Figure 5/5I/mutACTG1-1_DAPI.png]

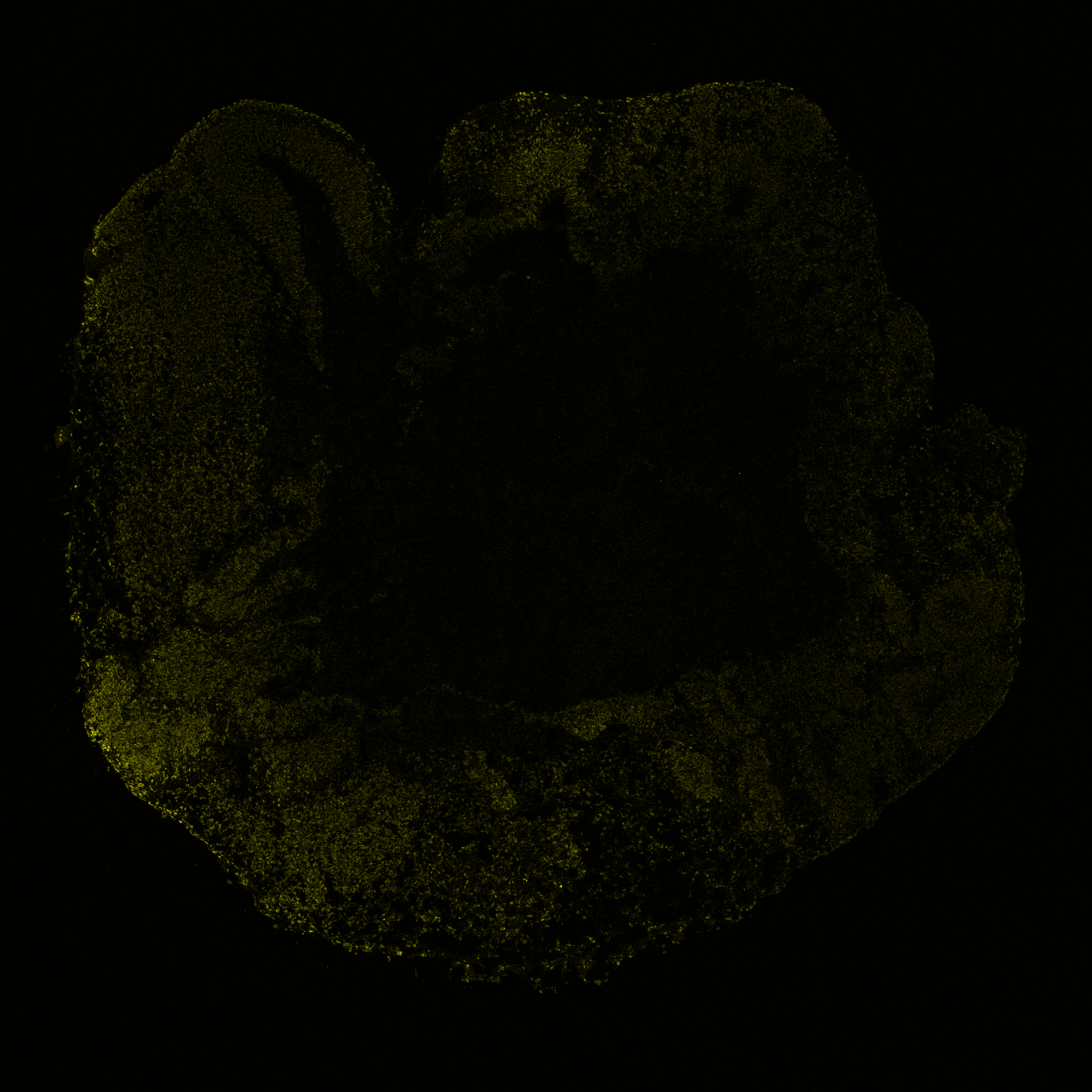

Supplement: Supplementary file 12 — Source data Fig. 5 [file 44319_2025_647_MOESM12_ESM.zip › Figure 5/5I/mutACTG1-1_TBR2.tif]

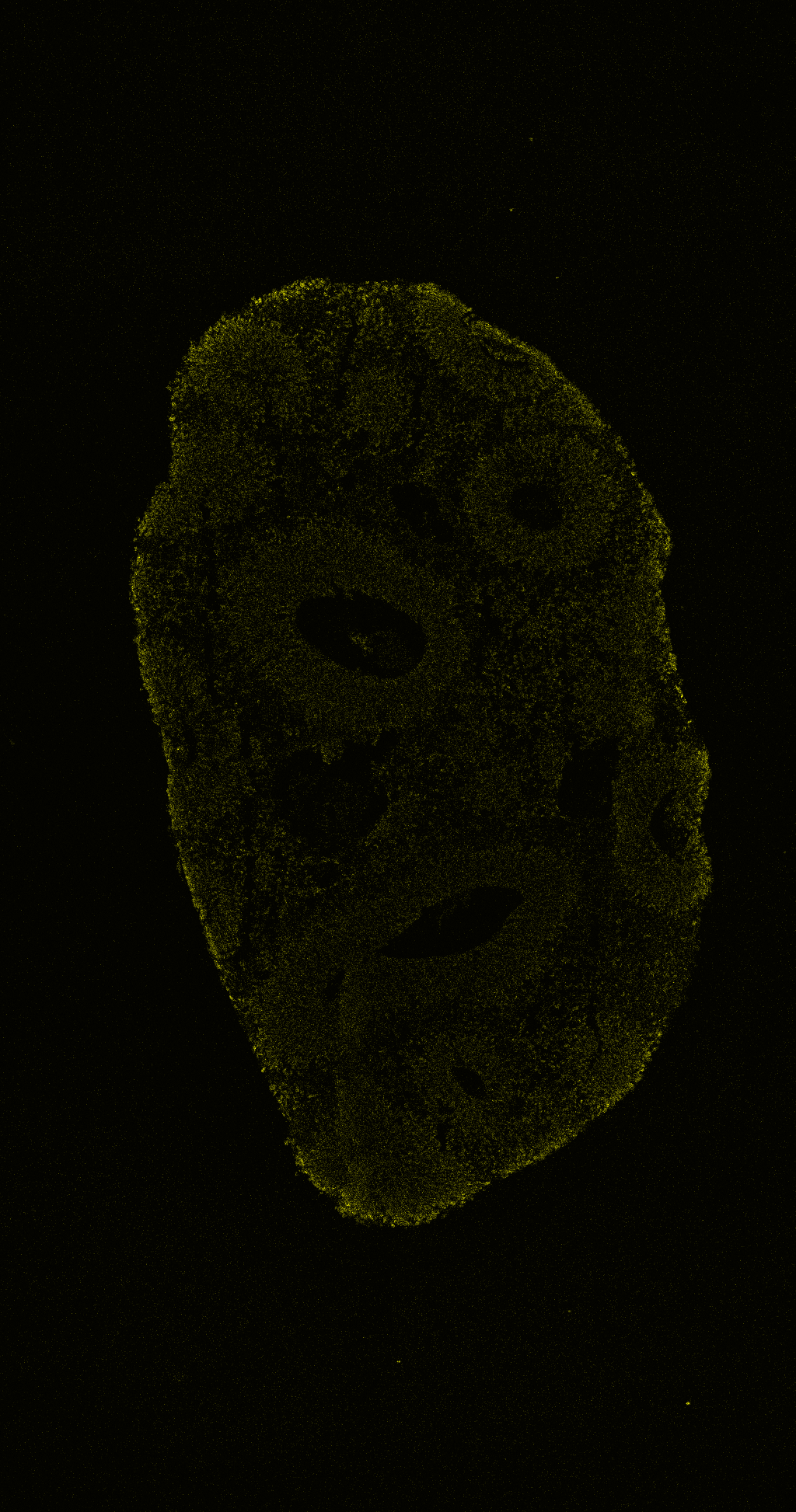

Supplement: Supplementary file 12 — Source data Fig. 5 [file 44319_2025_647_MOESM12_ESM.zip › Figure 5/5I/c1 SC102A-1_TBR2.tif]

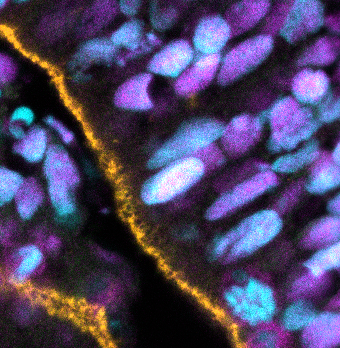

Supplement: Supplementary file 12 — Source data Fig. 5 [file 44319_2025_647_MOESM12_ESM.zip › Figure 5/5D/c2 CRTDi011-A_interphase_MERGE.tif]

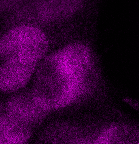

Supplement: Supplementary file 12 — Source data Fig. 5 [file 44319_2025_647_MOESM12_ESM.zip › Figure 5/5D/mutACTB-1_anaphase_horizontal cleavage_SOX2.tif]

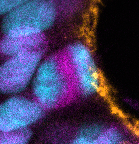

Supplement: Supplementary file 12 — Source data Fig. 5 [file 44319_2025_647_MOESM12_ESM.zip › Figure 5/5D/mutACTB-1_anaphase_horizontal cleavage_MERGE.tif]

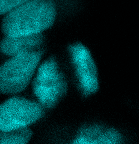

Supplement: Supplementary file 12 — Source data Fig. 5 [file 44319_2025_647_MOESM12_ESM.zip › Figure 5/5D/mutACTB-1_anaphase_horizontal cleavage_DAPI.tif]

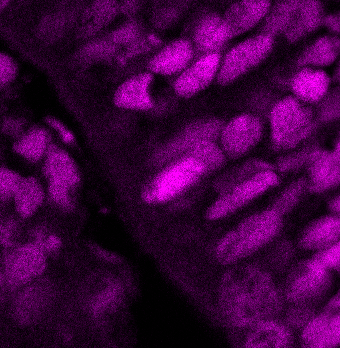

Supplement: Supplementary file 12 — Source data Fig. 5 [file 44319_2025_647_MOESM12_ESM.zip › Figure 5/5D/c2 CRTDi011-A_interphase_SOX2.tif]

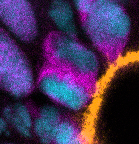

Supplement: Supplementary file 12 — Source data Fig. 5 [file 44319_2025_647_MOESM12_ESM.zip › Figure 5/5D/c2 CRTDi011-A-1_anaphase_vertical cleavage_MERGE.tif]

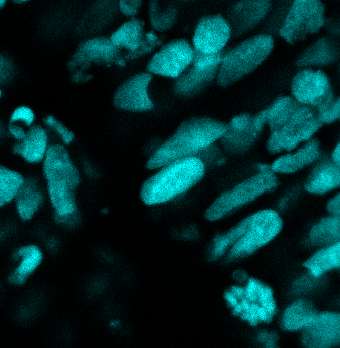

Supplement: Supplementary file 12 — Source data Fig. 5 [file 44319_2025_647_MOESM12_ESM.zip › Figure 5/5D/c2 CRTDi011-A_interphase_DAPI.tif]

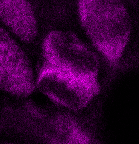

Supplement: Supplementary file 12 — Source data Fig. 5 [file 44319_2025_647_MOESM12_ESM.zip › Figure 5/5D/c2 CRTDi011-A-1_anaphase_vertical cleavage_SOX2.tif]

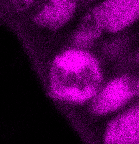

Supplement: Supplementary file 12 — Source data Fig. 5 [file 44319_2025_647_MOESM12_ESM.zip › Figure 5/5D/c2 CRTDi011-A_metaphase_SOX2.tif]

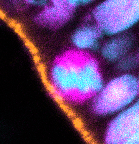

Supplement: Supplementary file 12 — Source data Fig. 5 [file 44319_2025_647_MOESM12_ESM.zip › Figure 5/5D/c2 CRTDi011-A_metaphase_MERGE.tif]

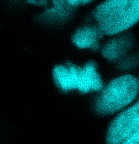

Supplement: Supplementary file 12 — Source data Fig. 5 [file 44319_2025_647_MOESM12_ESM.zip › Figure 5/5D/c2 CRTDi011-A_metaphase_DAPI.tif]

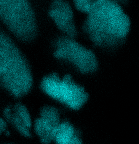

Supplement: Supplementary file 12 — Source data Fig. 5 [file 44319_2025_647_MOESM12_ESM.zip › Figure 5/5D/c2 CRTDi011-A-1_anaphase_vertical cleavage_DAPI.tif]

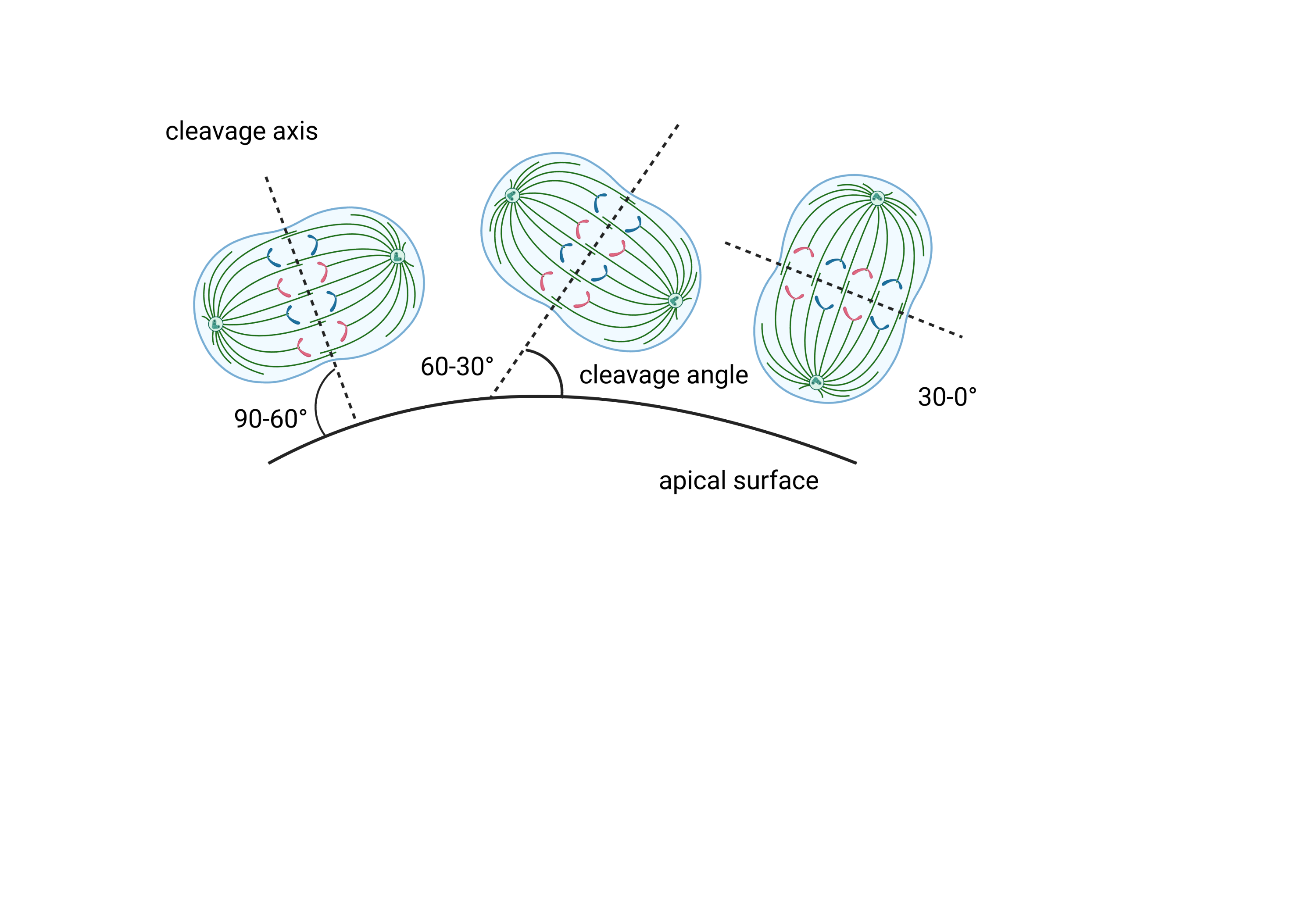

Supplement: Supplementary file 12 — Source data Fig. 5 [file 44319_2025_647_MOESM12_ESM.zip › Figure 5/5E/Cell Cleavage_20220305.png]

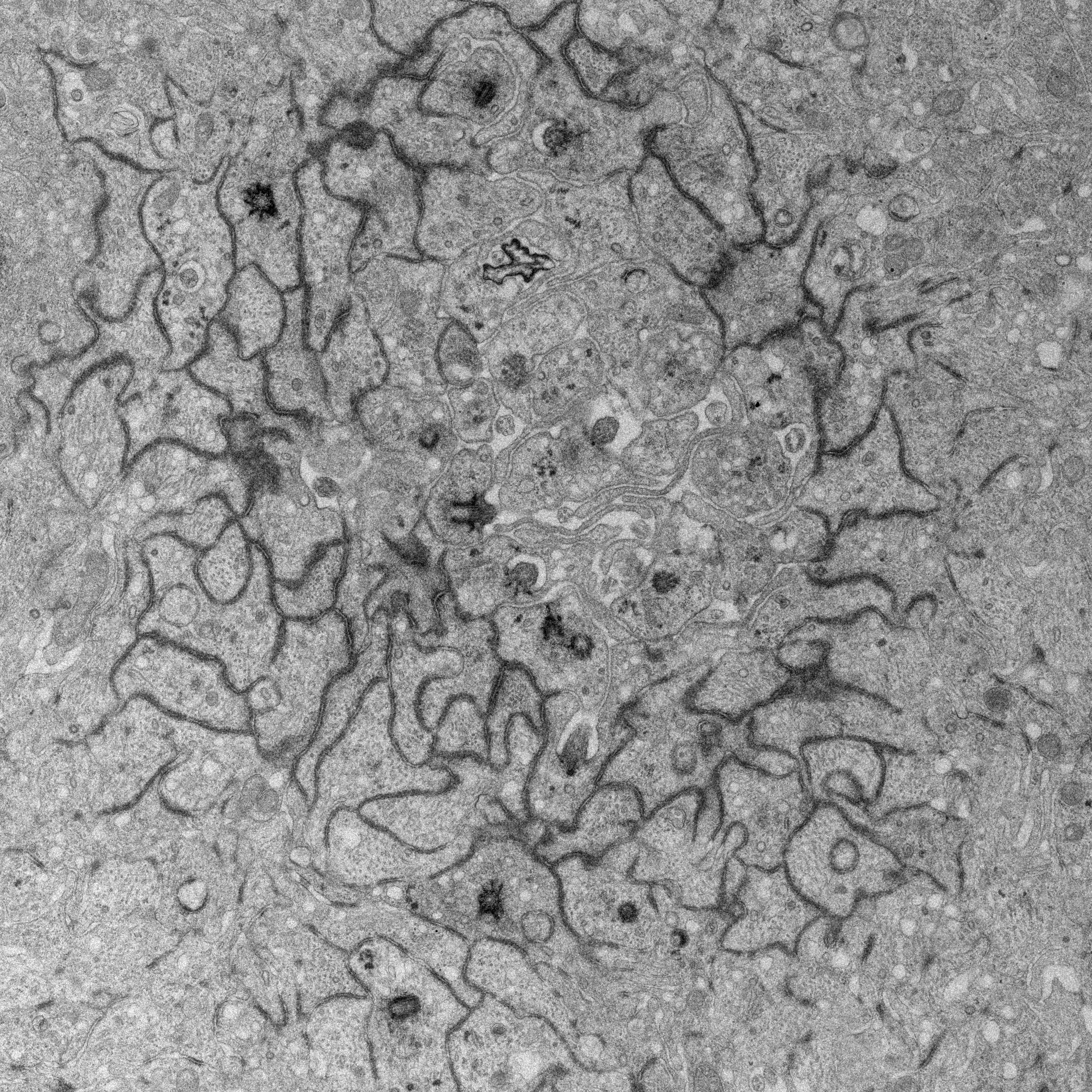

Supplement: Supplementary file 13 — Source data Fig. 6 A, B [file 44319_2025_647_MOESM13_ESM.zip › Figure 6A_B/6A/mutACTB-1.tif]

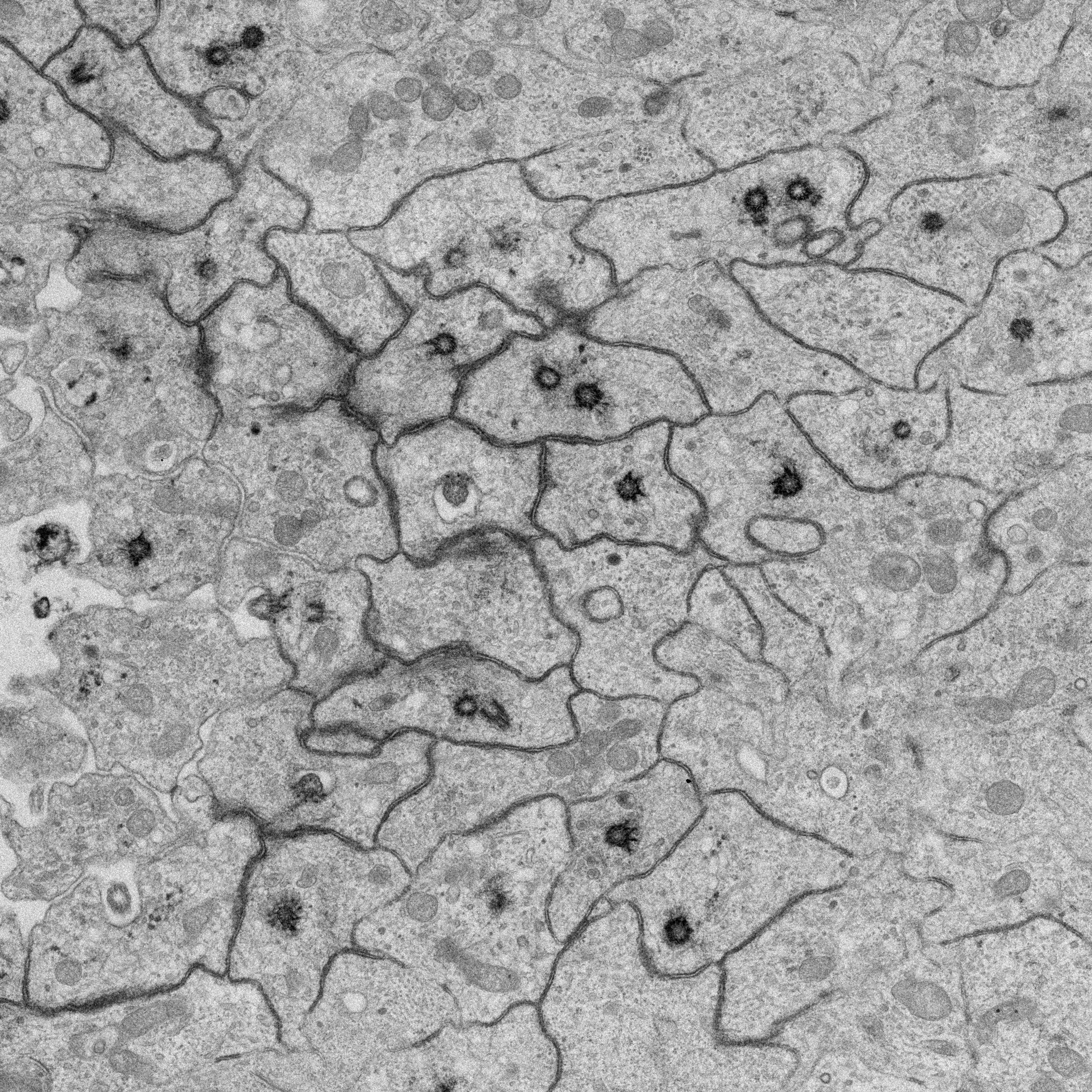

Supplement: Supplementary file 13 — Source data Fig. 6 A, B [file 44319_2025_647_MOESM13_ESM.zip › Figure 6A_B/6A/c2 CRTDi011-A.tif]

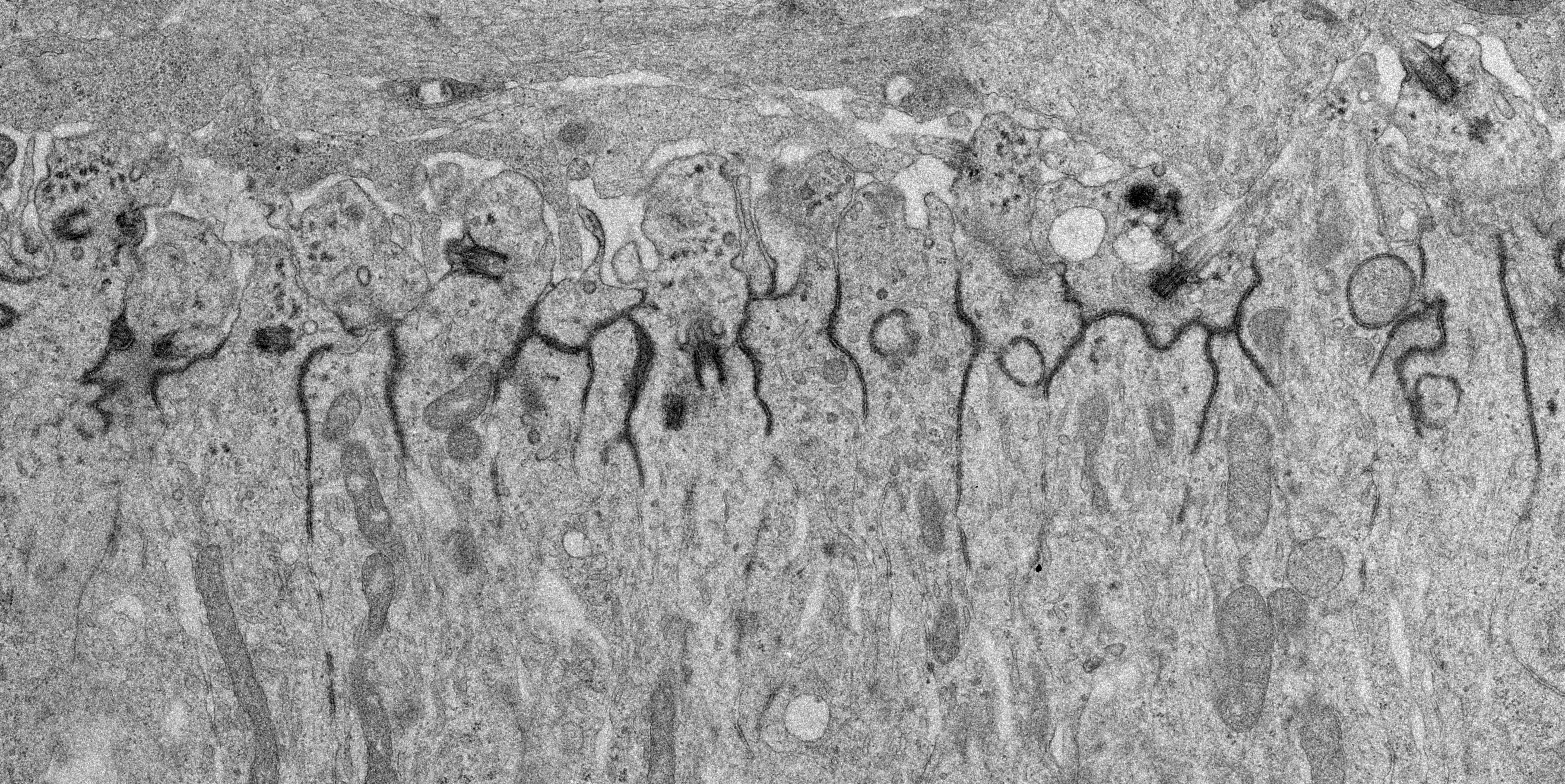

Supplement: Supplementary file 13 — Source data Fig. 6 A, B [file 44319_2025_647_MOESM13_ESM.zip › Figure 6A_B/6B/mutACTG1-2.tif]

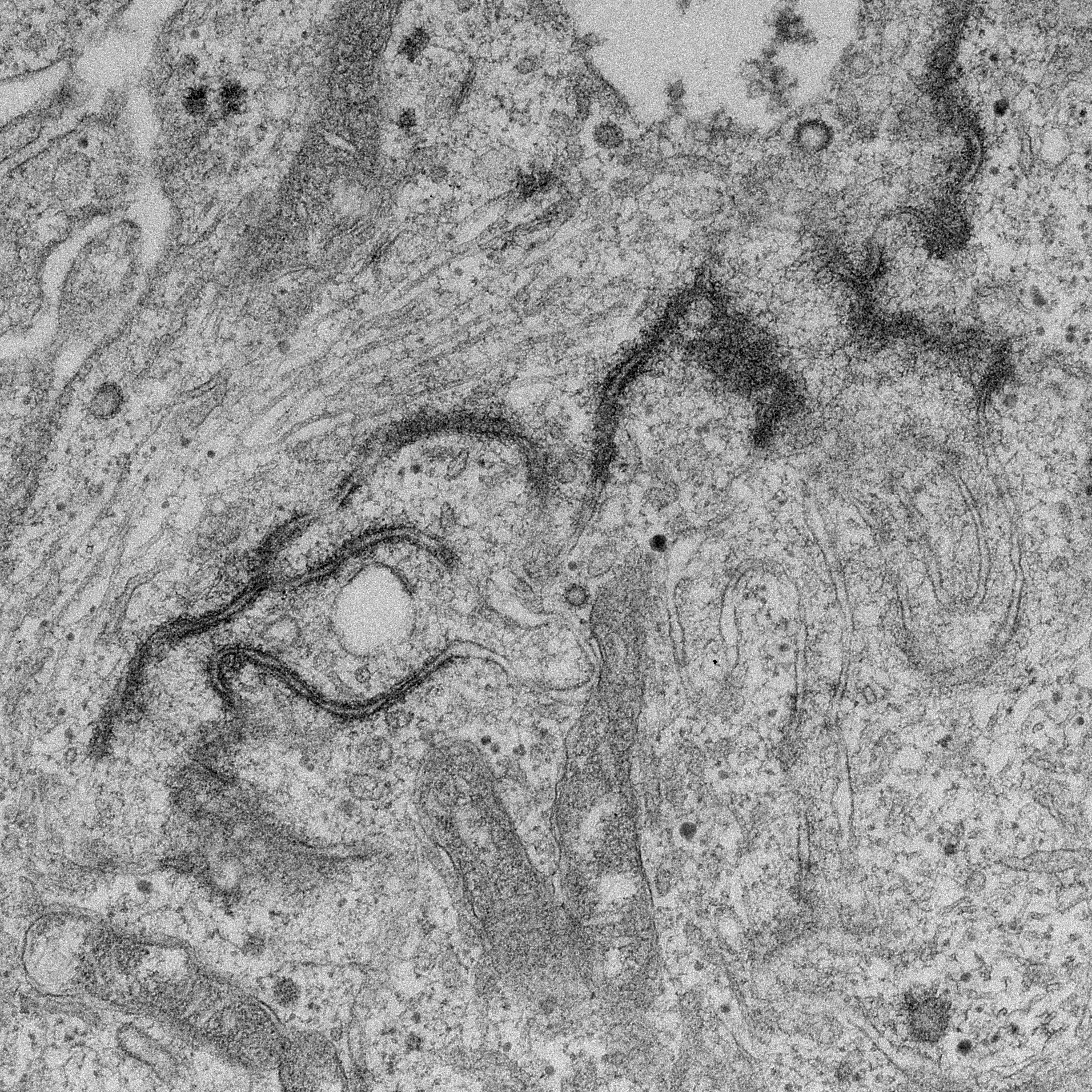

Supplement: Supplementary file 16 — Source data Fig. 6 replicate 2 [file 44319_2025_647_MOESM16_ESM.zip › Figure 6_replicate 2/mutACTB_organoid-2-1.tif]

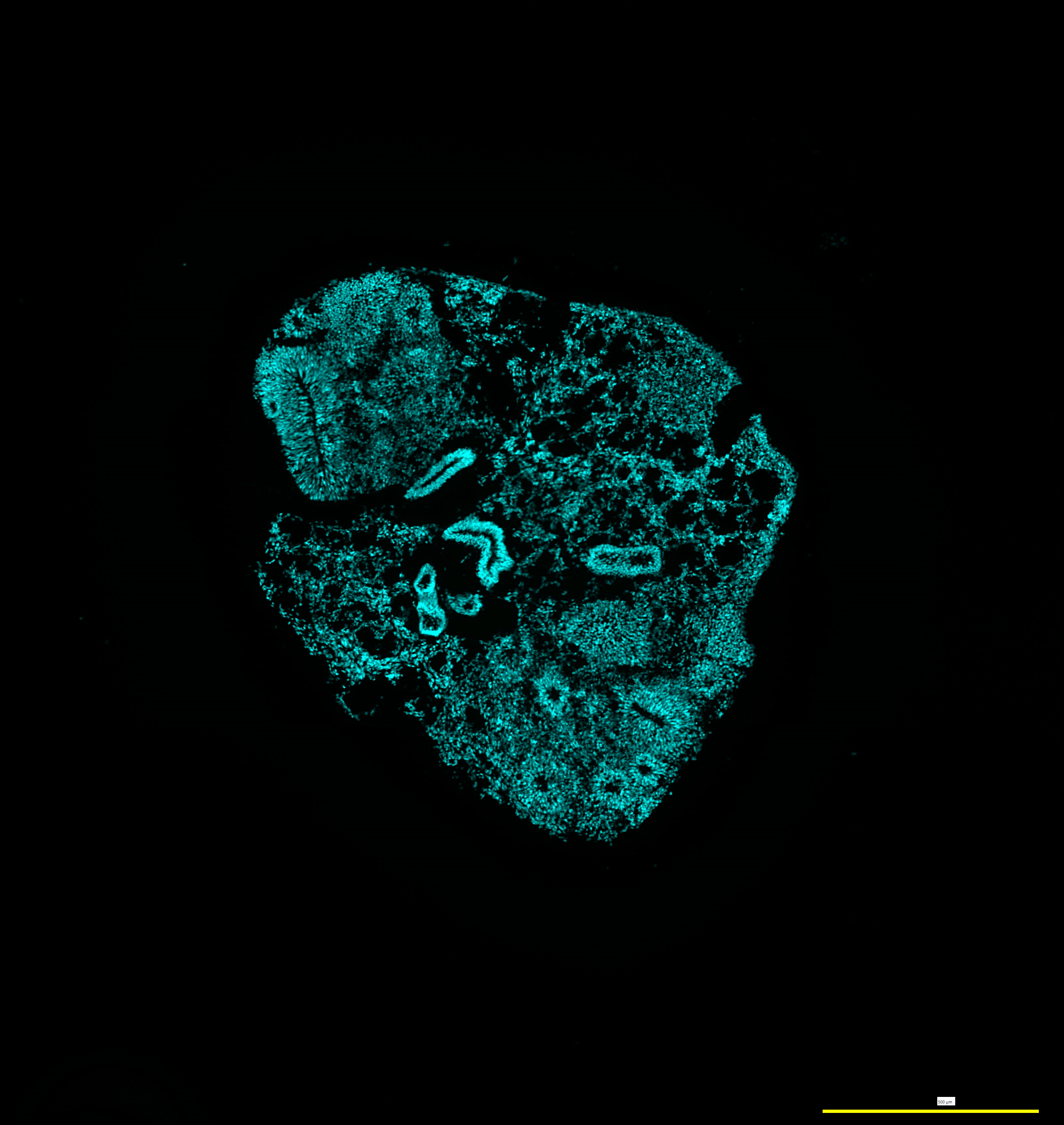

Supplement: Supplementary file 19 — Source data Fig. 7 [file 44319_2025_647_MOESM19_ESM.zip › Figure 7/7A/CRTDi011-AΓÇômutACTB-2_d29_DAPI.tif]

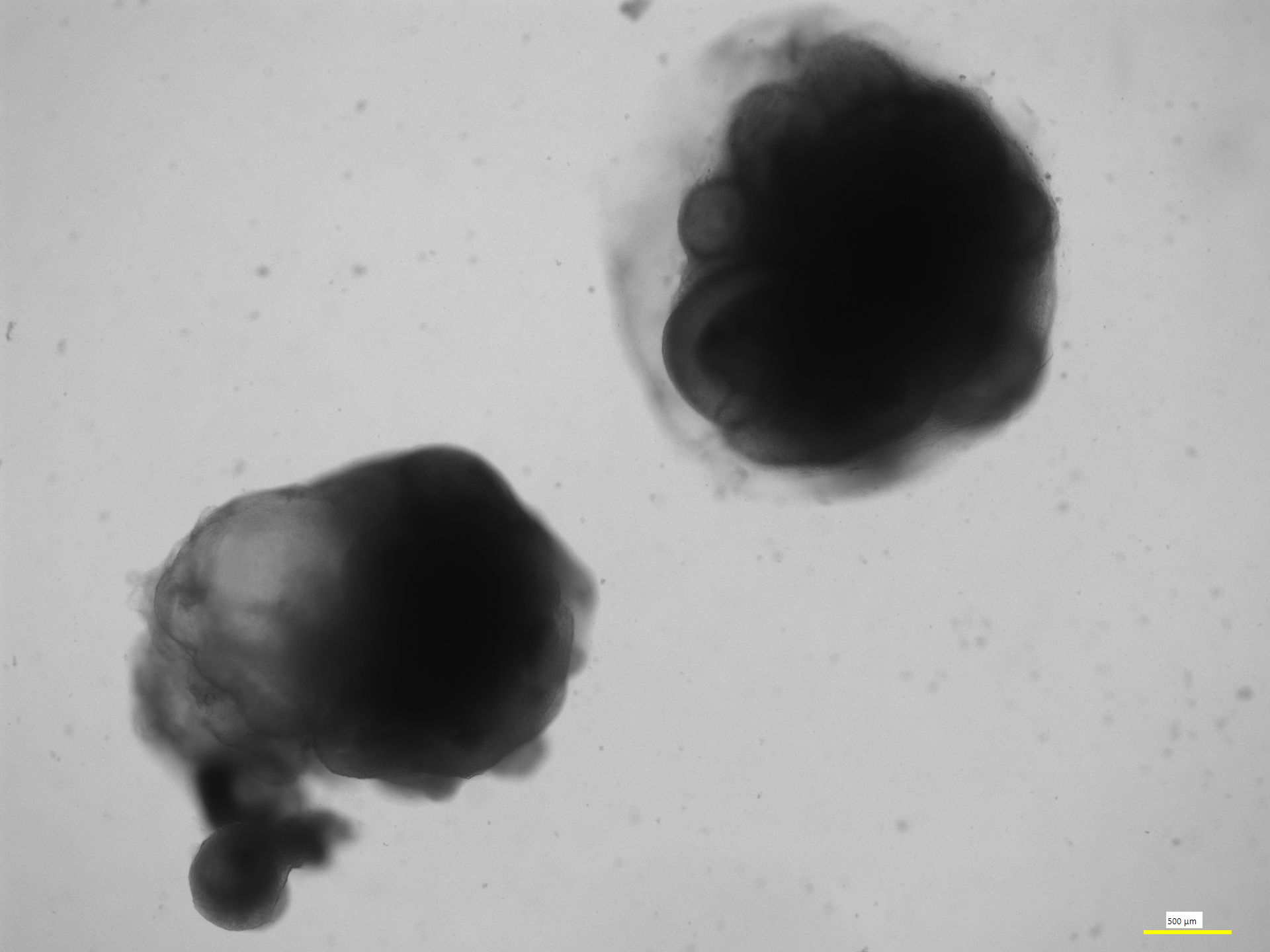

Supplement: Supplementary file 19 — Source data Fig. 7 [file 44319_2025_647_MOESM19_ESM.zip › Figure 7/7A/c2_CRTDi011-A_d29_brightfield.tif]

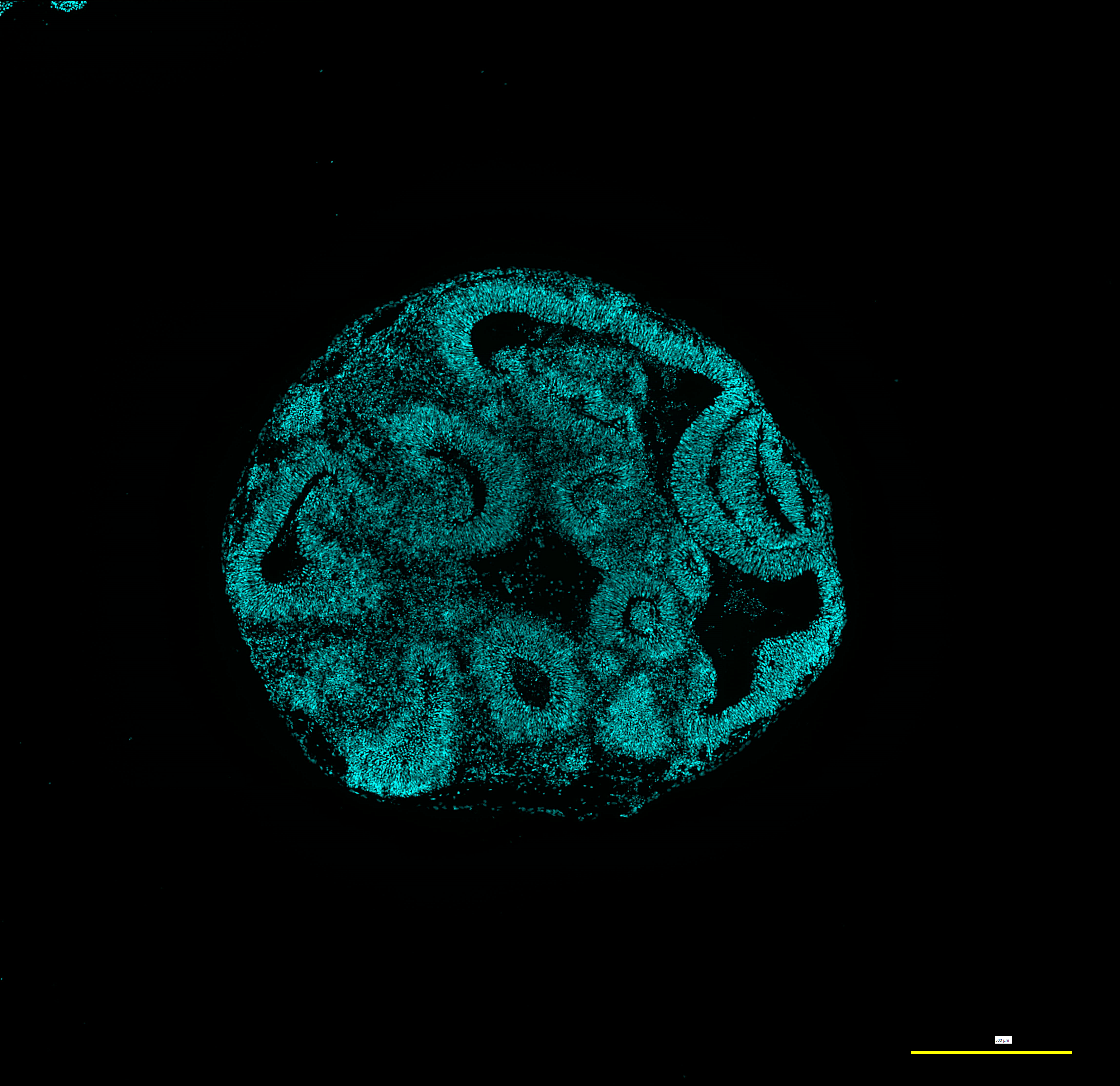

Supplement: Supplementary file 19 — Source data Fig. 7 [file 44319_2025_647_MOESM19_ESM.zip › Figure 7/7A/c2_CRTDi011-A_d29_DAPI.tif]

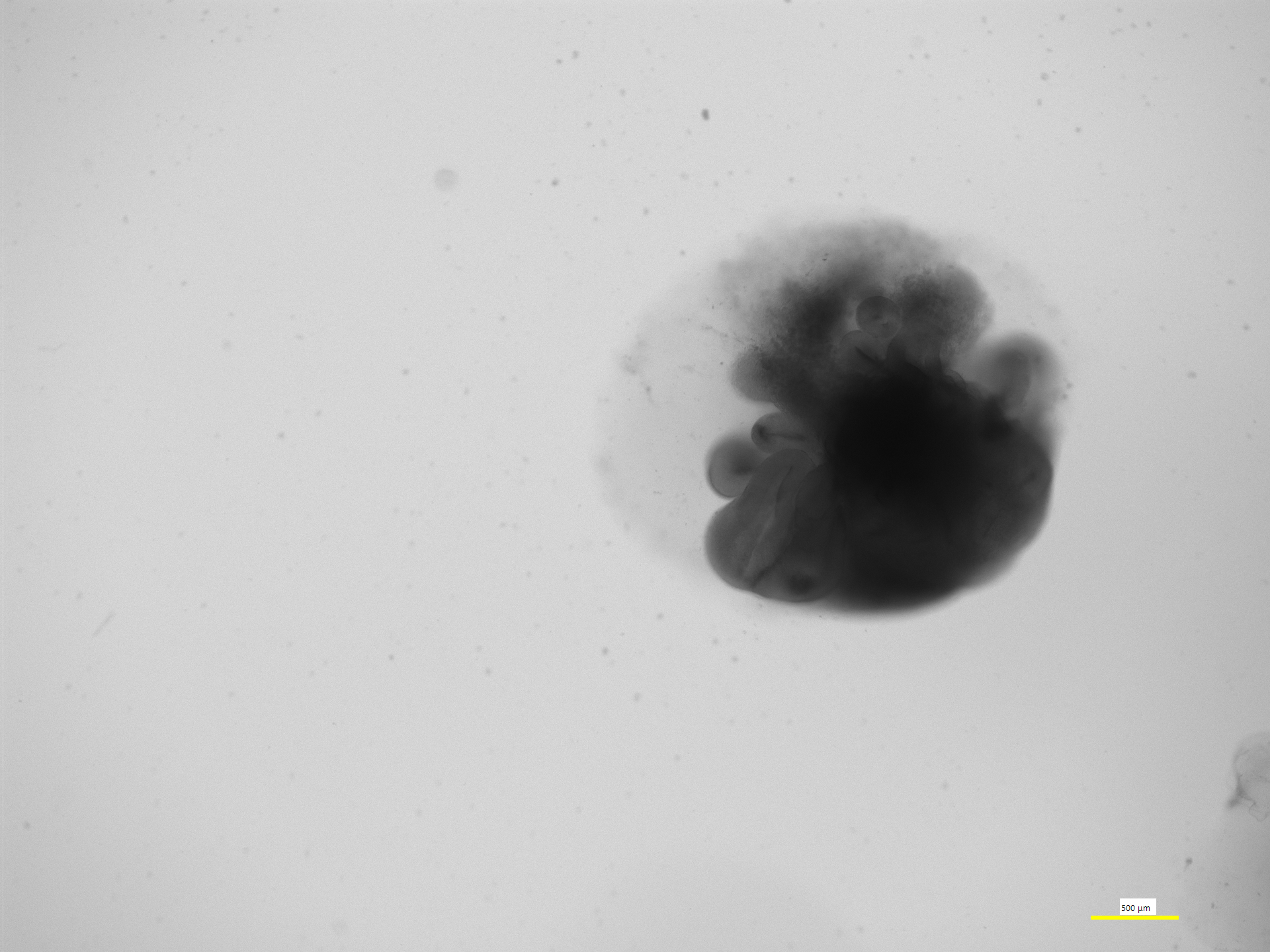

Supplement: Supplementary file 19 — Source data Fig. 7 [file 44319_2025_647_MOESM19_ESM.zip › Figure 7/7A/CRTDi011-AΓÇômutACTB-2_d29_brightfield.tif]
