# Supplementary material for: Cerebral organoids expressing mutant actin genes reveal cellular mechanism underlying microcephaly
Source: EMBO Rep. 2025 Dec 10;27(2):387–415. doi: 10.1038/s44319-025-00647-7 (PMC12852704; doi:10.1038/s44319-025-00647-7)
Supplement: Supplementary file 20 — Expanded View Figures [file 44319_2025_647_MOESM20_ESM.pdf]

Expanded View Figures

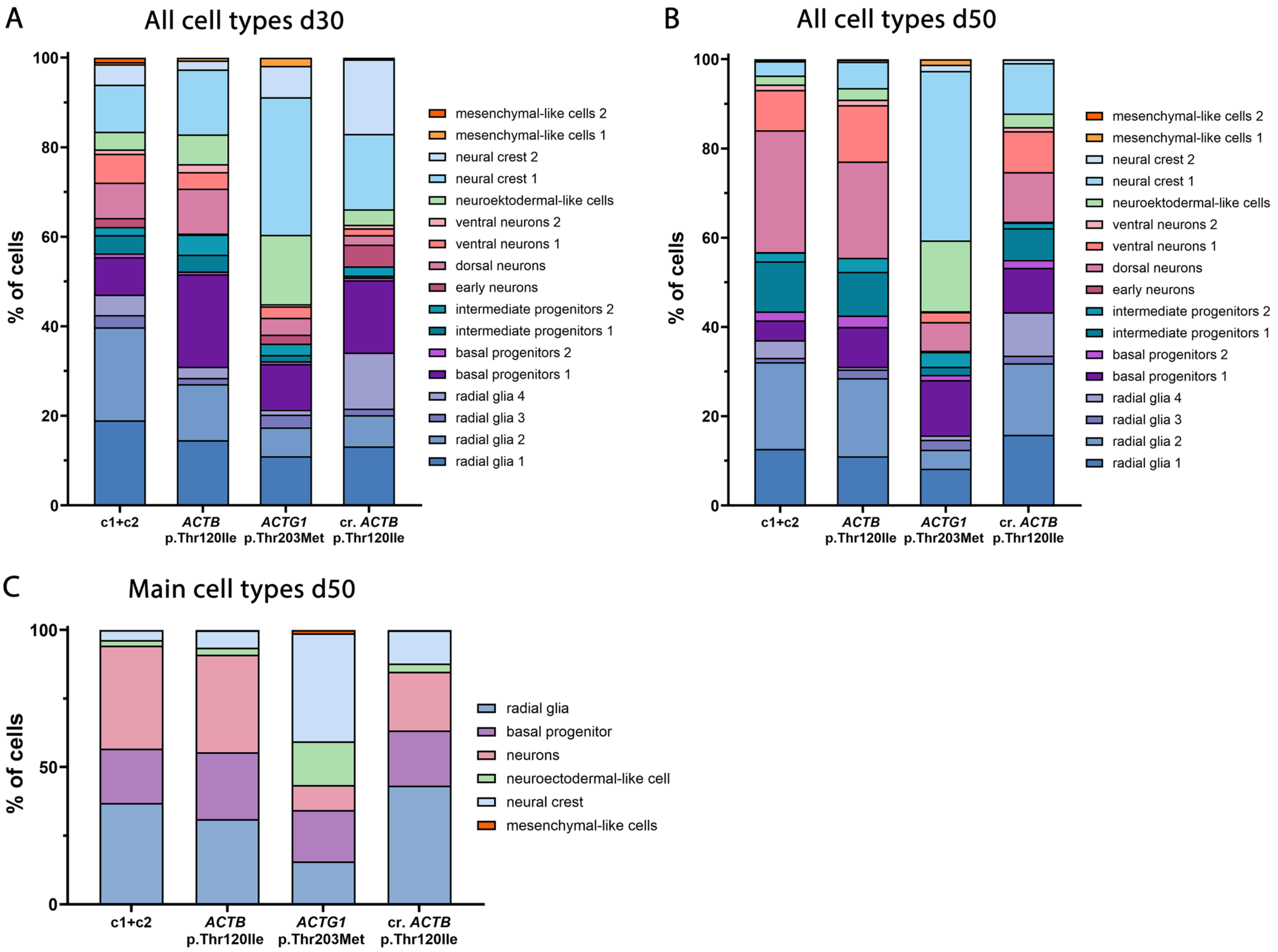

Figure EV1. Cell type composition of control and BWCFF-S cerebral organoids on culture day 30 and 50.

(**A**, **B**) Quantification of the cell type composition in percent of scRNA-seq data from control (c1, SC102A-1 and c2, CRTDi011-A;), BWCFF-S *ACTB* Thr120Ile, BWCFF-S *ACTG1* Thr203Met, and cr. *ACTB* Thr120Ile cerebral organoids at culture day 30 (**A**) and 50 (**B**). Specification of the color-coded cell types on the right side of the panel. (**C**) Quantification of the composition of the combined cell types (i.e., radial glia, basal progenitors, neurons, neuroectodermal-like cells, neural crest and mesenchymal-like cells) in percent of scRNA-seq data from control (c1, SC102A-1 and c2, CRTDi011-A;), BWCFF-S *ACTB* Thr120Ile, BWCFF-S *ACTG1* Thr203Met, and cr. *ACTB* Thr120Ile cerebral organoids at culture day 50. Specification of the color-coded cell types on the right side of the panel.

© The Author(s)

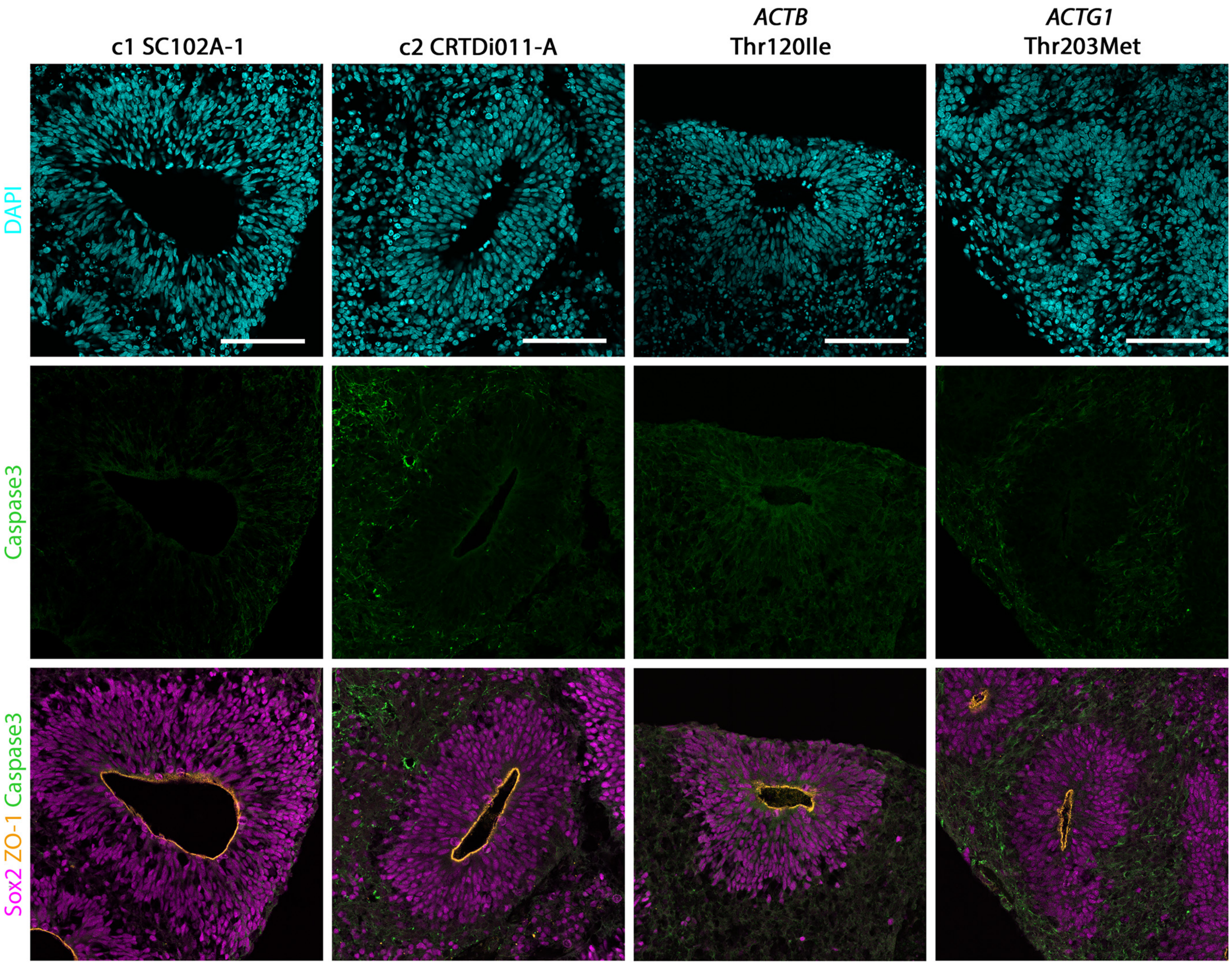

Figure EV2. No difference in apoptosis between control and BWCFF-S cerebral organoids.

Triple immunofluorescence for Caspase 3 (green), SOX2 (magenta) and ZO-1 (orange), combined with DAPI staining (blue), of sections of control (c1, SC102A-1 and c2, CRTDi011-A; two left columns), BWCFF-S *ACTB* Thr120Ile (second column from right) and BWCFF-S *ACTG1* Thr203Met (right column) 30-day-old cerebral organoids showing ventricle-like structures. Scale bars, 100 μm.

 © The Author(s)

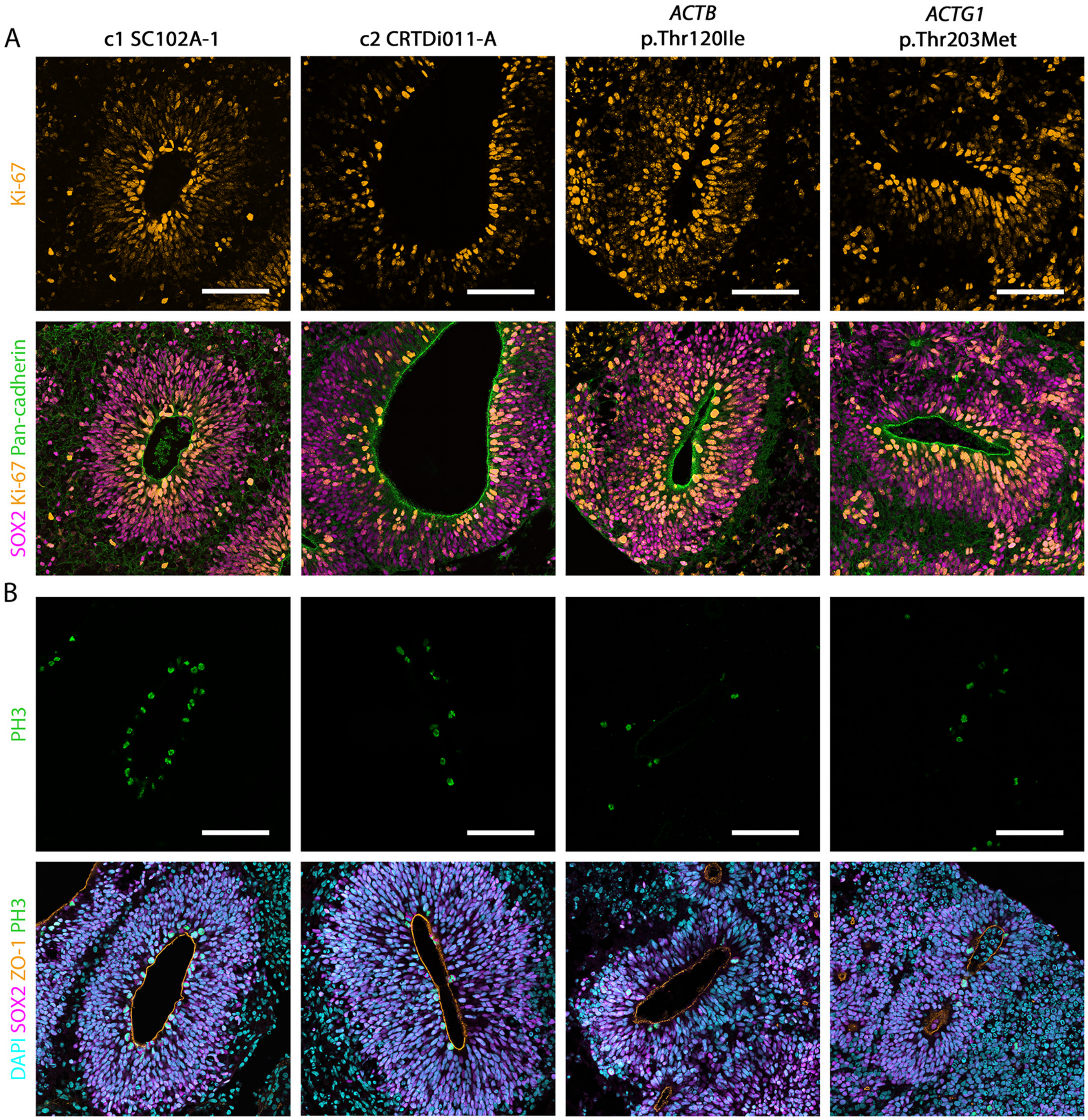

Figure EV3. No difference in the number of cycling VZ progenitors and mitotic APs between control and BWCFF-S cerebral organoids.

(**A**) Triple immunofluorescence for Ki-67 (orange), SOX2 (magenta) and pan-cadherin (green) of sections of control (c1, SC102A-1 and c2, CRTDi011-A; two left columns), BWCFF-S *ACTB* Thr120Ile (second column from right) and BWCFF-S *ACTG1* Thr203Met (right column) 30-day-old cerebral organoids showing ventricle-like structures. Scale bars, 100 µm. (**B**) Triple immunofluorescence for SOX2 (magenta), phosphohistone H3 (PH3, green) and ZO-1 (orange), combined with DAPI staining (blue), of sections of control (c1, SC102A-1 and c2, CRTDi011- A; two left columns), BWCFF-S *ACTB* Thr120Ile (second column from right) and BWCFF-S *ACTG1* Thr203Met (right column) 30-day-old cerebral organoids showing ventricle-like structures. Scale bars, 100 µm.

© The Author(s)

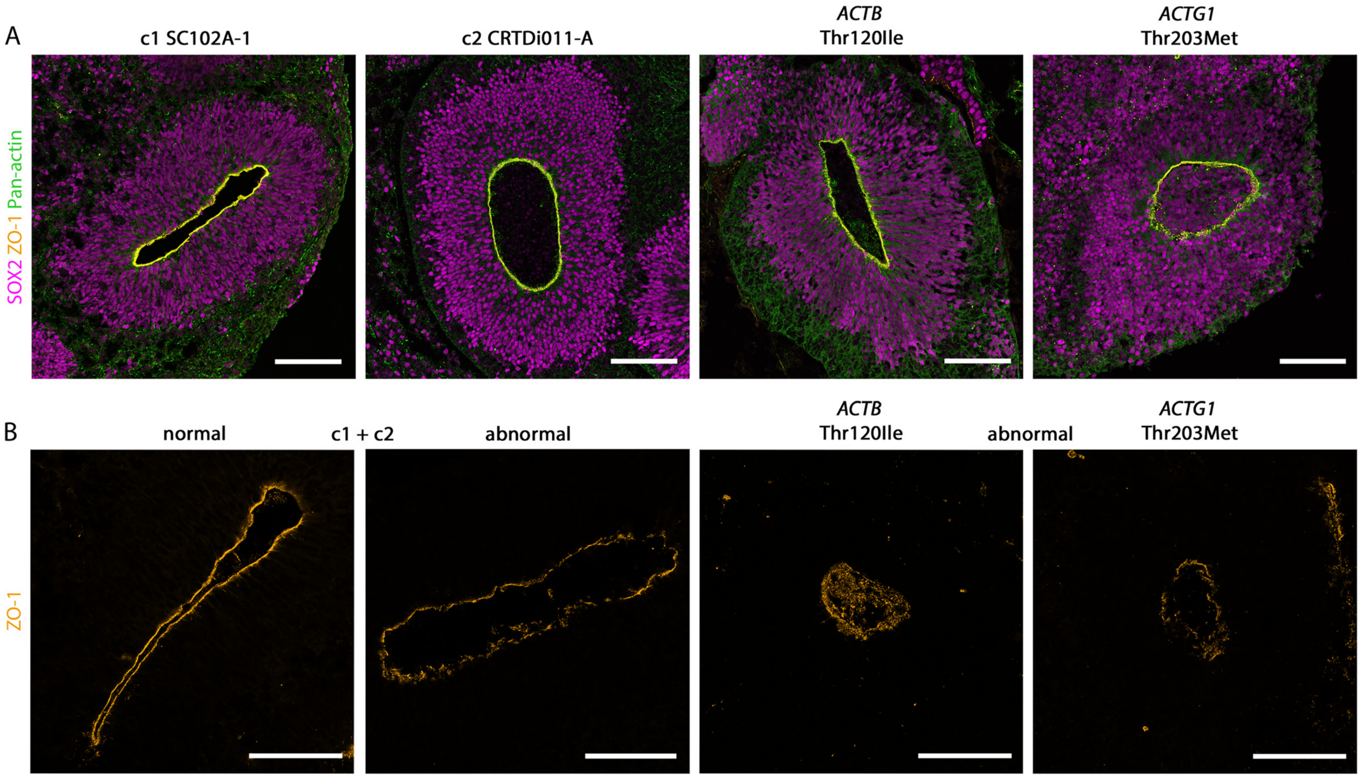

Figure EV4. Morphology of apical junctional complexes in control and BWCFF-S cerebral organoids.

(**A**) Triple immunofluorescence for SOX2 (magenta), ZO-1 (orange) and pan-actin (green) of sections of control (c1, SC102A-1 and c2, CRTDi011-A; two left panels) and BWCFF-S *ACTB* Thr120Ile (second panel from right) and *ACTG1* Thr203Met (right panel) 30-day-old cerebral organoids; Scale bars, 100 µm. (**B**) Exemplary images of ZO-1 immunofluorescence of sections of control (c1, SC102A-1 and c2, CRTDi011-A; two left panels), BWCFF-S *ACTB* Thr120Ile (second panel from right) and *ACTG1* Thr203Met (right panel) 30-day-old cerebral organoids showing normal (left panel) and abnormal (second, third and fourth panel from left) adherent junction belt morphology. Scale bars, 100 µm.

 © The Author(s)

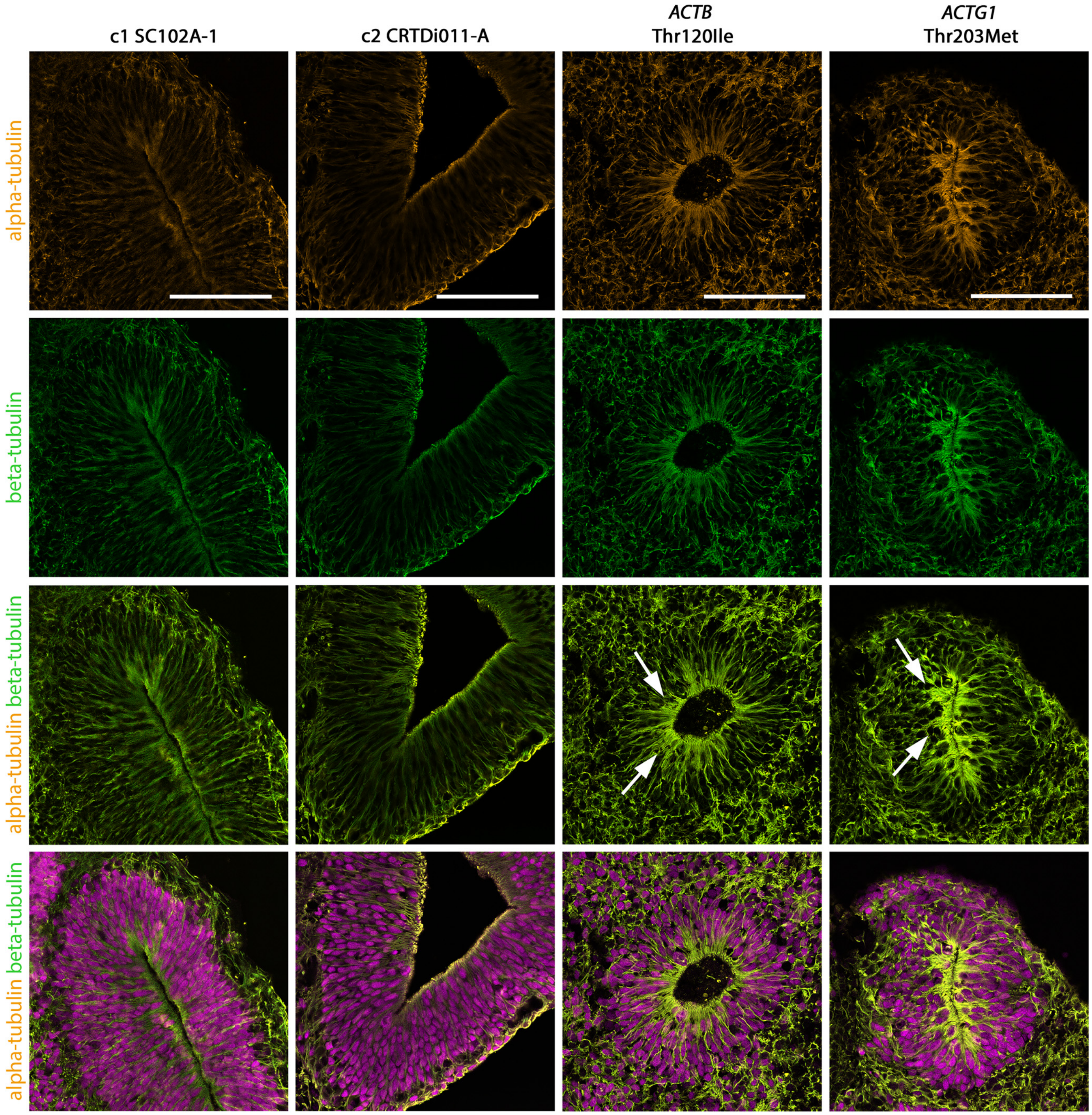

Figure EV5. BWCFF-S cerebral organoids show an increased localization of alpha- and beta-tubulin at the apical cell cortex.

Triple immunofluorescence for alpha-tubulin (orange, shown in rows 1, 3 and 4), beta-tubulin (green, shown in rows 2–4) and SOX2 (magenta, shown in row 4) of control (c1, SC102A-1 and c2, CRTDi011-A; two left columns), BWCFF-S *ACTB* Thr120Ile (second column from right) and BWCFF-S *ACTG1* Thr203Met (right column) 30-day-old cerebral organoids. Note the strong alpha-tubulin and beta-tubulin fluorescence signal at the apical cell cortex of BWCFF-S VZ progenitors (white arrows); Scale bars, 100 μm.

© The Author(s)
